# Book of Abstracts of the 21th European Conference on Eye Movements in Leicester 2022

**DOI:** 10.16910/jemr.15.5.2

**Published:** 2022-08-21

**Authors:** Victoria McGowan, Ascensión Pagán, Kevin B. Paterson, David Souto, Rudolf Groner

**Affiliations:** Chief Editor, Journal of Eye Movement Research; University of Bern

**Keywords:** eye movements, eye tracking, reading attention, Art perception, fixation, saccade

## Abstract

Contents

Keynotes:

*Iain Gilchrist:* Integrative Active Vision p 5

*Ziad Hafed:* A Vision for orienting in Primate Oculomotor Control Circuitry p 6

*Fatema Ghasia:* Miniscule Eye Movements Play a Major Role in Binocular Vision Disorders p.7

*Miriam Spering:* Eye Movements as a Window into Human Decision-Making p.8

*Monica S. Castelhano:* Explorations of how Scene Context and Previous Experience Dynamically Influence Attention and Eye Movement Guidance p.9

Symposia:

Eye Tracking and the Visual Arts p.19

Eye Movements during Text Processing and Multiline Reading p.23

Unstable Fixation and Nystagmus with a Focus on the Next Generation of Researchers p.84

Eye Movements as a measure of Higher-Level Text Processing p.97

Eye Movements in Memory Processes Between Working Memory and Long-Term Memory p.178

Symposium to Honour Alexander Pollatsek’s Legacy to Eye Movement Research p.204

Talks:

Reading p.30

Parafoveal Processing p.36

Cinical and Applied p.39

Visual Search p.92

Eye Movement Control in Reading I & II p.104 & 116 & 225

Reading Development p.110

Decision-Making p.122

Eye-tracking Methods p.128

Real World and Virtual Reality p.134

Chinese Reading p.185

Special Populations p.191

Visuo-motor p.195

Bilingual Reading p.201 & 217

Reading Comprehension p.219

Pupillometry p.235

Poster sessions:

Attention p.44 & 139

Cognition p. 49

Visuo-Motor p.62

Memory p.145

Methods p.150

Reading p. 57 & 155

Real World p.169

Social Cognition p.173

## Book of Abstracts of the 21th European Conference on Eye Movements in Leicester 2022



