# Supplementary material for: Book of Abstracts of the 21th European Conference on Eye Movements in Leicester 2022
Source: J Eye Mov Res. 2022 Aug 21;15(5):10.16910/jemr.15.5.2. doi: 10.16910/jemr.15.5.2 (PMC10350687; doi:10.16910/jemr.15.5.2)
Supplement: Supplementary file 1 [file jemr-15-05-b-SD1-01.pdf]

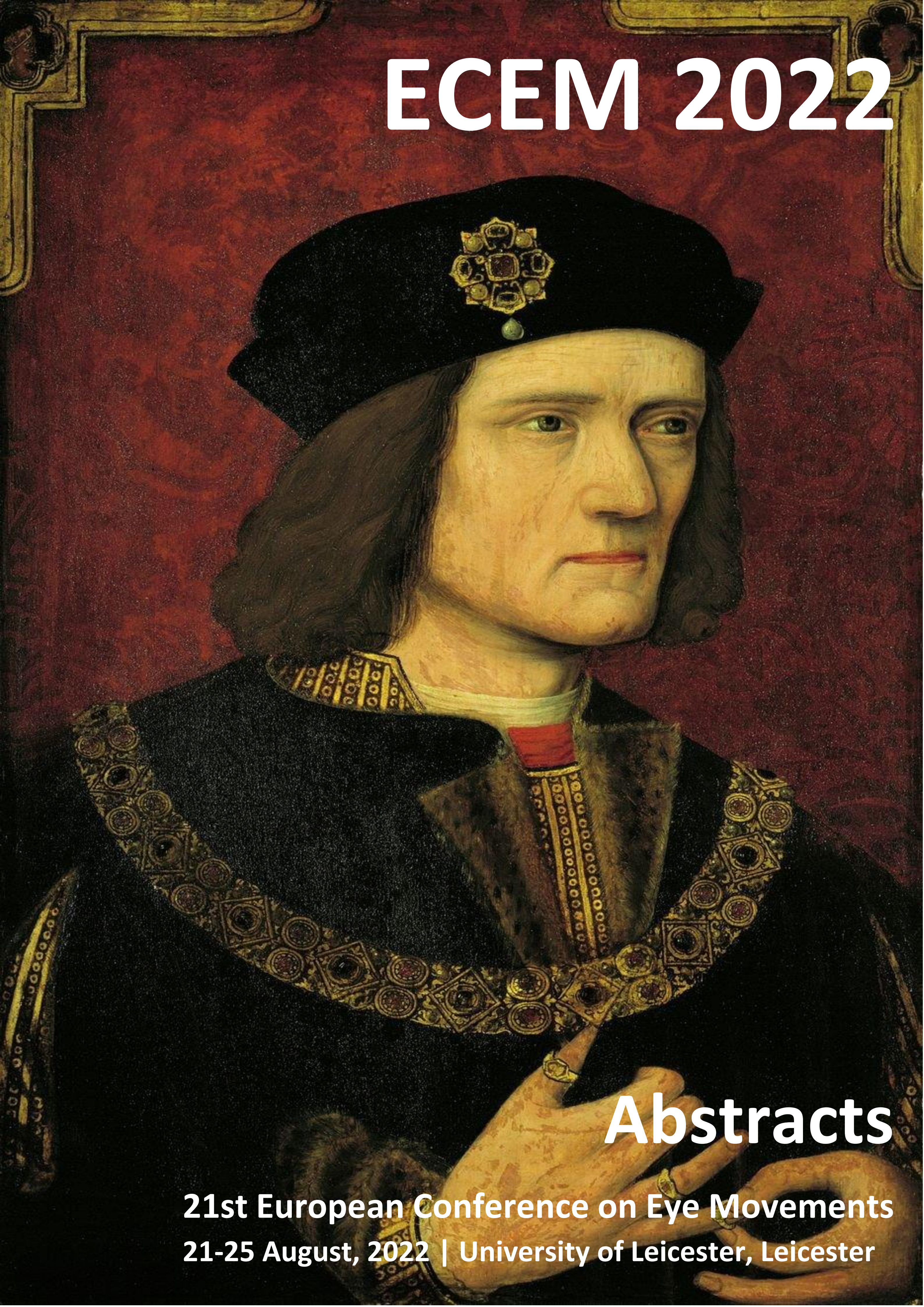

ECEM 2022

Abstracts

21st European Conference on Eye Movements

21-25 August, 2022 | University of Leicester, Leicester

Programme
# of the 21st European Conference
on Eye Movements

Edited by Victoria A. McGowan, Ascensión Pagán,

Kevin B. Paterson, and David Souto

August 21st to 25th, 2022

Leicester, UK

About the ECEM Programme Cover Image

The background of the front cover image depicts a portrait of King Richard III. King Richard III ruled from 1483-1485 and was the last English king to die in battle, at the battle of Bosworth Field (just outside Leicester). In 2012 an ambitious team from the University of Leicester set out to discover the remains of his body - and found them buried under a car park in Leicester city centre. The discovery and identification of his body is now one of the University's most celebrated achievements. For more information, see: https://le.ac.uk/richard-iii

The painting is by an unknown artist and is part of the Royal Collection. Examinations of the panel on which the portrait was painted suggests it was created around a date between 1504 and 1520. It also has been suggested that careful examination of the paintings by eye movements alone can reveal some deliberate alteration to the painting, with changes to right shoulder and coat used to create an unevenness of the shoulders. There is also the suggestion that the eyes have been overpainted to appear greyer, and that the mouth has been changed to turn down at the corners.

These changes most likely reflect propogandist attempts to portray Richard unfavourably. The King had scoliosis of the spine, and the physical difference this produced has been widely used (including by Shakespeare) to signal moral failings and to characterise Richard as an evil man.

For more information, see the Royal Collection Trust

https://www.rct.uk/collection/403436/richard-iii-1452-85

Contents

Keynotes 5

Monday, August 22 10

LT1 10.00-12.00 Higher-level I / Social Cognition 10

LT1 13.00-15.00 Symposium: Eye-tracking and the Visual Arts 16

LT2 10.00-12.00 Symposium: Eye Movements during Multi-line Reading 23

LT2 13.00-15.00 Reading 30

LT2 16.30-17.30 Parafoveal Processing 36

LT8 16.30-17.30 Clinical and Applied I 40

Poster Session I 45

Tuesday, August 23 86

LT1 10.00-12.00 Symposium: Unstable Fixation and Nystagmus 86

LT1 13.00-15.00 Visual Search 90

LT1 16.30-18.30 Symposium: Eye Movements & Higher-level Text Processing 97

LT2 10.00-12.00 Eye Movement Control in Reading 104

LT2 13.00-15.00 Reading Development 110

LT2 16.30-18.30 Eye Movement Control in Reading II 116

LT8 10.00-12.00 Decision-making 122

LT8 13.00-15.00 Eye-tracking Methods 128

LT8 16.30-17.30 Real World and Virtual Reality 134

Poster Session II 139

Wednesday, August 24 179

LT1 10.00-12.00 Eye Movements in Memory Processes 179

LT2 10.00-12.00 Chinese Reading 185

LT2 13.00-14.00 Special Populations 191

LT8 10.00-12.00 Visuo-motor 195

LT2 13.00-14.00 Bilingual Reading 201

Thursday, August 25 204

LT1 09.30-12.00 Symposium: To honour Alexander Pollatsek's Legacy 204

LT1 13.00-15.00 Higher-level II 213

LT1 15.30-16.30 Bilingual Reading II 217

LT2 13.00-15.00 Reading Comprehension 219

LT2 15.30-16.30 Eye Movement Control in Reading III 225

LT8 13.00-15.00 Clinical and Applied II 228

LT2 15.30-16.30 Pupillometry 235

Author Index 238

Note

Keynote, talks and poster abstracts are listed separately in chronological order and by venue for parallel symposia and thematic sessions. Only the affiliation and email address of the first author is included. For the full list of affiliations see the online program: https://easychair.org/smart-program/ECEM2022/

An Author Index, included after the abstracts, can be used to retrieve specific author contributions.

KEYNOTE

PETER WILLIAMS LECTURE THEATRE - 17:30- 18:30

Integrative Active Vision

Iain Gilchrist

University of Bristol, United Kingdom
psidg@bristol.ac.uk

The Active Vision framework (Findlay and Gilchrist, 2003) argued that eye movement will be central to any successful model of visual perception and cognition: vision is about looking and seeing.

The study of eye movements, and particularly saccadic eye movements, is one of the great success stories of cognitive neuroscience. The brain networks that supports the generation of saccadic eye movements has been studied in detail for over fifty years and we have a deep understanding of the relationship between behaviour, anatomy and neurophysiology.

However, psychologist and neuroscientist tend to study a single systems in isolation. In this talk I will argue for the need to understand how the saccadic network interacts with other brain systems and functions. I will review examples of our own research that attempts to address this issue by exploring the relationship between the saccadic system and face processing, reward, response timing and salience. Together these studies describe a rich and complex pattern of interactions.

Any successful model of eye movement behaviour will ultimately need to include the influence of the multiple processes that shape visual perception and cognition. Integrative Active Vision is about understanding how different brain systems and processes work in concert with the eye movement systems to generate integrated, coherent and complex behaviour.

KEYNOTE

BENNETT LECTURE THEATRE 1 - 17:30- 18:30

A Vision for orienting in Primate Oculomotor Control Circuitry

Ziad Hafed

Werner Reichardt Centre for Integrative Neuroscience, Germany, Germany
ziad.m.hafed@cin.uni-tuebingen.de

Movement control is critical for successful interaction with our environment. However, movement does not occur in complete isolation of sensation, and this is particularly true of eye movements. Here, the superior colliculus (SC) plays a fundamental role, issuing saccade motor commands in the form of strong peri-movement bursts that are widely believed to specify both saccade metrics (direction and amplitude) and kinematics (speed). The lower brainstem, in turn, transforms these commands into appropriate extra-ocular muscle drives. In this talk, I will describe how the existence of visual sensory responses in the SC and brainstem oculomotor control networks is critical for supporting orienting, as well as for coordinating rapid orient-versus-interrupt decisions that we are constantly faced with in a dynamic environment. The series of investigations that I will describe will culminate in the intriguing observation that classic SC saccade-related peri-movement bursts are clearly dissociated from movement kinematics; rather, they are sensory-tuned and contain information about the visual features of the saccade targets. The visual signals that we observe are also often the strongest for images of real-life objects, rather than simplified patterns. These results recast classic models of brainstem oculomotor control, as well as hierarchical cortico-centric views of visual image processing.

KEYNOTE

PETER WILLIAMS LECTURE THEATRE - 8:30- 9:30

Miniscule Eye Movements Play a Major Role in Binocular Vision Disorders

Fatema Ghasia

Cleveland Clinic, United States
fatemaghasia@gmail.com

Dr. Fatema Ghasia is clinician-scientist with expertise in pediatric ophthalmology and binocular vision disorders and research interests in systems neuroscience, with emphasis on human and primate ocular motor control. She is Associate Professor and directs the Vision Neurosciences and Ocular Motility Laboratory at Cole Eye Institute, Cleveland Clinic. My lab's primary focus is to understand the role of abnormal neural circuits in strabismus and amblyopia and apply novel strategies for their treatment. As a pediatric ophthalmologist, I witness first-hand the problems and nuances associated with diagnosing and treating patients with binocular vision disorders. As an oculomotor scientist, I have discovered and realized the value of obtaining eye movement recordings in these patients. To resolve a desperate need that I experienced as a clinician, I leveraged my role as an eye movement scientist to understand fixation eye movement abnormalities as they relate to amblyopia diagnosis and treatment outcomes. We have built a cutting-edge infrastructure for tracking eye and head movements simultaneously with high accuracy and precision in children under different viewing conditions. Over the last several years, we have investigated the utility of eye movement measurements in children with binocular vision disorders. The systematic analysis of eye movement traces obtained in the lab has revealed for the first time several features that can be utilized to detect the presence of amblyopia, clinical types, and severity. We have also found that FEM abnormalities correlate with reduced contrast sensitivities and depth perception, and inter-ocular suppression experienced by these patients. We have also found that assessing FEM characteristics can be a valuable tool to predict functional improvement after patching therapy and recent data as it relates to newer amblyopia dichoptic treatments.

KEYNOTE

PETER WILLIAMS LECTURE THEATRE - 8:30- 9:30

Eye Movements as a Window into Human Decision-making

Miriam Spering

University of British Columbia, Canada
mspering@mail.ubc.ca

Seeing and perceiving the visual world is an active and multimodal process during which the eyes continuously scan the visual environment to sample information. My research group uses human eye movements as sensitive indicators of performance in real-world interceptive tasks. Tasks such as catching prey or hitting a ball require prediction of an object's trajectory from a brief glance at its motion, and an ultrafast decision about whether, when and where to intercept. I will present results from two research programs that use eye movements as a readout of these types of decision processes. The first series of studies investigates go/no-go decision making in healthy human adults and baseball athletes and reveals that eye movements are sensitive indicators of decision accuracy and timing. The second set of studies probes decision making in patients with motor deficits due to Parkinson's disease and shows differential impairments in visual, motor and cognitive function in these patients. I will conclude that eye movements are both an excellent model system for prediction and decision making, and an important contributor to successful motor performance.

KEYNOTE

BENNETT LECTURE THEATRE 1 - 17:30- 18:30

Explorations of how Scene Context and Previous Experience Dynamically Influence Attention and Eye Movement Guidance

Monica S. Castelhano

Queen's University, Canada
monica.castelhano@queensu.ca

Research has long established that scene context improves performance across a number of tasks by affecting both attention and memory processing. Scene context improves performance by providing some form of predictions and expectations about the state of the world. However, many recent studies have also shown that immediate previous experience plays a significant role in attentional guidance. Thus, expectations about the world can be established in a number of ways, from the assumptions that come with real-world scene schemas to the previous experience from previous trials. Here, I will discuss how expectations affect performance when considered across different timelines and will explore how different influences dynamically modulate attentional guidance. By examining changes in strategies while viewing, we may come to a better understanding of how attentional priorities dynamically shift over the short-term and long-term.

HIGHER-LEVEL / SOCIAL COGNITION

BENNETT LECTURE THEATRE 1 - 10:00- 10:20

The influence of action affordances and visual salience on viewing of ancient stone tools

Timothy Hodgson, Maria Silva Gago, Flora Ioannidou and Emiliano Bruner

University of Lincoln School of Psychology, United Kingdom
tlhodgson@lincoln.ac.uk

The development of stone tools is thought to parallel the evolution of human cognitive and attentional capacities. One possibility is that sophisticated tool production techniques developed alongside brain systems that process action relevant features of natural objects. In a series of experiments, we investigated eye movements and visual attention during the viewing of Palaeolithic stone tools. Participants were presented with images of hand-axes, roughly worked pebbles, unworked pebbles and hi-bred images combining a hand-axe outline with an unworked stone texture. Images were also analysed using a graph based visual salience algorithm in order to identify which components of the image were most visually conspicuous as opposed to functionally relevant. Overall, the findings suggest that visual exploration is directed towards functional aspects of tools such as the grasping point at the base and the knapped cutting surface rather than salient internal and external features of the tool highlighted by the saliency model. A follow-up study used "real-world" mobile eye tracking during handling of the same stone objects and found an identical distribution of fixation dwell times across the tool regions. It is concluded that functional aspects of Palaeolithic tools strongly influence viewing patterns over and above the visual salience of images features.

HIGHER-LEVEL I / SOCIAL COGNITION

BENNETT LECTURE THEATRE 1 - 10:20- 10:40

Exploring the Mechanisms Related to Attention Biases for Threat in Social Anxiety

Katerina Pavlou, Athina Manoli, Julie Hadwin and Valerie Benson

Cyprus Institute of Marketing, CY, Cyprus
katerina@cima.ac.cy

Social anxiety disorder (SAD) is among the most common mental health disorders in children and SAD negatively affects quality of life. Cognitive models of anxiety propose that attentional biases toward threatening information play etiological and maintenance roles in anxiety. Threat processing is related to automatic attentional orienting to threat, difficulty disengaging from task-irrelevant threatening stimuli, and vigilance-avoidance of threat. The current study examined attentional processing in relation to SAD symptoms in N = 38 children (25 males) aged between 9-11 years old. We employed the Remote Distractor Paradigm (RDP) and presented faces that displayed different expressions (happy, angry, neutral) to act as distractors in a task where participants were required to ‘look at’ a non-face target presented at a lateral location. Distractors could appear at central, parafoveal and peripheral eccentricities, and we recorded and analysed eye movements for the task. Higher symptoms of SAD were associated with more saccadic errors for angry compared to happy face distractors, and slower latencies to the target in the presence of angry compared to happy and neutral face distractors, regardless of the distractor eccentricity. The findings indicate that social anxiety is characterised both by orienting to threat and difficulty disengaging from task-irrelevant threat.

HIGHER-LEVEL I / SOCIAL COGNITION

BENNETT LECTURE THEATRE 1 - 10:40- 11:00

Gaze path category differences lie in early fixation locations

Radha Nila Meghanathan and Stefan Pollmann

Otto von Guericke University Magdeburg, Germany
radha.meghanathan@ovgu.de

In an earlier study, we found that the fusiform face area and parahippocampal place area were activated not only by viewing face and house images, but also by gaze paths corresponding to face and house viewing. While viewing a face, we look at specific regions of the face in a specific sequence. How these viewing patterns of faces differ from those of houses is not known. To study differences in gaze paths between categories, we compared viewing patterns of faces with houses and a third category, leaves, by recording eye movements while participants viewed face, house and leaf images. We found that locations, but not durations, of fixations contributed toward identifying the three categories using a classifier. Distinguishing face from house gaze paths had higher accuracy than distinguishing house from leaf gaze paths or face from leaf gaze paths. We also found that early fixations were better predictors of category compared to later fixations. The second and third fixations contributed most to category identification. The differences between the gaze paths associated with these three categories arise early and lie in the locations of the fixations.

HIGHER-LEVEL I / SOCIAL COGNITION

BENNETT LECTURE THEATRE 1 - 11:00- 11:20

Gaze and speech behavior in parent-child interactions: A dual eye-tracking study

Gijs Holleman, Ignace T. C. Hooge, Jorg Huijding, Maja Deković, Chantal Kemner and Roy S. Hessels

Utrecht University, Netherlands
g.a.holleman@uu.nl

In this study, we used a dual eye-tracking setup to investigate face scanning behavior during video-mediated interactions between parents and their preadolescent children (8-10 years). 81 parent-child dyads engaged in two brief conversations about cooperative and conflictive family topics. We used eye tracking, audio-, and video-recordings to assess what regions of the face are looked at during episodes of speaking and listening, and whether patterns of gaze and speech were influenced by the topic of conversation. Regarding speech, our results show that children spoke more in the cooperation-scenario whereas parents spoke more in the conflict-scenario. Regarding gaze, we found that both parents and children looked more at the other's mouth region while listening compared to while speaking, which converges with previous studies about face scanning during speech perception. Furthermore, we found that parents gazed slightly more at the eyes of their children in the conflict-scenario compared to the cooperation-scenario. Results are discussed in terms of the role that parents and children take during cooperative and conflictive interactions, and how gaze behavior may support and coordinate such interactions.

HIGHER-LEVEL I / SOCIAL COGNITION

BENNETT LECTURE THEATRE 1 - 11:20- 11:40

Looking for interaction? An eye-tracking study on brief social encounters

Roy S. Hessels, Jeroen S. Benjamins, Andrea van Doorn, Jan Koenderink, Gijs Holleman and Ignace T. C. Hooge

Utrecht University, Netherlands
R.S.Hessels@uu.nl

As humans move through parts of their environment, they meet others that may or may not try to interact with them. Where do people look when they meet others? Does one's looking behavior depend on the other person's behavior, and does one's looking behavior predict subsequent interaction? We equipped 23 participants with a wearable eye tracker and had them walk through a university building. On the way, they encountered nine "walkers". Walkers were instructed to e.g., ignore the participant, greet him or her, or attempt to hand out a flyer. Participants' gaze behavior was analyzed using a fully automated area-of-interest analysis based on OpenPose pose estimation. The participant's gaze was mostly directed to the currently relevant body parts of the walker. Thus, the participant's gaze depended on the walker's actions. Individual differences in participants' gaze were consistent across walkers, regardless of the walkers' behavior. Our findings are discussed in the light of existing theories of gaze allocation, which may be extended by taking social motivation into account.

HIGHER-LEVEL I / SOCIAL COGNITION

BENNETT LECTURE THEATRE 1 - 11:40- 12:00

Processing Visual Information in the Classroom – A Comparison of Teachers' Gaze During Different Didactic Activities

Leonie Telgmann and Katharina Müller

Leibniz University Hannover, Germany
leonie.telgmann@iew.uni-hannover.de

Teachers need to distinguish between irrelevant and relevant classroom information in order to meaningfully interpret and react to this information (Wolff et al., 2021). To understand teachers’ information processing in real classrooms, mobile eye-tracking is used to assess teachers´ visual attention (Stürmer et al., 2017; McIntyre et al., 2019). Since the direct comparison of diverse classroom lessons in real life poses challenges, we have limited knowledge about teachers’ visual focus of attention during different didactic activities. As these activities, however, place different demands on the teachers’ instructive and learning-supporting actions (i.e. verbal and non-verbal, including eye-movements), we developed a standardized classroom teaching simulation to examine novice teachers´ focus of attention during teacher- and student-led activities.

Gaze behaviour of N=14 novice teachers’ was recorded with Tobi Glasses 3 (50 Hz). Eye movement measures (i.e. fixation frequency, average fixation duration) for AOIs representing seven different learner profiles were analysed and compared for a teacher-, and student-led activity. Results show that novice teachers significantly differ in their fixation frequency on the student with active off-task behaviour during both activities. Additionally, we observe a slight tendency of novice teachers to distribute their attention more evenly while teaching teacher-led activities.

SYMPOSIUM: EYE-TRACKING AND THE VISUAL ARTS

BENNETT LECTURE THEATRE 1 - 13:00- 14:40

Chair: Anna Miscena

University of Vienna, Austria
anna.miscena@univie.ac.at

Symposium Organiser: Anna Miscenà (Universität Wien)

The experience of art has always defied quantification: not all forms of art are tangible, the meaning of art is understood as subjective, its value is often unpredictable. Hence the study of art and of its perception is often descriptive, interpretative, and theoretical.

Nonetheless, in recent time, the study of art has changed to include tools of empirical and quantitative investigation. Among these, eye-tracking has enabled us to tackle questions which have long been central in the fields of art history and aesthetics. Some of these questions concern art itself: which elements of a painting gather our attention? Do composition, style, colour, affect eye-movements? Other questions concern us, the spectators, and the context in which art is found: can we monitor differences between groups of viewers? How do curatorial choices shape the viewing experience?

In this Symposium, researchers with backgrounds in museology, art history, sociology and computer science begin to answer some of these questions, using eye-movements as a tool to gain innovative insights in the visual experience of art. By presenting a selection of recent studies, this Symposium aims to spark a debate on the methodological implications of studying art and its perception quantitatively and empirically.

SYMPOSIUM: EYE-TRACKING AND THE VISUAL ARTS

BENNETT LECTURE THEATRE 1 - 13:00- 13:20

Is it Art? Effects of Framing Images as Art Versus Non-Art on Gaze Behavior and Aesthetic Judgments

Frank Papenmeier, Gerald Dagit, Christoph Wagner and Stephan Schwan

Eberhard Karls Universität Tübingen, Germany
frank.papenmeier@uni-tuebingen.de

The perception and evaluation of art might be governed by both stimulus-driven aspects and expectations of the observer. By framing actual artwork and actual scientific depictions as being either artwork or scientific depictions, we were able to investigate the individual contributions of both factors on viewers' gaze behavior and aesthetic judgments. With Experiment 1, we identified a set of images that were equally likely classified as being either artwork or scientific depiction by art laypersons. In Experiment 2, we measured art laypersons' eye-movements as well as their aesthetic judgments regarding those images (half being actual artwork and half being actual scientific depictions). Importantly, we told one group of participants that all images were artwork, whereas we told another group of participants that all images were scientific depictions. Our results indicate that gaze behavior was influenced by the actual image type but not by framing. Actual artwork led to fewer and longer fixations, a shorter scanpath, a shorter distance of fixations to the screen center, and a lower similarity of viewing patterns across participants. Further, actual artwork was rated as being more complex but less meaningful than scientific depictions, with no differences regarding perceived structuredness and liking. Framing the images as artwork led to no changes in perceived meaningfulness, complexity, or structuredness but to lower ratings on liking. We conclude that while the dichotomy between art and non-art influenced art laypersons' gaze behavior and aesthetic judgments, this influence was primarily driven by bottom-up factors rather than a specific cognitive art schema.

SYMPOSIUM: EYE-TRACKING AND THE VISUAL ARTS

BENNETT LECTURE THEATRE 1 - 13:20- 13:40

Eye-Catchers in the Museum: Measuring the Attraction Potential of Single Artworks

Carola Korhummel, Luise Reitstätter and Raphael Rosenberg

University of Vienna, Austria
carola.korhummel@univie.ac.at

It is a common observation that visitors dedicate a relatively short time period to individual exhibits in relation to their total length of stay in the museum. However, some artworks are looked at significantly longer than others, while some are barely noticed at all. Likewise, some artworks tend to leave a lasting impression, whereas others are not remembered in particular. Intriguingly enough, eye-catching exhibits are often the same among different visitors. Based on an interdisciplinary mobile eye tracking study conducted by the Lab for Cognitive Research in Art History (CReA) of the University of Vienna in cooperation with the University of Tübingen, the EVAlab and the Austrian Gallery Belvedere in two successive years, this analysis combines quantitative and qualitative data in order to measure the attraction potential of artworks within an exhibition context. While previous studies have already pointed out the cumulative effect of exhibits, the focus thus shifts to the inherent potential of single artworks to capture the visitors' gazes.

SYMPOSIUM: EYE-TRACKING AND THE VISUAL ARTS

BENNETT LECTURE THEATRE 1 - 13:40- 14:00

Eye-tracking and Painting Restoration

Pablo Fontoura

EHESS, France
pablosfontoura@gmail.com

The effects of painting restoration on the visual perception of museum visitors

This proposal will present a method to understand the influence of painting conservation on museum visitors' perception, visual exploration, and aesthetic experience.

The visual exploration of two-dimensional figurative representations activates universal vision abilities, which are culturally and historically implemented. By combining psychological and cognitive studies with historical and eye-tracking analysis, this interdisciplinary proposal explores how our visual experience of paintings simultaneously addresses three pictorial experience levels: material, mental and historical. To deepen our knowledge of the vision and interpretation of paintings, we will focus on an in-depth empirical case study: the restoration of the Isenheim Altarpiece (1512-1516), painted by Matthias Grünewald. This Germanic polyptych, a masterpiece of Western art, is studied in its natural environment of observation, the Unterlinden Museum. This approach will allow us to engage in a comparative study of how different observers view the same unvarnished painting before and after restoration and integrate the altarpiece's material aspects with the observers' visual perception.

This research, conducted at Centre de Recherche et de Restauration des Musées de France (C2RMF) and the Center for Research on Arts and Language (CRAL) of the École des Hautes Etudes en Sciences Sociales (EHESS), adds to the traditional scientific analyzes of the materiality of the works mentioned above the eye-tracking method but also the cognitive and social science method. We aim to contribute to integrating "hard" and "human" sciences in the service of innovation in art research and technology.

The central aspect of the restoration of the Isenheim Altarpiece retained in this proposal is the cleaning of the panels. One of the main questions posed by us is to determine how the colors, the shapes, and the details of the paintings, which

become more intense after the lightening of the brown-yellow varnish, which currently protects the pictorial layer, change the visual perception and the impressions of observers of the artwork. Another question concerns the potential effect of restoring the images, weathered over time by the oxidation of the varnish, which has become opaque and darkens the panels, on the "historical" imagination of the altarpiece. We also wonder about the impact of conservation-restoration choices on the aesthetic experience of the people who look at the artwork—for example, leaving visible signs of the effects of time and even the marks of accidents on the paintings.

This study will use two experimental methods that are potent instruments in interdisciplinary research. The first is the use of an eye-tracker. The eye-tracker is a versatile instrument that can be exploited at different levels of cognitive resolution. This study combines eye-tracking results with qualitative methodologies (self-comments, questionnaires). The second innovative experimental aspect of the presentation will be computer models to analyze visual perception. Such models are helpful in that objective measures can be compared to a person's subjective perception. We propose to use this type of model to study the perception of the ten panels of the Issenheim Altarpiece before and after the varnish cleaning. From this point of view, detecting AOI (Areas of Interest) in the images will contribute to understanding the visual exploration of the altarpiece panels and

SYMPOSIUM: EYE-TRACKING AND THE VISUAL ARTS

BENNETT LECTURE THEATRE 1 - 14:00- 14:20

The SmART Viewer: the Impact of Smartphone Use on the Art Viewing Experience

Zoya Dare and Raphael Rosenberg

University of Vienna, Austria
zoya.dare@gmail.com

Smartphones are quickly becoming the primary device that many use to access the internet and the billions of images it makes available. This new context provides an intimate experience of a series of small images in quick, and nearly endless, succession. As this becomes the default image viewing setting, how do our experience of visual information and habits of viewing change? The current study investigates whether art viewing behaviour changes in relation to average daily screen time. We present measurements of attention span and memory retention as well as a self-reported aesthetic experience. Results confirm some past research on the effects of smartphones on general cognition but also demonstrate some emerging viewing behaviours.

SYMPOSIUM: EYE-TRACKING AND THE VISUAL ARTS

BENNETT LECTURE THEATRE 1 - 14:20- 14:40

Two Ways of Seeing: Investigating the Perception of a Painting's Surface vs of its Subject in Light of Wollheim's Theory of Twofoldness

Anna Miscena and Raphael Rosenberg

University of Vienna, Austria
anna.miscena@univie.ac.at

According to aesthetic philosopher Richer Wollheim, when looking at painting our attention oscillates constantly between two elements: its subject matter and its surface qualities, a perceptual phenomenon which he calls "twofoldness". The existence of two-fold perception has been theorized in art historical writing at least since the nineteenth century, in parallel with the development of modern art. Nonetheless, unlike its art historical precedents which focus on aesthetic phenomenology, Wolheim´s theory considers twofoldness to be also observable from a behavioural perspective, as assertion which calls for an empirical investigation.

This paper reports the results of two eye-tracking investigations on twofoldness: one conducted in a laboratory setting, one in the museum. We hypothesised that twofoldness, as described by Wollheim, could find a physiological correspondence in a viewer´s eye-movements and that it could be described as an the alternation of local and global aspects in a scanpath. In order to test this hypothesis, we examined the behaviour of viewers looking at modern art. Egon Schiele´s paintings, in which surface qualities are evident also to non-experts and play an active role in shaping identifiable subjects, lend themselves perfectly to this type of investigation.

SYMPOSIUM: EYE MOVEMENTS DURING TEXT PROCESSING AND MULTILINE READING: NEW CHALLENGES AND OPPORTUNITIES FOR INSIGHTS

BENNETT LECTURE THEATRE 2 - 10:00- 12:00

Chair: Julie Kirkby

Bournemouth University, United Kingdom
jkirkby@bournemouth.ac.uk

Symposium Organisers: The Reading Research Group, Bournemouth University.

For over a half century, the study of reading has been greatly enhanced through the recording and analysis of readers' eye movements. However, even though most reading situations involve texts with multiple lines, eye movement reading research has been dominated by single line reading studies. Investigation of text processing can provide insights into language comprehension and inferencing that more closely map the complexity of the everyday cognitive tasks. This symposium will present research that attempts to provide such insights and tackle the challenges of multiline reading experimentation.

SYMPOSIUM: EYE MOVEMENTS DURING TEXT PROCESSING AND MULTILINE READING: NEW CHALLENGES AND OPPORTUNITIES FOR INSIGHTS

BENNETT LECTURE THEATRE 2 - 10:00- 10:20

Return Sweeps During Multiline Reading: The Influence of Text Justification and Column Setting in Chinese (and English) Readers

Jeannie Judge, Mengsi Wang, Donna Gill, Xuejun Bai, Chuanli Zang and Simon P. Liversedge

University of Central Lancashire, United Kingdom
jjudge@uclan.ac.uk

Reading multiline texts in logographic and alphabetic writing systems requires return sweeps. We examined presentation format on return sweeps in native Chinese (Experiment 1) and English readers (Experiment 2) to determine how text alignment and column setting influence reading behaviour across two different written orthographies. Participants read left-aligned and fully justified multiline texts presented in one and two columns. We reasoned that more return sweeps and greater variability in line length might be disruptive to reading at a global (text reading time) and local (sweep targeting/correction) level. Chinese readers fixated further to the extremes of the final character of a line for text presented in two than one column, and for left-aligned than fully justified text. These effects indicate less reliance on line-final parafoveal processing for text in two columns, and for left-aligned text. Participants were more likely to undershoot line-initial target words following a long than a short return sweep due to greater saccadic targeting error for longer return sweeps. Similarly, undersweep fixation durations were longer for text in two columns than one column suggesting costs associated with more return sweeps. We will assess the results for English readers (currently under analysis) against those of the Chinese readers.

SYMPOSIUM: EYE MOVEMENTS DURING TEXT PROCESSING AND MULTILINE READING: NEW CHALLENGES AND OPPORTUNITIES FOR INSIGHTS

BENNETT LECTURE THEATRE 2 - 10:20- 10:40

The Eye-voice Span during Multiline Reading: The Implications of Return-sweeps

Victoria Adedeji, Julie Kirkby, Martin Vasilev and Timothy Slattery

Bournemouth University, United Kingdom
vadedeji@bournemouth.ac.uk

The relationship between the eye-voice span (EVS) and eye movement control has been documented recently in single line reading studies (Inhoff et al., 2011; Laubrock & Kliegl, 2015), yet no recent studies have been carried out with multiline stimuli. Therefore, this relationship was examined across line boundaries by comparing fixations adjacent to the return-sweep and intra-line fixations in a group of developing readers. The EVS had a greater impact on line-final and undersweep fixations compared to intra-line fixations. However, the EVS impacted accurate line-initial and intra-line fixations similarly. Our findings replicate previous work (Laubrock & Kliegl, 2015) of longer fixation durations in response to large EVSs. We extend this work by showing that this effect is greater for line-final and undersweep fixations in children. Additionally, we found that accurate line-initial fixations were much longer than intra-line fixations. Taken together, these suggest that return-sweep saccades may be more costly during oral reading and may contribute significantly to longer reading times in children who primarily read aloud.

SYMPOSIUM: EYE MOVEMENTS DURING TEXT PROCESSING AND MULTILINE READING: NEW CHALLENGES AND OPPORTUNITIES FOR INSIGHTS

BENNETT LECTURE THEATRE 2 - 10:40- 11:00

Algorithms for Assigning Fixations to Lines of Text in Multiline Passage Reading

Jon Carr, Valentina Pescuma and Davide Crepaldi

SISSA, Italy
jcarr@sissa.it

A common problem in eye tracking research is vertical drift – the progressive displacement of fixation registrations on the vertical axis that results from a loss of calibration over time. This is esspecially problematic for experiments that involve the reading of multiline passages, where it is critical that fixations on one line of text are not erroneously recorded on another. Correction is often performed manually, but this is time-consuming and error-prone. Various methods have previously been proposed for the automated correction of vertical drift in the context of reading, but these have largely been developed in isolation with little attempt to systematically evaluate them, meaning that drift correction and line assignment techniques have been moving forward blindly. We document the major algorithms and evaluate them using both simulated and natural eye tracking data. Our results indicate that different algorithms are better suited to different types of drift phenomena, allowing us to offer evidence-based advice on algorithm selection.

SYMPOSIUM: EYE MOVEMENTS DURING TEXT PROCESSING AND MULTILINE READING: NEW CHALLENGES AND OPPORTUNITIES FOR INSIGHTS

BENNETT LECTURE THEATRE 2 - 11:00- 11:20

Analyzing Multi-line Reading Experiments: Automatized Pre-processing and Practical Recommendations

Sascha Schroeder and Domink Glandorf

Universität Göttingen, Germany
sascha.schroeder@psych.uni-goettingen.de

In this talk, we will explore the consequences of assigning fixations to lines automatically when analysing data from multi-line experiments. Using data from the MECO corpus we computed correlations for various eye-movement measures based on manually annotated data and data automatically pre-processed using different line-assignment algorithms. Correspondence for first-fixation duration, gaze duration, and total reading time was generally high (correlations varying r = .81-.96 between the different algorithms). By contrast, correlations for skipping probability (r = .53-.85) and go-past time (r = .38-.72) were substantially lower and should thus be interpreted with caution. For all measures, there were consistent differences between different assignment algorithms. In a next step, we investigated whether the effects of word length, word frequency, and predictability differed between manually annotated data and the different line-assignment algorithms. Results show that effect sizes obtained using automatically processed data are qualitatively and quantitatively similar to those obtained using manually annotated data with little differences between algorithms. We discuss our findings with regard to different strategies how to (semi-)automatically pre-process data from multi-line reading experiments. In addition, we will elaborate on further methodological challenges involved in such experiments and provide practical recommendations for their analysis.

SYMPOSIUM: EYE MOVEMENTS DURING TEXT PROCESSING AND MULTILINE READING: NEW CHALLENGES AND OPPORTUNITIES FOR INSIGHTS

BENNETT LECTURE THEATRE 2 - 11:20- 11:40

Scanpath Regularity as a Predictor of Performance on Reading Comprehension Assessments

Diane Mézière, Lili Yu, Erik D. Reichle, Titus von der Malsburg and Genevieve McArthur

University of Turku, Finland
diane.meziere@utu.fi

In recent years, researchers have explored the possibility of using eye movements to measure reading comprehension. Findings suggest that eye-movement measures can predict performance on reading comprehension assessments, but that the relationship between eye-movement behaviour and reading comprehension is mediated by differences in task demands between assessments. We compared word-based eye-movement measures to measures of scanpath regularity as predictors of reading comprehension scores. Scanpaths go beyond word-based eye-movement measures and capture the global pattern of fixations during reading. Scanpath regularity is relative to other participants' eye movements and factors in readers' adaptation to task demands. It may therefore be less sensitive to differences in task demands between assessments. We used a dataset in which participants were administered three reading comprehension assessments while their eye movements were monitored: the York Assessment for Reading Comprehension (YARC), the Gray Oral Reading Test (GORT), and the sentence comprehension subset of the Wide Range Achievement Test (WRAT). Results showed that word-based and scanpath measures both made unique contributions as predictors of reading comprehension scores, suggesting that scanpaths capture effects that are not caught by word-based measures and vice versa. Nevertheless, both types of measures were influenced by differences in task demands between reading comprehension assessments.

SYMPOSIUM: EYE MOVEMENTS DURING TEXT PROCESSING AND MULTILINE READING: NEW CHALLENGES AND OPPORTUNITIES FOR INSIGHTS

BENNETT LECTURE THEATRE 2 - 11:40- 12:00

Lower-level Oculomotor Deficits in Schizophrenia during Reading: Evidence from return-sweeps

Madison Laks, Andriana Christofalos, Stephanie Wolfer, Elisa C. Dias, Daniel C. Javitt and Heather Sheridan

State University of New York, Albany, NY, United States
mlaks@albany.edu

Return-sweep saccades are eye movements that are made when readers move from the end of one line in a passage to the beginning of the next line. Readers often under-shoot a return-sweep saccade and require a subsequent, corrective saccade to fixate at the appropriate location at the beginning of a line. Given that prior work examining oculomotor deficits in readers with Schizophrenia (Sz) has primarily focused on single-line and single-word reading tasks, return-sweep saccades have not yet been examined in Sz. To assess return-sweep saccades in Sz, we analyzed an existing dataset (Dias et al., 2021, Schizophrenia Bulletin) in which participants read multi-line passages in four different line-spacing conditions. The probability of making a corrective saccade following a return-sweep was significantly higher in readers with Sz compared to healthy controls. Because visual acuity constraints do not permit detailed lexical processing of line-initial words when return-sweep saccades are initiated, our findings provide evidence for low-level oculomotor deficits in Sz during reading. Additionally, the probability of making a corrective saccade was significantly higher for condensed-spaced passages compared to both the double- and triple- spaced passages, which is consistent with prior research showing that increased visual crowding disrupts saccade targeting.

READING

BENNETT LECTURE THEATRE 2 - 13:00- 13:20

Investigating the Time-course of Visuo-motor and Linguistic Processes during Reading using EEG Combined with Eye-tracking

Régis Mancini, Laure Spieser, Eric Castet, Boris Burle and Françoise Vitu

Aix-Marseille Université, France
regis.mancini@univ-amu.fr

Many models have been proposed to account for eye-movement behaviour during reading. Most share the assumption that readers' saccades are controlled in a top-down manner by the needs of ongoing word-identification processes. However, it remains undetermined whether eye fixations during reading (on average 225 ms) are long enough to enable predominant top-down control. Here we assessed the time course of visuo-motor and linguistic processes during reading using Fixation-Related Potentials (FRPs). Twenty participants read 316 French sentences, as well as 316 pseudo-sentences (all letters replaced by the letter "z"), while their eye movements and their electroencephalogram (EEG) were co-registered. FRPs were analysed using Unfold, an EEG-deconvolution and (non-)linear-modelling toolbox. We entered sentence type, frequency and length of the fixated word, and incoming-saccade launch site, as predictors. Our preliminary results showed an effect of sentence type on the N1 component (~150 ms) but mainly in near launch-site cases, thus when the (z-)word could be previewed during the previous fixation. In contrast, there was no effect of word frequency before 200 ms from fixation onset, thus suggesting that the effect of sentence type was visual rather than linguistic in nature. Therefore, word-identification processes might be too slow to efficiently control saccades during reading.

READING

BENNETT LECTURE THEATRE 2 - 13:20- 13:40

A Glimpse into the Neural Basis for Foveal and Parafoveal Processing: Combined Analyses of Eye Movements and Fixation-based fNIRS during Reading

Ralph Radach, Andre Rölke, Christian Vorstius and Markus Hofmann

Bergische Universität Wuppertal, Germany
radach@uni-wuppertal.de

We recorded eye movements while participants (N=36) read sentence pairs in German. The second sentence included a target word with high vs. low frequency, which was either of high or low predictability based on context provided in the first sentence. Using this methodology, we obtained a pattern of oculomotor data that is highly compatible with the existing literature, including parafoveal-on-foveal effects. Concurrently, we utilized the rapid sampling frequency of near-infrared spectroscopy (NIRS) to examine neural correlates of word frequency and predictability in the occipital and orbitofrontal cortex. The onset of fixations within target words served as trigger events for the recording of hemodynamic responses. We observed increased activation in the right ventral occipital cortex when the fixated target word N was of low frequency, attributable to increased processing load during saccade planning. Unpredictable low frequency words increased activity in the left dorsal occipital cortex upon the fixation of the preceding word N-1, presumably due to interference with top-down modulated expectation. There was no such interaction in the orbitofrontal cortex, implying that it is not involved in the resolution of predictions during natural reading. Implications of these data for theories of eye movement control in reading will be discussed.

READING

BENNETT LECTURE THEATRE 2 - 13:40- 14:00

Contribution of Oculometry and EEG Synchronization in the Understanding of the Origin of the Dyslexia: Evidence from a Phonological Lexical Decision Task in French Students

Aikaterini Premeti, Frédéric Isel and Maria Pia Bucci

MoDyCo Laboratory, UMR 7114, CNRS & Paris Nanterre University, France
kpremeti@gmail.com

Dyslexia is a learning disability and its etiology is still debated. Phonological deficit and visuo-attentional impairment are two of the most discussed hypotheses to explain the poor reading abilities reported in dyslexia population. In this study, both eye movements and event-related potentials (ERP) were simultaneously recorded in two groups of university students, with and without dyslexia, during a phonological lexical decision task. Five categories of stimuli implicating various levels of sublexical or lexical orthographic and phonological processing were used (i.e., French content words, pseudohomophones, pseudowords, consonant strings, and symbols). We examined the number and the duration of fixations, the amplitude of saccades as well as three reading related ERP components, namely the N170, N320 and N400. Preliminary data show more and longer fixations, and several saccades in the dyslexics compared to controls, as well as reduced ERP amplitudes in the three ERP markers of interest. Taken together, these findings lend support to our assumption that dyslexia is a multifactorial deficit implicating both visual and phonological impairments.

READING

BENNETT LECTURE THEATRE 2 - 14:00- 14:20

Distinct Patterns in Eye Movements and Fixation-related Potentials put Constraints on Models of Eye Movements in Reading

Elizabeth Schotter, Sara Milligan, Martín Antúnez and Horacio Barber

University of South Florida, United States
eschotter@usf.edu

Eye tracking studies reveal that readers make shorter fixations on foveated target words when previously perceived parafoveal preview words were higher frequency or linguistically similar to the target. This parafoveal preview benefit in eye movements does not reveal the extent to which parafoveal word processing, and the integration of that word with foveally obtained information, continues after saccade plans have been initiated. Therefore, we coregistered EEG and eye movements during a gaze-contingent display change paradigm and measured fixation related potentials (FRPs) upon foveal fixation. Eye movements showed primarily an effect of preview frequency, suggesting that saccade planning is based on the familiarity of the parafoveal input. FRPs, on the other hand, demonstrated a disruption in downstream processing (i.e., the N400 component) when parafoveal and foveal input differed, but only when the parafoveal word was high frequency. This suggests that lexical processing continues after the eyes have moved away from a word and that eye movements and FRPs provide distinct but complementary information about lexical processing in reading. These findings put constraints on models of reading by suggesting that lexical processes that occur before an eye movement program is initiated are qualitatively different from those that occur afterward.

READING

BENNETT LECTURE THEATRE 2 - 14:20- 14:40

The uUse of Sentential Constraint in Young and Older Adults: Evidence from Co-registered Eye Movements and Fixation-related Potentials

Federica Degno, Ascensión Pagán, Simon P. Liversedge, Richard Kirkden, Sarah J. White and Kevin B. Paterson

Bournemouth University, United Kingdom
fdegno@bournemouth.ac.uk

Eye movement and ERP studies report conflicting findings concerning age differences in the effects of sentential constraint on reading. Whereas eye movement studies report larger effects of sentential constraint by older relative to younger adults (see Zhang et al., 2022), ERP findings suggest that context effects are diminished or delayed for older readers (see Payne & Silcox, 2019). These contrary findings may reflect methodological differences, including the use of unnatural sentence displays in ERP research. To address these limitations, we used a co-registration technique to record eye movements (EMs) and fixation-related potentials (FRPs) simultaneously while young (18-30 years) and older (65+ years) adults read sentences naturally, and used sentential constraint to manipulate the predictability of specific target words. Eye movement analyses were conducted over all data (full EM dataset) and only data matching FRPs, while FRPs were analysed to capture both early (P1 and N1) and later (N400 and P600) components. Both EM datasets and early FRP components showed main effects of age-group and predictability, while the full EM dataset and later FRP components revealed larger predictability effects for older adults. We argue that this novel approach provides compelling evidence that older adults rely more on sentential constraint in reading.

References

Zhang, J., Warrington, K.L., Li, L., Pagán, A., Paterson, K.B., White, S.J. & McGowan, V.A. (2022). Are older adults more risky readers? Evidence from meta-analysis. Psychology and Aging, 37(2), 239-259. http://dx.doi.org/10.1037/pag0000522

Payne, B.R., & Silcox, J. (2019). Aging, context processing, and comprehension. Psychology of Learning and Motivation, 71, 215-264. https://doi.org/10.1016/bs.plm.2019.07.001

READING

BENNETT LECTURE THEATRE 2 - 14:40- 15:00

Are There Independent Effects of Constraint and Predictability on Eye Movements during Reading?

Roslyn Wong, Aaron Veldre and Sally Andrews

The University of Sydney, Australia
roslyn.wong@sydney.edu.au

Evidence of processing costs for unexpected words presented in place of a more expected completion remains elusive in the eye-movement literature. The current study investigated whether prediction error costs might depend on the source of constraint violation provided by the prior context. Participants' eye movements were recorded as they read predictable words or unpredictable alternatives that were semantically related or unrelated in three-sentence passages that were matched on cloze probability but differed in whether the source of constraint originated solely from the local context provided by the final sentence of the passage, from the global context provided by the first two semantically rich sentences of the passage; or from the combined local and global contexts. Linear mixed models assessed the relative impact of the type of prior constraint on readers' early and late processing of unexpected input. We interpret our results within a hierarchical generative framework of language comprehension and consider their implications for existing theoretical models of predictive processing.

PARAFOVEAL PROCESSING

BENNETT LECTURE THEATRE 2 - 16:30- 16:50

New Evidence on Parafoveal Syntactic Processing during Reading

Laura Schwalm and Ralph Radach

Bergische Universität Wuppertal, Germany
lschwalm@uni-wuppertal.de

Despite extensive research on extrafoveal linguistic processing during reading, there is a surprising gap regarding the role of syntax. It is still unclear whether syntactic information in the form of grammatical gender can be acquired in the parafovea and used to facilitate the processing of subsequent words. The issue is related to the question of whether small word groups like minimal noun phrases can be the functional target in the programing of reading saccades.

Two experiments used a gaze-contingent display change paradigm, manipulating syntactic fit within nominal phrases in German. Either the article (experiment 1) or the noun (experiment 2) was manipulated, resulting in a mismatch within the nominal phrase, depending on the condition ("den/die Fisch" vs. "die Fisch/Hecht"; the fish/pike). When the eyes crossed a boundary to the right of the preceding word, the mask was replaced to provide a syntactically fitting noun phrase. Results indicated a substantial elevation of viewing times on both parts of the noun phrase. Contrary to expectation, the effect was independent of noun frequency. This work provides direct evidence for parafoveal syntactic processing, suggesting that that grammatical gender is used to generate constraints on subsequent nouns. Consequences for eye movement control will be discussed.

PARAFOVEAL PROCESSING

BENNETT LECTURE THEATRE 2 - 16:50- 17:10

Transposed-Letter Allographic Effects in Arabic: Evidence from the Boundary Technique

Maryam A. Aljassmi, Sami Boudelaa, Kevin B. Paterson and Manuel Perea

Zayed University, University of Leicester, United Arab Emirates
maryam.aljassmi@zu.ac.ae

Research using the masked priming same-different matching task in Arabic strongly suggests that allographic variation (changes in a letter's shape as a function of its position in the word) can constrain lexical processing. Specifically, response times to target words (e.g., يسترون [ySTRw] "they cover") were shorter when preceded by a transposed-letter (TL) nonword prime in which transposed root letters maintained their shape (e.g., يتسرون [yTSRwn]) than when they did not (e.g., يسرتون [ySRTwn]). The present study investigated whether allography modulates TL priming in more naturalistic viewing conditions: when people move their eyes while reading. Accordingly, we used the boundary-change technique in which parafoveal preview of the target word was (a) identical (e.g., أخلقها –أخلقها 'I create her'), (b) a TL-nonword without allographic changes (TL-Allog: e.g., أخقلها -أخلقها), and (c) a TL-nonword with allographic changes (TL+Allog: e.g., ألخقها –أخلقها). Fixation times on targets were shorter only when the preview was identical. Thus, visual similarity alone does not modulate the integration of parafoveal and foveal information during sentence reading when the manipulated letters are root letters. These findings support the view that the order of the root letters in Semitic languages allows little or no perceptual noise in a normal reading scenario.

PARAFOVEAL PROCESSING

BENNETT LECTURE THEATRE 2 - 17:10- 17:30

Relating Foveal and Parafoveal Processing Efficiency with Word-level Eye-movement Measures of Text Reading

Timo Heikkilä and Jukka Hyönä

University of Turku, Finland
timo.t.heikkila@utu.fi

We examined the relationship between foveal and parafoveal word processing efficiency and word-level eye-movement parameters in reading among 74 adult readers (age range 17-66 years). Foveal processing efficiency was assessed with performance accuracy in the lexical decision and naming task, where 6-8–letter words and pseudowords were presented backward-masked for identification using different exposure times (20-60ms). Parafoveal processing efficiency was assessed by presenting 5-letter words and pseudowords for 150 ms to the right and left of the fixation point (1.33-2.24 degrees of visual angle). Mean overall performance measures of word naming and pseudoword naming were calculated for the naming tasks and a d' sensitivity index for the lexical decision tasks. Measures of foveal and parafoveal processing efficiency were used to predict eye-behaviour in reading 8 expository texts. Word-based LMM–analyses revealed that individual differences in foveal prossessing efficiency predict gaze durations especially in long and infrequent words. Foveal processing effiency also affected skipping probability, which was revealed as a main effect. Parafoveal processing efficiency predicts probabilities of skipping especially shorter words that are parafoveally previewed from shorter distances, while showing less relation to first-pass reading times. Even among adult readers word recognition skills predict word reading times and word skipping.

CLINICAL AND APPLIED I

BENNETT LECTURE THEATRE 8 - 13:00- 13:20

Eye Tracker Footage is It Enough? Retrospective Interview with Amateur Soccer Officials Using Eye Tracker Footage

Lee Waters, Itay Basvitch and Matthew Timmis

Anglia Ruskin University, United Kingdom
lee.waters86@hotmail.com

Objectives: Eye tracking is a valuable in assessing individuals gaze behaviour but does not unveil why they are engaging in certain practices. To address limitations in sport eye tracking research the present paper aims to investigate gaze behaviours of soccer officials during successful and unsuccessful offside decisions, but also why.

Methods: 20 male active amateur qualified (Level 4-7) soccer officials (Mage 22.5 SD 4.61 yrs) with an average experience of 41-50 games wore the SMI eye tracker during an applied attack versus defence drill. While reviewing the eye tracking footage, retrospective semi-structured interviews were conducted (M=20.4 min; SD=6.2; Range 11.7 – 26.8 min). Inductive thematic analysis was then performed.

Findings and Discussion: Key constructs of offside, decision making, obstacles and emotions were summarised as the higher order themes while making offside decisions. Gaze anchoring was highlighted to be a successful technique to allow officials to see all relevant information, whereas the type of offside was emphasised to be a key factor in correct interpretation.

Conclusions: Key constructs have been identified and explained, which can be shared with soccer officials through training regimes. Eye tracking technology has also been shown to be a useful and innovative reflective tool to assist in the understanding of individuals gaze behaviours.

CLINICAL AND APPLIED I

BENNETT LECTURE THEATRE 8 - 13:20- 13:40

Expertise Effects on Fixation Locations and Durations: Evidence from a Music-related Visual Search Task

Nicole Arco, Kinnera Maturi and Heather Sheridan

University at Albany, State University of New York, United States
narco@albany.edu

According to chunking and template theories of expertise, experts learn how to group together individual features into larger meaningful patterns called "chunks". Here, we tested if expert musicians use "chunks" to process music scores by re-analyzing an eye tracking dataset from a music expertise study by Maturi & Sheridan (2020). In Maturi & Sheridan (2020)'s study, participants completed a music-related visual search task while their eye movements were monitored. We extended Maturi & Sheridan (2020) by analyzing fixation locations and durations within bars of music. The experts allocated more attention to the beginning and end of a bar than non-musicians, as shown by a higher proportion of fixations and longer fixation durations compared to non-musicians. Similar to this pattern of results, skilled chess players are more likely than less skilled players to fixate near the edges of squares on a chessboard (de Groot and Gobet, 1996). One possible explanation for why experts fixate closer to the edge of a bar of music is that they are using parafoveal processing to encode larger patterns (or chunks) that extend across multiple bars. Our findings suggest that there are qualitative differences in how experts and non-musicians allocate their attention while processing music scores.

CLINICAL AND APPLIED I

BENNETT LECTURE THEATRE 8 - 13:40- 14:00

A Matter of Background: How and When Does the Virtual Background in an Instructional Video Impact Learning?

Andrienne Kerckhoffs, Leen Catrysse and Halszka Jarodzka

Open Universiteit, Netherlands
Andrienne.kerckhoffs@ou.nl

Together with the growing use of instructional videos in educational settings, comes an increased demand for concrete research-based principles concerning the design of such videos. This study explored whether a video’s background affects students visual information processing, and learning outcomes. In a between-group design, participants (N=47, age 14-17, 35 female) were assigned to one of three conditions: video with a neutral, authentic, or off-topic background. We measured prior-knowledge and working memory capacity (WMC), recorded eye tracking data during the learning process, and measured learning outcomes and learning experience. The eye tracking data revealed that a neutral background distracted the viewers the least, allowing them to pay better attention to relevant parts of the video. Also, learners self-reported that the off-topic background was distracting, however, the negative effect on the learning outcomes did not reach statistical significance. Furthermore, the WMC level had a significant impact on visual information processing and learning outcomes. We did not find a positive effect of the authentic background. This study suggests that backgrounds in learning material can distract visual processes and be experienced as such. Hence, we conclude that educators should preferably use a neutral background for their learning video’s, especially for learners with lower WMC.

CLINICAL AND APPLIED I

BENNETT LECTURE THEATRE 8 - 14:00- 14:20

Classification and Staging of Parkinson’s Disease using Video-Based Eye Tracking

Donald Brien, Heidi Riek, Rachel Yep, Jeff Huang, Brian Coe, Corson Areshenkoff, David Breen, David Grimes, Mandar Jog, Donna Kwan, Anthony Lang, Brian Levine, Connie Marras, Mario Masellis, Paula McLaughlin, Jb Orange, Alicia Peltsch, Angela Roberts, Ang

Centre for Neuroscience Studies, Queen's University, Canada
dcbrien.work@gmail.com

Background: Over 80% of those diagnosed with Parkinson’s Disease (PD) progress to PD with dementia (PDD) – with mild cognitive impairment an intermediary step (PD-MCI) - suggesting a complex spectrum of pathology concomitant with aging. Biomarkers sensitive and specific to all stages of disease are required if clinically relevant diagnostics are to be developed.

Objectives: We used video-based eye tracking and machine learning to develop a simple, non-invasive screening tool sensitive to PD and the stages of PDD.

Methods: From 121 PD (46 CN / 47 MCI / 20 Dementia / 8 Other) and 108 healthy controls we collected video-based eye tracking measures on an interleaved pro/anti-saccade task. Features of pupil, blink, and saccade behaviour were used to train a machine-learning classifier to predict confidence scores for PD/PD-MCI/PDD diagnosis.

Results: The overall Receiver Operator Characteristic Area Under the Curve (ROC-AUC) of the classifier was 0.87, whereas the subtypes showed progressively increased AUC with progressing disease, with the AUC of PDD alone being 0.94. The classifier had a sensitivity of 83% and a specificity of 78%. Moreover, the confidence scores predicted PD motor and cognitive scores at baseline and longitudinally.

Conclusion: We operationalized measures of saccade, pupil, and blink from video-based eye tracking to achieve high sensitivity to the landscape of PD heterogeneity. This work provides a framework for true clinical utility, where a simple eye tracking task could be used to screen for PD/PDD and predict outcomes.

CLINICAL AND APPLIED I

BENNETT LECTURE THEATRE 8 - 14:20- 14:40

Gaze Patterns Reflect Expertise in Dynamic Echocardiographic Imaging

Jochen Laubrock, Alexander Krutz and Sebastian Spethmann

Brandenburg Medical School Theodor Fontane (MHB), University of Potsdam, Germany
laubrock@uni-potsdam.de

Domain experts and novices differ in the way they sample visual information. Information about experts' allocation of attention can be used for educational as well as for diagnostic purposes. However, eye tracking data on expertise in medical imaging is scarce, and in particular absent for echocardiography, he key imaging and diagnostic method in cardiology. How is expert diagnosticians' allocation of attention characterized? Here we used eye tracking on echocardiographic loops of patients with reduced ejection fraction and controls to compare 15 national or international board certified diagnostic experts with 15 beginning medical students. Participants viewed 21 one-second video loops of apical four-chamber views and were asked to assess the systolic function of the left ventricle (as a diagnostic task) and image quality (as a visual control task), while their eye movements were recorded at 1000Hz using an EyeLink Plus. Rating data show that experts had a higher sensitivity in discriminating between patient and control loops. AOI analyses show that experts fixated diagnostically relevant areas such as the left ventricle wall earlier and more often, but more importantly, novices spent a lot more time on visually salient, but diagnostically irrelevant regions for the current diagnostic task, like the mitral valve. It appears that experts have learnt to ignore the salient motion cue presented by the mitral valve, and to focus on diagnostically more relevant areas. In addition to enhancing the training of echocardiography, eye-tracking results might be useful to aid in characterizing echocardiographic expertise objectively.

CLINICAL AND APPLIED I

BENNETT LECTURE THEATRE 8 - 14:40- 15:00

Pilot Study of Ocular Microtremor in Healthy People and in Psychopathology

Irina Shoshina, Sergey Lyapunov, Yuliya Simon, Michail Ivanov, Viktoriya Stanovaya, Ivan Lyapunov and Alisa Kosikova

St. Petersburg State University, Russia
shoshinaii@mail.ru

Introduction. Ocular microtremor (OMT) is a natural oculomotor activity which originates in the oculomotor area of the brainstem. The amplitude of tremor oscillations is normally 20-40 arc seconds, the average oscillation frequency is 87 Hz. The working hypothesis - the parameters of eye tremor can be objective and adequate indicators of the mental state. Method. The parameters of OMT were recorded using an optical system elaborated by the authors and calculated using the author's program. Video recordings with the duration 0.3-1.5 were performed from the outer side of the sclera. Participants: 9 persons without psychiatric and neurological diagnoses, 7 patients with schizophrenia and 3 patients with delusional disorder. Results. Analysis of variance showed a significant difference between groups in certain ranges of frequency (ft) and amplitude (at) of tremor. Differences were observed between patients with schizophrenia and delusional disorder in ft71 (p=0.02), at67 (p=0.0009), at71 (p=0.00001), at110 (p=0.00006), mentally healthy and schizophrenic patients in ft71 (p=0.02) and ft110 (p=0.00001), healthy participants and patients with delusional disorder in at67 (p=0.001), at71 (p=0.001), and at110 (p=0.0001). Conclusion. OMT measurement could be a useful tool for clinical diagnosis of a number of conditions.

Supported by Saint Petersburg State University (ID 37590964)

POSTER SESSION I, 1 ATTENTION

STUDENT UNION SQUARE HALL - 15:00- 16:30

The Time Course of Inhibition of Return in an Extended Saccade Sequencing Paradigm

Christof Körner, Margit Höfler, Petra Schönfelder, Helene Halbmayr and Iain D. Gilchrist

University of Graz, Austria
christof.koerner@uni-graz.at

We tested the time course of saccadic inhibition of return and its relation with saccadic momentum. To this end, we extended a saccade sequencing paradigm, originally developed by Ludwig et al. (2009). Participants were required to make eight saccades in a display that consisted of 16 circles as possible fixation locations. The gaze of the participants was guided either by a saccade-contingent endogenous arrow cue at fixation pointing to the next fixation location (Experiment 1), or by an exogenous cue appearing at the next fixation location (Experiment 2). The direction of the subsequent saccade could deviate from the preceding saccade direction by four different angles. After the sixth fixation in the sequence, the gaze was either directed back to a location fixated up to five fixations earlier (fixation lag) or directed to a new location. We found longer saccade latencies for fixation lags of up to three fixations back compared to fixations of new locations for endogenous cues, indicating the presence of inhibition of return. However, forward saccades were consistently initiated faster than those with greater angular deviations, indicating saccadic momentum. Nonetheless, there seemed to be an effect of inhibition of return in addition to saccadic momentum.

POSTER SESSION I, 2 ATTENTION

STUDENT UNION SQUARE HALL - 15:00- 16:30

Role of Attention in a Dual Task of Localization and Saccadic Remapping

Anna Dreneva, Uliana Chernova, Maria Ermolova and Joseph MacInnes

Lomonosov Moscow State University, Faculty of Psychology, Russia
annadrenyova@mail.ru

Predictive remapping is considered to be a crucial mechanism of maintaining visual stability. In this study, we aimed to investigate the role of attention in predictive remapping while performing a dual task paradigm which included two conditions: with and without saccadic remapping. In the first task participants remembered the clock hand position either after a saccade to the clock face (saccade condition requiring remapping) or after the clock being displaced to the fixation point (fixation condition with no saccade). In the second task they reported the remembered location of a dot on the periphery appeared for 1 s. We hypothesized that performance in these two tasks would interfere in the saccade condition, but not in the fixation condition, due to the attentional demands. We revealed that for the clock task, answers in the saccadic condition tended to underestimate the actual clock hand position by approximately 37 ms while responses in the fixation condition were close to veridical. The results point at significant interaction between the two tasks with decreased predicted accuracy in the clock task for increased error in the localization task, but only for the saccadic condition. These results demonstrate the key role of attention in predictive remapping.

POSTER SESSION I, 3 ATTENTION

STUDENT UNION SQUARE HALL - 15:00- 16:30

A Time-course Analysis of Food Cue Processing

Jonas Potthoff and Anne Schienle

University of Graz, Austria
jonas.potthoff@uni-graz.at

Background: Previous eye-tracking research has demonstrated that high-calorie food cues capture visual attention, particularly in individuals with overweight and weight concerns. The present experiment investigated whether this attentional bias can be influenced by context variables (i.e., the presence of pleasant low-calorie cues), and changes over time. This was studied via time-course analysis.

Method: Ninety women (mean age = 25 years) were presented with 60 picture pairs (à 6 s) that either showed a combination of high-calorie food + low-calorie food, high-calorie food + non-food, or low-calorie food + non-food. The women were assigned to one of three groups (n = 30): overweight with weight concerns (OW+), normal-weight with weight concerns (NW+), and normal weight without weight concerns (NW-). The relative number of fixations on (high-calorie) food cues was examined across the course of the picture pair presentations for one-second intervals.

Results: High-calorie food was fixated more often than low-calorie food and non-food only during the first second of the picture pair presentation. Subsequently, all participants (independent of the group assignment) showed the tendency to gaze at low-calorie cues when these cues were combined with high-calorie cues.

Conclusion: This study revealed timing-related context effects on visual food cue reactivity. While the early attentional bias to high-calorie cues could not be changed by context, this was possible during the later processing stage. Future studies need to examine whether the observed context effect can influence food choices and eating behavior.

POSTER SESSION I, 4 ATTENTION

STUDENT UNION SQUARE HALL - 15:00- 16:30

Attentional Orienting to Angry Gazes in Young Children with Autism Spectrum Condition

Li Zhang, Guoli Yan and Valerie Benson

Yancheng Normal University, China
zzli1992@126.com

Atypical joint attention has been frequently reported in individuals with autism spectrum condition (ASC) but, empirical studies suggest intact gaze following in this population.

The current study investigated gaze following under an emotional gaze cueing paradigm (GCP) context in young children with and without ASC. Real faces were presented centrally, and the task was to look at a parafoveal or peripheral target which was displayed following a gaze change, and on some trials an emotion change, in the central faces. The gaze change was either congruent (cued) or incongruent (mis-cued) with the location of the target. In an angry gaze condition the centrally-presented face switched expression following a shift of the eyes to the left or right, whereas in the neutral face condition there was no expression change. Typical children exhibited an increased gaze cueing effect (faster to look to the lateral target correctly cued by gaze relative to the mis-cued condition) for angry faces compared to neutral faces. This effect was absent in the ASC group who also showed disengagement difficulties from the central faces regardless of the face type and gaze direction. The findings provide evidence for atypical joint attention in an emotional GCP context in ASC.

POSTER SESSION I, 5 COGNITION

STUDENT UNION SQUARE HALL - 15:00- 16:30

Attentional Engagement and Disengagement Differences for Circumscribed Interest Objects in Young Chinese Children with Autism Spectrum Condition: An Eye Movement Study

Li Zhou, Li Zhang, Yuening Xu, Fuyi Yang and Valerie Benson

East China Normal University, China
52204107001@stu.ecnu.edu.cn

The current study aimed to investigate attentional processing differences for circumscribed interest (CI) and non-CI objects in young Chinese children with autism spectrum condition (ASC) and typically developing (TD) controls. In Experiment 1, a visual preference task explored attentional allocation to cartoon CI and non-CI materials, between the two groups. We found that ASC children exhibited a preference for CI-related objects compared to non-CI objects, and this effect was absent in the TD children. Experiment 2 utilised the traditional gap-overlap paradigm (GOP) to investigate attentional disengagement from CI or non-CI items in both groups and showed a similar disengagement performance for all children. Experiment 3 adopted a modified GOP (MGOP) to further explore disengagement in the two groups and results suggested that children with ASC exhibited 'sticky attention' compared to their TD peers, and that endogenous disengagement was influenced further in the presence of CI-related objects in the ASC children. However, exogenous disengagement performance was preserved in the ASC group. The current results have implications for understanding how the nature of engagement and disengagement processes can contribute to differences in the development of core cognitive skills in young children with ASC.

POSTER SESSION I, 6 COGNITION

STUDENT UNION SQUARE HALL - 15:00- 16:30

Altered Pupil Dynamics Associated with Cognitive Impairment in the Progression of Parkinson’s Disease

Jeff Huang, Brian Coe, Matthew Smorenburg, Donald Brien, Derek Beaton, Brian Tan, Connie Marras, Jane Lawrence-Dewar, Stephen Strother, Donna Kwan, Paula McLaughlin, Anthony Lang, Sandra Black, Elizabeth Finger, Morris Freedman, Michael Strong, Richard H.

Centre for Neuroscience Studies, Queen's University, Canada
1jh19@queensu.ca

The progressive neurodegeneration in Parkinson’s disease (PD) leads to impairments in autonomic, motor, and cognitive functions. Pupillometry is an easy-to-measure method that is increasingly used in clinical investigations to assess these functions. In addition to global luminance and arousal, pupil size is also modulated by converging bottom-up sensory and top-down cognitive signals. Therefore, the disruptions in neural circuitry due to neurodegeneration should affect pupil control, and disease progression and level of cognitive dysfunction should produce impaired pupil responses. We examined pupil dynamics in patients diagnosed with early-stage PD during an interleaved pro-/anti-saccade task annually over 4 years. Patients were categorized as cognitive normal (CN), mild cognitive impairment (MCI), and dementia based on composite neuropsychological scores. Compared to controls, PD patients showed significant reduction in pupil dilation suggesting changes in the top-down preparation signals. Pupil measures differentiated between CN, MCI, and dementia PD patients, and showed changes with disease progression. Our findings demonstrated links between pupil dynamics and PD neurodegeneration, showing that pupil measurements in visuomotor tasks have the potential to provide relevant early behavioural biomarker for diagnosis and tracking disease progression.

POSTER SESSION I, 7 COGNITION

STUDENT UNION SQUARE HALL - 15:00- 16:30

Study protocol: Eye-tracking Parameters as Biomarkers of Presymptomatic Frontotemporal Dementia

Teuni Ten Brink, Lize Jiskoot, Sanne Böing, Stefan Van der Stigchel, Esther van den Berg, Harro Seelaar and Jackie Poos

Utrecht University, Netherlands
a.f.tenbrink@uu.nl

Frontotemporal dementia (FTD) is a clinical, pathological and genetically heterogeneous neurodegenerative disorder. Disease pathology in familial FTD already emerges years before symptom onset, which is associated with subtle cognitive changes. For upcoming medication trials, sensitive biomarkers to assess disease stage and track progression are crucial. Cognitive measures could serve as biomarkers, however, standard neuropsychological assessment lacks sensitivity. We hypothesize that eye movement features could serve as a sensitive biomarker for FTD. Our primary aim is to identify which eye movement features can be used as a biomarker for FTD. We will assess which (structured and spontaneous) eye movement features (1) dissociate best between patients with FTD, Alzheimer's disease, presymptomatic FTD mutation carriers, and healthy controls, and (2) are sensitive to detect longitudinal cognitive decline in presymptomatic C9orf72, GRN and MAPT mutation carriers. A range of eye-tracking tests that have been related to FTD and Alzheimer's disease in previous research will be administered, including pro-/anti-saccade, oculomotor capture, smooth pursuit, self-paced eye movements, Brixton spatial anticipation, visual search (Trail Making Test), and free viewing. In addition to features derived from structured eye-tracking tests, such as the anti-saccade test, spontaneous eye movements are evaluated. Frequentists and machine learning techniques will be used.

POSTER SESSION I, 8 COGNITION

STUDENT UNION SQUARE HALL - 15:00- 16:30

Eye-Tracker Procedure to Analyze Sex Differences and Strategy induction for Solving a Mental Rotation Task

Raúl Cabestrero, Isabel Orenes, Laura Cepero, Antonio Rodán, Laura Fernández-Méndez, Pedro Montoro, Julia Mayas, Antonio Prieto and Mª José Contreras

Universidad Nacional de Educación a Distancia (UNED), Spain
rcabestrero@psi.uned.es

When individuals perform Mental Rotation tasks, individual differences emerge in solution strategies that could be described by the response patterns used to cope with the task. The purpose of this study was to manipulate instructions encouraging solving a Mental Rotation Task (MRT) with a holistic versus a piecemeal strategy. It is expected that both strategies could be elicited and measured through eye movements tracked when participants perform the task. Sex differences in response patterns and performance were also analyzed. 140 MR trials were presented using the Eyelink Portable Duo 2000 Hz. Participants completed two phases during the experiment. Each phase comprised 10 practice trials plus 60 experimental ones (30 same and 30 different). Additionally, five angular disparities were configured (0-45-90-135-180º). During the first phase, participants solved the task in their own style. During the second phase, half of the participants were encouraged to solve the task using the holistic strategy, while the other half were induced to apply a piecemeal strategy. Data analysis combined different eye movement parameters for indicating two distinct eye-patterns—fixating and switching patterns. Results were discussed highlighting the role that employed strategy plays when interpreting sex differences in Mental Rotation tasks.

POSTER SESSION I, 9 COGNITION

STUDENT UNION SQUARE HALL - 15:00- 16:30

Eye-tracking Measures of Aesthetic Experience

Maartje Raijmakers, Aleksandra Sobolewska and Eftychia Stamkou

Department of Psychology, University of Amsterdam, Netherlands
m.e.j.raijmakers@uva.nl

In aesthetics research for visual art, a central question concerns the relationship between the complexity of the artwork and the appreciation of the viewer. It has been hypothesized that this relation follows a Goldilocks principle, meaning that the highest appreciation is expected somewhere in between a very low and very high complexity of the scene. (Berlyne, 1971; Cutting, 2020). Much of this aesthetics research is based on self-reports of the viewer, mostly single questions about liking and appreciation. Recent studies show that the concept of aesthetic appreciation is multi-dimensional, including both affective and cognitive involvement (Hager et al., 2012; Schindler et al., 2017). In this study, we relate the multi-dimensional self-report measures of aesthetic experience to measures derived from eye-tracking (Nayak & Karmakar, 2019) while free viewing an artwork. To this end, we conduct an eye-tracking study of adults looking at selected artworks from the database of paintings rated for complexity (The Vienna Art Picture System, VAPS). The aim of this study is to test whether the Goldilocks principle as found with self-report measures, is also present with eye-tracking measures as a function of perceived and calculated visual complexity.

POSTER SESSION I, 10 COGNITION

STUDENT UNION SQUARE HALL - 15:00- 16:30

Does News Source Matter? Fake News Recognition and Message Credibility in Social Media: An Eye-tracker Approach

Elena Artemenko, Maksim Terpilovskii, Taisiia Ulianova, Victoria Vziatysheva and Olessia Koltsova

HSE University, Russia
edartemenko@hse.ru

Finding predictors of news misinformation detection is one of the most relevant tasks in text processing in social media research. In this pilot study, we use the eye-tracker approach to check whether the news source visibility and source type affects the accuracy of fake news recognition and news credibility; we adopt a 10% Type-I error threshold. Russian native-speaking participants (N=9) were offered 17 political news messages via a unique web interface thus producing 153 observations. Each news item contained a headline, the news source type, the date of publication, and the news text. Participants were asked to evaluate each news item's credibility on a six-point Likert scale ranging from "true" to "fake". Psychometric data of the participants were also collected (e.g.,CRT). Accuracy (correctness of news recognition understood as the hit rate) and news credibility (measured on a six-point scale) were calculated as dependent variables. The source visibility (was it noticed or unnoticed by the participant) and source type (from domestic - i.e. Russian - media or from foreign media) variables were used as independent factors. Predictably, no statistically significant main effects or interactions were found for the accuracy. Therefore, we have no evidence that the source type or its visibility to the participant affect the accuracy of news categorization. However, the visibility of the source and its type affect the participants' perception of message credibility. The analysis showed the main effect of the source visibility ($t = -2.070$, $p < .05$) and an interaction between source visibility and source type ($t = 1.941$, $p < .10$). This means that foreign sources are significantly less trusted than domestic sources, but this effect only exists when the participants have actually seen the source.

POSTER SESSION I, 11 COGNITION

STUDENT UNION SQUARE HALL - 15:00- 16:30

Effects of Pictures in Instructions for Use

Michael Meng

Merseburg University of Applied Sciences, Germany
michael.meng@hs-merseburg.de

Research on multimedia learning has shown that people learn better from words and pictures than from words alone (Multimedia Principle, Mayer, 2021). However, it is less clear which effects pictures have when they are added to step-by-step procedural instructions which users typically read not to learn, but to act on the information they receive. In order to test effects of pictures on how users work through procedural instructions and interleave reading and acting, we created a simple software tutorial describing eight sequences consisting of 2-5 tasks to perform with the GIMP image manipulation program. There were two versions of the tutorial, differing with respect to whether they included pictures or not. Eye movements were recorded while subjects (N=42) worked through a tutorial and executed the respective tasks. Results show that subjects solved tasks more accurately and faster when procedures contained pictures. Eye tracking data revealed that time to process the instructions and transitions between the tutorial and GIMP did not differ between conditions. However, pictures supported subjects in applying procedural information to the GIMP software and encouraged them to adopt an atomized instead of a delayed task execution strategy (Jannin et al., 2019) which we argue improved overall performance as well.

POSTER SESSION I, 12 COGNITION

STUDENT UNION SQUARE HALL - 15:00- 16:30

The Effect of Bilateral Eye Movements on Episodic Memory Retrieval: An Assessment of Ageing and Disease Effects

Trevor Crawford and Megan Polden

Lancaster University, United Kingdom
t.crawford@lancaster.ac.uk

ilateral eye movements are the process of shifting your gaze from left to right in quick succession. Bilateral eye movements can lead to enhancements on subsequent memory and recall tasks. Previous research has prominently assessed healthy younger adult populations and has failed to assess the enhancement effect in clinical populations. Bilateral eye movements could offer therapeutic benefits to older adults with age-related cognitive decline and individuals with cognitive impairment. Across two experiments we assessed the effect of bilateral eye movements on memory and recall task performance. Experiment 1 aimed to replicate the effect in younger and older adults and assess whether the enhancement effect was exclusive to bilateral eye movements or elicited via top-down control tasks such as anti-saccade tasks. Participants were presented with 36 words to recall in a subsequent task and randomly allocated to an eye movement condition: bilateral, anti-saccade or fixed. Experiment 2 employed an online study methodology assessing the effect in individuals diagnosed with mild cognitive impairment, Alzheimer's and Parkinson's. Both experiments failed to replicate the enhancement effect across all participant groups with no significant differences in the number of words recalled based on eye movement condition. The results do not provide support for the use of bilateral eye movements to provide therapeutic benefits in clinical populations. This study adds to the growing number of failures to replicate and indicates an unstable effect lacking robustness. Future research should aim to establish clear and precise boundary conditions in which the effect is present, robust, and replicable.

POSTER SESSION I, 13 READING

STUDENT UNION SQUARE HALL - 15:00- 16:30

Word Length, Frequency, and Predictability Effects in Eye-movements in L1 Reading: A Systematic Comparison of 12 Languages

Daniil Gnetov, Victor Kuperman and Sascha Schroeder

McMaster University, Canada
gnetovd@mcmaster.ca

Reading behaviour in all studied written languages shows robust effects of "lexical benchmark" predictors: longer, less frequent and less predictable words take more effort to process. Yet little comparative evidence exists about cross-linguistic variability of these effects. This study compares lexical benchmark effects on eye-movements during text reading in 12 alphabetic and abjad (Hebrew) languages.

We used eye-movement data from the Multilingual Eye-Movement COrpus (MECO L1), collected from 543 participants in 12 countries reading short expository texts in their L1. Word frequency and predictability (a computational measure of word's similarity to previous context) were computed using fastText vector models pre-trained on Wikipedia corpora in respective languages. Linear-mixed models demonstrated a very high degree of cross-linguistic similarity between benchmark effect sizes across eye-movement measures. Despite cross-linguistic differences in respective distributions, word frequency, length and word predictability explained a similar amount of variance in all languages. The effects were also similar in whether they preferentially affected early (word frequency), late (word length) or cumulative (word predictability) oculomotor measures. Moreover, many observed instances of cross-linguistic variability – both in word length and frequency distributions and in behavioural effects sizes – can be traced back to morphological characteristics and script type of the language in question.

POSTER SESSION I, 14 READING

STUDENT UNION SQUARE HALL - 15:00- 16:30

Investigating the Effect of Negation on the Reading of Health Statements

Anna M. Plunkett, Meyrem Tompson, Sarah Wu, Kevin B. Paterson, Sarah J. White and Victoria A. McGowan

University of Leicester, United Kingdom
amp61@student.le.ac.uk

Negators (e.g. “not”) often appear in health statements (e.g. “Do not take more than one dose”). Previous literature shows that sentences containing negators have longer sentence reading times, lower comprehension accuracy, and are less likely to be remembered accurately, relative to sentences without negators. Given their short length, negators are likely to have low fixation rates. However, no studies have examined whether skipping of negators may contribute to difficulties in processing, understanding, and remembering sentences containing negators. Furthermore, no studies have examined whether making negators visually more salient, to increase the likelihood of fixation, will reduce such difficulties. Accordingly, in this study, participants will read fictional health messages without negation, with salient negation (negator bold and underlined), or with standard negation (negator formatted identically to surrounding text). Eye movements for the negator and the surrounding region will be examined, as well as sentence reading times, and immediate and delayed comprehension accuracy. The findings will reveal whether eye movement behaviour for negators is linked with difficulties in processing, understanding, and remembering health messages containing negators, and whether this is mitigated by increasing negator salience. Such findings will have important practical implications for the wording and visual presentation of health messages.

POSTER SESSION I, 15 READING

STUDENT UNION SQUARE HALL - 15:00- 16:30

Effect of Prior Knowledge on Re-reading Behavior After an Interruption and Text Comprehension

Guillaume Chevet and Véronique Drai-Zerbib

University of Burgundy, France
guillaume.chevet@hotmail.fr

Previous research showed that in case of interruption during reading participants re-read the part of the paragraph preceding the interruption when they are allowed to do so (Cane et al., 2012; Chevet et al., 2021) This behavior could prevent text comprehension from being degraded. We believe this is all the more true when the participant has no prior knowledge on the thematic of the interrupted text. 40 participants either with a Bachelor of psychology or physics took part in the experiment. They had to read 4 texts, 2 texts related to psychology and 2 texts related to physics. For each thematic, one of the texts was interrupted by a short distracting paragraph. Eye-movements were recorded during the full reading session. After each text, the comprehension was evaluated by means of questions of surface, targeting a proposition explicitly mentioned in the paragraph at the location of the interruption, and also by means of text-based inferential questions, requiring the subject to make the link between information of the paragraph located before and after the interruptions. We are expecting that participants who re-read the less the part preceding the interruption will have their performances in comprehension degraded especially for the text for which they have no prior knowledge.

POSTER SESSION I, 16 READING

STUDENT UNION SQUARE HALL - 15:00- 16:30

The Impact of Inter-word Spacing on Inference Processing: Evidence from Eye Movements

Andriana Christofalos, Madison Laks, Nicole Arco and Heather Sheridan

State University of New York, Albany, NY, United States
achristofalos@albany.edu

Prior research has shown that removing inter-word spaces disrupts word recognition processes during reading in alphabetic languages. The present study examined whether this disruption to word recognition would negatively impact inferential processing. We examined eye movements while participants read two-sentence long passages that were either strongly constrained (e.g., "Amy Parked her car on a steep hill. She watched as her car slowly rolled down the street.") or weakly constrained towards an inference (e.g., "Amy parked her car in her driveway. She watched as her car slowly rolled down the street."). We also manipulated text segmentation by presenting the passages with either normal inter-word spacing, or with the spaces replaced by numbers to produce an unsegmented text condition (e.g., "Amy7parked3her2car8in5her9driveway"). Consistent with prior work, our preliminary findings showed significantly longer fixation durations, higher numbers of fixations, and shorter saccade amplitudes in the unsegmented compared to the normal spacing condition. Data collection is ongoing, and as a next step we plan to use eye tracking to test our hypothesis that removing spaces will be more disruptive to processing in the weakly constrained compared to the strongly constrained condition. Our future findings will inform how lower-level processing interacts with higher-level processing during reading.

POSTER SESSION I, 17 READING

STUDENT UNION SQUARE HALL - 15:00- 16:30

Unexpected Sounds Inhibit the Movement of the Eyes during Reading and Letter Scanning

Michael Lowman, Martin Vasilev, Julie Kirkby, Katherine Bills and Fabrice Parmentier

Bournemouth University, United Kingdom
mlowman@bournemouth.ac.uk

Efficient cognitive functioning requires the ability to ignore task-irrelevant stimuli. However, it is important to detect unexpected changes in the environment, which could signal danger. It is well-established that the brain continuously monitors the auditory environment and can detect subtle changes to a repeated sound. Recently, it has been shown that the movement of the eyes' during reading is inhibited by unexpected sounds, referred to as novelty distraction. The current experiment investigated if novelty distraction is independent of higher-level linguistic processing by comparing a reading to a letter scanning task. Seventy-two Bournemouth University students either read sentences or scanned letter strings of the sentences for the letter ‘o’ with three auditory conditions: silence, standard, and novel. The results showed that first fixation durations were slightly longer in the standard sound condition compared to silence. Additionally, novel sounds led to a significant increase in first fixation durations compared to standard sounds (novelty distraction). Critically, the novelty distraction effect was identical between the two tasks, suggesting that it is independent of the type of task participants were doing. Because the letter scanning task eliminated higher-level language processing, the results suggest that novel sounds generally inhibit ongoing oculomotor processes independent of linguistic processing.

POSTER SESSION I, 18 READING

STUDENT UNION SQUARE HALL - 15:00- 16:30

Eye Tracking as a Tool for the Estimation of a Text Comprehension

Konstantin Chernozatonskiy, Victor Anisimov, Ksenia Babanova, Andrey Pikunov, Daria Zhigulskaya and Alexandra Latyshkova

Oken Technologies Inc., United States
kc@oken.tech

The growing interest to reader's comprehension estimation leads to searching for new methods enabling such estimation in real-time (or pseudo real-time). It can be used not only for more effective educational processes, but also in adaptation of text content for different purposes. In the study itself, Oken Reader application with eye-tracking tech-nology was used to assess perception and comprehension processes in reading. Twen-ty-three (23) respondents aged between 19 and 31 years (mean = 24.5, SE = 1.4, 65% women) took part in the study. As a result, fixation duration analysis on the base of proportion of correct answers to the item showed significant difference for mu, sigma, tau ex-Gaussian parameters and mean all between groups, where all answers were correct or false. For the medium groups demonstrated statistically significant difference only in tau parameter. Using the preliminary video supplement led to decreased num-ber of long fixations. The results of the study revealed the fundamental possibility of searching the correlations of physiological indicators such as eye movements with the process of comprehension the read text.

POSTER SESSION I, 19 READING

STUDENT UNION SQUARE HALL - 15:00- 16:30

How do We Read Multimodal Advertising Posters?

Anastasiia Konovalova and Petrova Tatiana

Saint Petersburg University, Russia
nastya111197@mail.ru

This study deals with the processing of verbal and non-verbal information in Russian multimodal advertising posters. The effect of lexical ambiguity in a slogan is checked out. 36 Russian participants (female = 28, mean age = 25) read 14 advertisements with lexically ambiguous slogans (the first meaning is supported by the picture and the second one – by the text) and 14 unambiguous advertisements. All the posters were edited and equalized by the size, font, background colour, the layout of the elements and the number of letters in the text. We used EyeLink 1000 Plus (SR Research) apparatus, monocular reading, head fixed.

The results show that total dwell time does not differ for ambiguous and unambiguous posters. Readers made almost the same number of fixations: Me = 32,39 for ambiguous posters and Me = 32,73 for unambiguous ones. Surprisingly, the first fixation was made on the verbal part of the poster significantly more often than on the picture (761 VS 247). Also, unambiguous slogans were read significantly slower (more fixations and more regressions) than ambiguous slogans. These findings contradict the previous results and assumptions that ambiguity in a text slows down the reading process, since the reader needs time to resolve it [Levelt, 1978; Sereno, O'Donnell, Rayner, 2006].

POSTER SESSION I, 20 READING

STUDENT UNION SQUARE HALL - 15:00- 16:30

Effects of Auditory Distraction during Reading: Evidence from the Eye Movements of Young and Older Adults

Hayley Barton, Kevin B. Paterson and Victoria A. McGowan

University of Northampton, United Kingdom
hayley.barton@northampton.ac.uk

Eye movement studies show that auditory distraction from irrelevant but meaningful background speech can disrupt reading by increasing regression rates and overall reading times. Little research has examined how this effect changes with age, however, although older adults might experience greater disruption due to poorer attentional control in older age. Accordingly, we recorded the eye movements of young (18-30 years) and older (65+ years) adults who read sentences in counterbalanced blocks of silence, irrelevant but meaningful background speech, and meaningless background speech. In addition, to test the hypothesis that auditory distraction might disrupt reading by interfering with the semantic integration of words, participants were presented with sentences containing either a highly predictable or a less predictable word. Consistent with previous research, the meaningful background speech was most disruptive. However, while typical patterns of adult age differences in eye movement behaviour were observed, the older adults were not more affected by the presence of background speech. Finally, while the meaningful background speech produced increased effects of word predictability, consistent with this interfering with semantic integration, this effect did not differ across the two age groups. We consider these effects in relation to accounts of auditory distraction on eye movements in reading.

POSTER SESSION I, 21 READING

STUDENT UNION SQUARE HALL - 15:00- 16:30

Eye Movements during the Verification of Arithmetic Calculations

Christine Green, Zhichao Zhang, Asha Sajjid, Jonathan Watson, Chuanli Zang, Simon P. Liversedge and Reyhan Furman

University of Central Lancashire, United Kingdom
cgreen11@uclan.ac.uk

Basic written addition and subtraction are the building blocks for more complex calculations but little is known about the cognitive processes involved. The current study examined eye movements during the processing of correct and incorrect arithmetic computations. Participants (n=24) were presented with three-digit addition and subtraction sums in a formal vertical presentation on a computer screen. They then had to make judgements as to whether the answer was correct or incorrect whilst their eye movements were recorded using an EyeLink 1000.

Initial results show that eye movements are concentrated on the digits forming the second line, the central digits, for both addition and subtraction for correct sums. However, distinctly different eye movements were found for the error conditions, as the fixation count was greater on the central digit of the column that contained the error. Overall, the operator received fewer fixations than the digits indicating that, as it is necessary for the successful completion of the sum, it was being processed parafoveally without direct fixation. These eye movement data indicate that individuals follow distinct step-by-step cognitive processes when solving sums. Implications of the current results for understanding the time course of mathematical processing will be discussed.

POSTER SESSION I, 22 READING

STUDENT UNION SQUARE HALL - 15:00- 16:30

A Large-scale Eye-movement Study of Reading in Russian Children

Anastasiya Lopukhina, Vladislava Staroverova, Nina Zdorova, Nina Ladinskaya, Anastasia Kaprielova, Sofya Goldina, Ksenia Bartseva, Olga Vedenina and Olga Dragoy

Center for Language and Brain, HSE University, Russia
nastya-merk@yandex.ru

This study presents a cross-sectional comparison of eye movements during silent reading in Russian schoolchildren. We aimed to establish (1) the development of basic eye-movement characteristics with age, (2) the influence of psycholinguistic word properties (length, frequency, predictability) on eye movements at different ages, and (3) the association between phonological processing and reading.

We tested 222 children from grades 1-6 (age range 6-13; 105 girls). All children's non-verbal IQ (Raven, 2004), oral reading fluency, and comprehension (Kornev, 1997) were within the normative range. The children read 30 sentences (6-9-word long) adapted from the Russian Sentence Corpus (Laurinavichyute et al., 2019) while their eyes were tracked with Eyelink PortableDuo or 1000+. They also completed a complex phonological task 'Changing the Sound in a Pseudoword' (Dorofeeva et al., 2020).

The results indicate that progress in reading manifests in eye-movement characteristics from grades 1 through 4 and then slows down, although it does not reach the adults' level in grade 6 (Laurinavichyute et al., 2019). The effects of word length, frequency, and predictability become smaller with age, with a significant reduction between grades 1-2 and between grades 3-4. Better phonological processing is associated with shorter fixation durations, especially in grades 1 and 2.

POSTER SESSION I, 23 READING

STUDENT UNION SQUARE HALL - 15:00- 16:30

Parafoveal Processing in Chinese Reading: Further Evidence for the Multi-Constituent Unit (MCU) Hypothesis

Chuanli Zang, Zijia Lu, Xuejun Bai, Guoli Yan and Simon P. Liversedge

University of Central Lancashire, United Kingdom
czang@uclan.ac.uk

Unlike English, Chinese is a character-based, unspaced language. An intriguing question concerns how lexical processing in Chinese is operationalized across potentially ambiguous, multiple adjacent character sequences. We were particularly interested in processing of frequently occurring Multi-Constituent Units (MCUs), that is, linguistic units comprised of more than a single word, that might be represented lexically. Using the boundary paradigm (Rayner, 1975), we conducted six experiments and manipulated the preview of each constituent of target strings, including three-character famous people's names (consisting of a one-character family name followed by a two-character given name, Experiment 1), place names (consisting of a two-character modifier and a one-character noun, Experiment 2), product names and modern phonetic borrowings (these structures are not transparent and thus constituents were defined and assessed comprehensively in Experiments 3a & b, and Experiments 4a & b). The results from all six experiments showed very consistent patterns with more pronounced preview effect from the second constituent when the first constituent was present in the parafovea, very strongly suggesting that these frequently occurring MCUs are lexicalized and processed parafoveally as single units during Chinese reading. The results provide compelling evidence in support of the MCU hypothesis.

POSTER SESSION I, 24 READING

STUDENT UNION SQUARE HALL - 15:00- 16:30

The Processing of Chinese Three-character Idioms with a “1+2” Modifier-noun Structure

Ying Fu, Simon P. Liversedge, Xuejun Bai, Guoli Yan and Chuanli Zang

Tianjin Normal University, China
yfu11@uclan.ac.uk

Previous evidence demonstrates that Chinese three-character idioms with a 2-character modifier and 1-character noun structure (2+1 MN) are processed as Multi-Constituent Units (MCUs, Zang et al., 2021). However, it is unclear whether idioms with a 1-character modifier and 2-character noun structure (1+2 MN) are processed as MCUs, as there are less constraints from the first constituent over the second compared with 2+1 idioms. In Experiment 1, we monitored participants’ eye movements when they read sentences with embedded idioms. Idioms were either presented normally (without segmentation highlighting), or highlighted with segmentations for whole MCUs, or constituent parts that did or did not coincide with the structure. Reading times were shortest for whole unit segmentations and longer for non-structural segmentations. Experiment 2, used the boundary paradigm (Rayner, 1975) to orthogonally manipulate preview of the modifier and noun of 1+2 idioms (identity or pseudocharacter). A greater preview of the noun occurred on the modifier when the modifier was an identity than pseudocharacter preview. Overall, these results suggest that Chinese idioms with a 1+2 MN structure are processed as MCUs, both foveally and parafoveally.

POSTER SESSION I, 25 READING

STUDENT UNION SQUARE HALL - 15:00- 16:30

What's Up, popEye? Updates to popEye – an R Package to Analyse Eye Movement Data from Reading Experiments

Sascha Schroeder

University of Goettingen, Germany
sascha.schroeder@psych.uni-goettingen.de

popEye is an R package to analyse eye movement data from reading experiments (Schroeder, ECEM 2019; freely available at https://github.com/sascha2schroeder/popEye). A unique feature of popEye is that it allows semi-automatic analysis of text-level experiments. To this end, popEye implements a wide selection of state-of-the-art line assignment algorithms and sentence-level measures. In addition, popEye has been designed to be used in cross-linguistic studies and thus supports a wide variety of languages including Hebrew, Korean, and Chinese.

In this poster presentation, I will present some of the new features that have been implemented in popEye since its first release, including new options for data pre-processing, line-assignment, and automated cleaning that make the analysis process more flexible and transparent. I will also provide links to new tutorials that demonstrate how popEye is used and walk user through an example analysis. The poster presentation is intended to be open and interactive and is supposed to give (potential) users the opportunity to ask questions about popEye and discuss new feature requests.

POSTER SESSION I, 26 READING

STUDENT UNION SQUARE HALL - 15:00- 16:30

Evolution of Eye Movements across Five Expertise Level during Sight Reading of Music

Joris Perra and Véronique Drai-Zerbib

Laboratoire d'Étude de l'Apprentissage et du Développement, France
joris.perra@gmail.com

Sight-reading of music is a task in which the musician must perform a score during the first reading or with very little preparation (Wolf, 1976). It is a widely studied task in the music reading literature as it generates issues that involve a high level of cognitive processing.

The challenge facing the musician during sight-reading is to perform despite a conflict between, on the one hand, the need for time to decode the visual information and prepare a motor response, and on the other hand, a limited time determined by the rhythmic constraints to perform the score.

Eye movements variables are known to discriminate individuals according to their ability and difficulty to process visual information. Therefore, studying musicians' eye movements during a sight-reading task enables to discriminate musicians according to their level of expertise and to evaluate their ability to cope with the complexity generated under the temporal constraint linked to the production.

In this experiment, 68 musicians from 5 conservatory levels had to sight-read 34 excerpts of different level of complexity while their eye movements were recorded. Musical score consisted of both classical tonal of Western music and contemporary repertoire and eye-movements data were recorded using the SR Research Eyelink 2000Hz system.

The main results show that the study of eye movements makes it possible to discriminate the level of expertise of musicians, even those whose conservatory level is close. Furthermore, this experiment shows that complexity is a significant factor in the evolution of eye movements and should be taken further into account to explain the differences in eye movements between performers.

POSTER SESSION I, 27 READING

STUDENT UNION SQUARE HALL - 15:00- 16:30

Reading Speed for Different Power Distributions of Progressive Power Lenses using Eye-tracking

Pablo Concepcion Grande, José Miguel Cleva, Eva Chamorro, Amelia Gonzalez, Paulina Dotor and José Antonio Gomez-Pedrero

IOT, Spain
pconcepcion@iot.es

Progressive power lenses (PPL) are the most popular solution for presbyopes, who cannot focus clearly at near-vision due to aging. PPLs have a continuous and smooth increase in spherical power from the upper to the bottom region. This geometrical change induces unwanted peripheral astigmatism, which negatively affects vision and therefore reading speed (RS). The goal of this study is to evaluate how RS is affected by 3 PPLs (PPL-D optimized for distance-vision, PPL-N optimized for near-vision, and PPL-B a balanced design). Pupil position was recorded using a wearable eye-tracker system (Tobii-Pro-glasses 3) on 21 PPL users while reading a text out-loud located at 5.25m and 0.37m. A randomized block design test was conducted to compare the effect of power distributions on RS. Statistical analysis was performed using Statgraphics Centurion XVI.II software. At distance-reading vision, PPL-D showed statistically significant higher RS (PPL-D: 160±30wpm, PPL-B: 150±30wpm, PPL-N: 150±30wpm, P=0.01) than PPL-B and PPL-N. At near-reading vision, PPL-N has statistically significantly higher RS (PPL-N: 170±30wpm, PPL-B: 150±40wpm, PPL-D: 140±30wpm, P<0.01) than PPL-B and PPL-D. In conclusion, the power distribution of PPLs significantly affects RS when doing vision-based tasks. To provide the best visual experience, PPL selection must be considered user needs.

POSTER SESSION I, 28 VISUO-MOTOR

STUDENT UNION SQUARE HALL - 15:00- 16:30

The Temporal Order Judgment Between Saccade and Visual Stimulation Just after Saccade

Junhui Kim and Takako Yoshida

Tokyo Institute of Technology, School of Engineering, Japan
kim.j.ba@m.titech.ac.jp

Ballistic eye movement, saccade, disrupts temporal order judgment, which has been well investigated within a single modality such as vision while not being well investigated between one’s saccade and visual stimuli onset. Here we investigated the discrimination threshold of temporal order judgment before and after the saccade to see if the judgment can be illusory reversed.

Subjects initiated saccade toward a target dot, while a central dot disappeared 200 ms before or after, or simultaneously with target dot onset(central dot offset condition). Two horizontal white bars at the bottom and top were displayed simultaneously, with onset asynchrony from 0 to 675 ms. Subjects had to answer whether the saccade or the bars onset occurred first.

Psychometric functions on the proportion of “saccade” response against the relative temporal distance between the saccade and bars onset showed that Sigmoid-like function, suggesting the correct judgment when the saccade initiated from -500 to 100 ms relative to the bars onset, irrelevant to the central dot offset condition. Interestingly, proportion of “saccade” response increased when saccade was initiated 300 ms after the bars onset, which might suggest postdiction-like effect. Our results might be different phenomenon from what has been shown in the single modality study.

POSTER SESSION I, 29 VISUO-MOTOR

STUDENT UNION SQUARE HALL - 15:00- 16:30

3D Object Viewpoint Discriminability Influences Target-selection for Saccades

Emma E.M. Stewart, Ilja Wagner and Roland W. Fleming

Justus-Liebig University Giessen, Germany
emma.e.m.stewart@gmail.com

Based on a 3D object's geometry, some object viewpoints are easier to discriminate from their neighboring viewpoints than others. Humans use saccades to sample information in the world, but it is unknown whether object properties such as viewpoint discriminability can influence saccade target selection. This study tested whether participants can infer the discriminability of an object viewpoint, and how they use this inference when choosing saccade targets in a subsequent match-to-sample task.

In a saccade target selection experiment, subjects fixated a central cross and were presented with two different viewpoints of a 3D-rendered object on opposite sides of fixation. One viewpoint was always more discriminable (easier to match) than the other. Participants were instructed to saccade to one of the two objects. In one condition, subjects matched the object-viewpoint they saccaded to, by rotating an onscreen object. In a second condition they were asked to match either the saccaded or non-saccaded object-viewpoint.

Results show that participants changed saccade target selection depending on the report condition, swapping whether they fixated the most or least discriminable viewpoint between conditions. This suggests that complex inferences about 3D objects may be used to inform saccade target selection and information sampling to guide subsequent behaviour.

POSTER SESSION I, 30 VISUO-MOTOR

STUDENT UNION SQUARE HALL - 15:00- 16:30

Eye Am In Control: Sense of Agency for Saccades

Julian Gutzeit, Lynn Huestegge, Jens Kürten and Lisa Weller

Julius-Maximilians-Universität Würzburg, Germany
julian.gutzeit@uni-wuerzburg.de

The sensation of having control over one's voluntary actions and their outcomes is referred to as sense of agency. We examined sense of agency for saccades with implicit and explicit measurements.

Participants fixated different stimuli on a screen while eye movements were recorded. After a short delay following stimulus fixation the color of the stimulus changed. Participants then reproduced the interval between saccade-landing and color-change. We made participants believe that they had control over the color change in one condition and no control over it in the other condition. We analyzed differences in interval reproductions between both conditions as a correlate of temporal binding, which might be linked to an implicit sense of agency. Further, we collected explicit agency ratings from participants.

We found no differences of temporal binding between the two conditions but higher agency ratings for conditions in which participants felt they caused the color change.

These findings indicate that temporal binding might better be explained as a phenomenon of multisensory integration in our study. However, oculomotor actions may generate an explicit sense of agency comparable to manual actions. This has several implications for the application of eye-tracking technology and the design of eye-tracking interfaces.

POSTER SESSION I, 31 VISUO-MOTOR

STUDENT UNION SQUARE HALL - 15:00- 16:30

Ocular Movements to Study the Influence of Defocus Induction on VA Measurement

Clara Benedi-Garcia, Pablo Concepcion Grande, José Miguel Cleva, Marta Alvarez and Eva Chamorro

Indizen Optical Technologies, Spain
cbenedi@iot.es

Ocular movements could help to understand better how visual performance is affected by defocus during Visual Acuity (VA) measurement. For that purpose, a clinical study was performed in 6 non-emmetropic subjects (age 34±8 years old). VA logMAR was measured at 2 distances (FV, 4m; NV, 37cm) under the induction of different amounts of defocus (±1, ±0.5, ±0.25, and 0D) on both eyes while an eye-tracker (Tobii Pro Glasses 3, Sweden) recorded pupil position. The following variables were analyzed using Statgraphics (Statgraphics Centurion XVII, v17.2.07): Reading Time (RT), Total Duration of Fixations (TDF), and Fixation Count (FC). At FV, positive defocuses present worse values on the variables, since they cannot be accommodated (p-value<0.05,RT&TD@1D). At NV, non-statistically significant differences were found on positive defocuses with 0D, since it helps to accommodation. The most demanding conditions (0.2VA and ±1D) were analyzed according to age. At FV, negative defocus requires extra accommodation, and the task is more difficult for elder subjects (R2=0.68&0.88 for RT&FC). At NV, positive defocus helps to read to elder people (R2=0.52 for FC). Eye-tracker is a useful tool to study the influence of defocus induction facing VA tasks. It was found a dependence on age in the most demanding conditions.

POSTER SESSION I, 32 VISUO-MOTOR

STUDENT UNION SQUARE HALL - 15:00- 16:30

Exploring Perceptual Decoupling during Voluntary and Reflexive Eye Behaviour

Ziva Korda, Sonja Annerer-Walcher, Christof Körner and Mathias Benedek

University of Graz, Austria
ziva.korda@uni-graz.at

Eye behaviour differs between internally (IDC) and externally direct cognition (EDC) and thus is indicative of an internal versus external attention focus. Recent work proposed perceptual decoupling (i.e., eye behaviour becoming less determined by the sensory environment) as one of the key mechanisms involved in the observed eye movement differences. To investigate the effects of perceptual decoupling on the voluntary and reflexive eye behaviour during EDC and IDC we used a dual task approach, combining an external eye movement task with internal visuospatial and arithmetic tasks. Internal tasks were further manipulated by three workload conditions. We expected the effects to be moderated by task type (more pronounced for visuospatial internal activity), workload (higher for higher internal demands) and follow a characteristic time course relative to internal operations. Furthermore, we hypothesised that perceptual decoupling will be more pronounced in voluntary eye movements but to some degree still observable in the reflexive eye responses. Data from 50 participants was acquired and currently are being analysed with linear mixed models. The findings will and shed light on relevant conditions of perceptual decoupling.

POSTER SESSION I, 33 VISUO-MOTOR

STUDENT UNION SQUARE HALL - 15:00- 16:30

Anticipating Choice Behaviour in Strategic Settings via Machine Learning Modeling of Scanpath Subsequences

Sean Byrne, Adam Peter Frederick Reynolds, Luca Polonio, Massimo Riccaboni, Carolina Biliotti and Falco Bargagli Stoffi

IMT School for Advanced Studies Lucca, Italy
sean.byrne@imtlucca.it

To date, eye movement research has extensively utilised game environments to study the link between cognition and gaze behaviour in strategic settings. We demonstrate a machine learning (ML) classification method that accurately identifies participants' cognitive strategy before they commit to an action while playing matrix games that are commonly found in the economic literature. By applying ML models trained on scanpaths derived from complete sequences of eye movement data, we are able to classify scanpath subsequences generated from a hold-out subset of the participants. Our proposed ML approach outperforms traditional methods of analysis applied to the same economic game environments. This research aims to illustrate how eye-tracking data combined with ML can bring about an information asymmetry in favour of those who collect and process the data. We highlight how eye-tracking technology may be used to produce undesirable outcomes for individuals operating in strategic settings in the not-too-distant future.

POSTER SESSION I, 34 VISUO-MOTOR

STUDENT UNION SQUARE HALL - 15:00- 16:30

Interaction of Dynamic Error Signals in Saccade Adaptation

Ilja Wagner and Alexander C. Schütz

Phillips University Marburg, Germany
ilja.wager@staff.uni-marburg.de

Saccade adaptation corrects motor errors to maintain saccade accuracy. A recent study demonstrated that saccade adaptation is not only driven by accuracy feedback shortly after saccade offset, but also by temporarily delayed feedback signals if they are task-relevant (Wagner et al., 2021). Here we studied how delayed feedback from relevant stimuli interacts with conflicting feedback signals from temporally proximate, but irrelevant stimuli.We instructed participants to make a saccade towards an unfilled circle. Inside the circle, two discrimination stimuli were shown successively at opposite locations. Participants were instructed to discriminate the location of a gap, either on the first (discriminate-first-condition) or the second stimulus (discriminate-second-condition).We observed saccade adaptation in the discriminate-first-condition, but not the discriminate-second-condition. A second experiment showed that this was modulated by saccade target properties: using a small square, located in-between discrimination stimuli, instead of the circle, we observed saccade adaptation to the first stimulus in the discriminate-second-condition. The large saccade target in the first experiment might allow for a larger presaccadic attentional focus and lead to weaker object correspondence with the postsaccadic discrimination stimuli. Both factors might contribute to a greater flexibility in which feedback signals are selected and which are suppressed for saccade adaptation.Acknowledgements: This project was funded by the SFB/TRR 135 and the International Research Training Group, IRTG 1901, “The Brain in Action”, from the German Research Foundation (DFG).

POSTER SESSION I, 35 VISUO-MOTOR

STUDENT UNION SQUARE HALL - 15:00- 16:30

Do Horizontal, Vertical and Oblique Stimulus Motion Evoke Comparable Nystagmus and After-nystagmus in Human Vision?

Kirsten Williams, Paul McGraw, Timothy Ledgeway and Denis Schluppeck

University of Nottingham, United Kingdom
Kirsten.Williams@nottingham.ac.uk

The visual system uses optokinesis to help stabilise the retinal image. Optokinetic nystagmus (OKN) displays a sawtooth time-course and results in after-nystagmus (OKAN) when all lights are extinguished during optokinetic stimulation. Here we compared OKN and OKAN in both cardinal and oblique directions for a range of stimulus velocities. We varied the instructions given to participants to elicit look OKN (actively following features) or stare OKN (passively viewing and keeping in focus) and examined their relationship with OKAN. Human participants observed a sinusoidal grating (0.5 c/deg at 10, 20 or 40 deg/s) drifting in one of eight directions, equally spaced in polar coordinates. Eye position was measured using the Eyelink 1000 eye tracker. At 10 deg/s, OKN gain was similar during both look and stare conditions (~1.0) and was largely independent of motion direction. At 20 deg/s, stare OKN gain was reduced relative to look OKN and both were attenuated for vertical motion. At 40 deg/s, gain was further reduced, and gain differences between look and stare OKN disappeared. OKAN was more reliably produced during stare conditions. Contrary to previous reports, OKAN was evident for vertical stimulation, but the direction was reversed with respect to stimulus motion.

POSTER SESSION I, 36 VISUO-MOTOR

STUDENT UNION SQUARE HALL - 15:00- 16:30

Seeing the Forrest Through the Trees: Oculomotor Metrics are Linked to Heart Rate

Alex J. Hoogerbrugge, Christoph Strauch, Zoril A. Olah, Edwin S. Dalmaijer, Tanja C. W. Nijboer and Stefan Van der Stigchel

Utrecht University, Netherlands
a.j.hoogerbrugge@uu.nl

Fluctuations in a person's arousal accompany mental states such as drowsiness, mental effort, or motivation, and have a profound effect on task performance. Here, we investigated the link between two central instances affected by arousal levels, heart rate and eye movements. In contrast to heart rate, eye movements can be inferred remotely and unobtrusively, and there is evidence that oculomotor metrics (i.e., fixations and saccades) are indicators for aspects of arousal going hand in hand with changes in mental effort, motivation, or task type. Gaze data and heart rate of 14 participants during film viewing (https://studyforrest.org; Hanke et al., 2016) were used in Random Forest models, the results of which show that specifically the movement aspect of oculomotor metrics (i.e., velocities and amplitudes) links to heart rate – more so than the amount or duration of those features. We discuss that eye movements are not only linked to heart rate, but they may both be similarly influenced by the common underlying arousal system. These findings provide new pathways for the remote measurement of arousal, and its link to psychophysiological features.

POSTER SESSION I, 37 VISUO-MOTOR

STUDENT UNION SQUARE HALL - 15:00- 16:30

Cyclovergence Movements in Presence of Vertical Shear Disparity across depth planes

Saad Almajed, Philip Duke and David Souto

University of Leicester, United Kingdom
srha2@leicester.ac.uk

Cyclovergence (rotations of the eyes about their visual axes in the opposite directions) normally occurs to correct torsional misalignments of the eyes produced during eye movements (e.g., gaze elevation). Previous studies have shown that cyclovergence can be elicited visually by vertical shear disparity (equal and opposite vertical shear between the two eyes' images). Vertical shear disparity provides a signal to control cyclovergence. However, studies have only used stimuli in the fixation plane, so it is not known whether vertical shear disparities are effective in surfaces at a different depth from fixation. Here we investigated whether cyclovergence is elicited by vertical shear disparities in surfaces at different depths from fixation. Stimuli were stereoscopic surfaces (approx. 70deg.) comprising of arrays of texture elements. The surfaces contained vertical shear disparities (+/-1.5deg.) and were presented at a range of depths (horizontal disparities, +/-40 arcmin) with respect to fixation. Cyclovergence was measured using a nonius technique. We also examined the perceived inclination in depth of the stimuli, since a frontal surface appears inclined when vertical shear disparities are introduced. We found that vertical shear disparities in surfaces away from fixation are effective in evoking cyclovergence and inducing apparent slant.

POSTER SESSION I, 38 VISUO-MOTOR

STUDENT UNION SQUARE HALL - 15:00- 16:30

The Effects of Personal Interest Level on Gaze Bias for Visual Preference Decisions

Anna Yoshida and Keiko Momose

Waseda University, Japan

This study investigated the effect of the personal interest level (four levels) of an object (stimulus image) on gaze bias. Pictures of clothes and accessories that can easily attract attention in daily life were used as visual stimuli, and eye movements during preference decision making were recorded. The gaze bias (the bias of gaze toward the selected stimulus) was calculated and compared for each interest. The results showed that reaction time was longest for interest level 4, and characteristics of the gaze bias for the same were different from those for the other interest levels (1-3). The higher reaction time may be due to the liking effect combined with the time taken for selective encoding. The gaze likelihood curve showed that the curve started earlier with an increase in the interest level, which may have been due to differences in the reaction time. In the single dwell trials (looking at each object at once only), the opposite gaze bias was observed in the first look for interest level 4. When the personal interest level of the target was particularly high, the eye movement was characterized differently from that of targets with low interest level.

POSTER SESSION I, 39 VISUO-MOTOR

STUDENT UNION SQUARE HALL - 15:00- 16:30

It's Hard Not to Look - But Possible: Using Eye Movements to Study Inhibitory Control Difficulties in Multiple-action Control

Jens Kürten, Tim Raettig, Julian Gutzeit and Lynn Huestegge

University of Wuerzburg, Germany
jens.kuerten@uni-wuerzburg.de

Saccades are often regarded as mere by-products of input-related cognitive processes, not as controlled actions that potentially interfere with other actions. However, robust performance costs associated with executing a saccade in conjunction with another concurrent action have repeatedly been demonstrated. These costs are typically smaller for saccades than for actions of other effectors given the high prepotency of the former and the resulting preferential execution. Here, we utilised these features of saccades (high inherent prepotency while still requiring active control) to study how inhibition of a prepotent action (while initiating another action) can prove more difficult than the concurrent execution of both actions, thereby yielding relative dual-action performance benefits. Participants responded to a single peripheral visual target with either a dual action consisting of a saccade and a manual button press, a single saccade, or a single button press. The latter response demand required saccade inhibition. The results revealed high rates of inhibition failures (false-positive saccade executions) in single manual trials. While similar effects were also demonstrated with other action modality pairings (e.g., manual-vocal), saccades can be considered ideal for eliciting such dual-action action benefits and thus for studying inhibitory control in general.

POSTER SESSION I, 40 VISUO-MOTOR

STUDENT UNION SQUARE HALL - 15:00- 16:30

Relating Asthenopic Symptoms to Optometric Measures and Parameters of Binocular Vision

Joëlle Joss and Stephanie Jainta

FHNW University of Applied Sciences and Arts Northwestern Switzerland, Switzerland
joelle.joss@fhnw.ch

Asthenopic symptoms are related to heterophoria and problems of binocular vision. In a recent paper, we showed that vergence drift and fixation durations are related to symptoms (CISS-questionnaire), but optometric measures such as heterophoria, vergence or accommodative facility, AC/A-ratio or NPC did not significantly add to the explained variance of asthenopia. We re-analysed our data, in which binocular eye movements were recorded (EyeLink II) for 64 participants, and linear regression analyses related all parameters of binocular coordination (objective heterophoria, vergence drift, saccade disconjugacy, fixation disparity and fixation duration), and the above-mentioned optometric tests to 4 symptoms factors (eye comfort, reading process, image quality and fatigue), which we identified by a factor analysis of the CISS-questionnaire. Objective heterophoria and fixation duration predicted 20% of the variance in symptoms concerning the reading process (factor 2). Furthermore, fixation duration seems to be slightly, but not significantly, related to symptoms addressing fatigue (factor 4), whereas optometric tests are not significantly related to any asthenopia factor. Overall, objective measures during reading relate to asthenopic symptoms, however the lack of asthenopic symptoms prediction by daily optometric parameters is still unexplained. Therefore, further research is needed to find out suited optometric parameters to infer asthenopic symptoms.

SYMPOSIUM: UNSTABLE FIXATION AND NYSTAGMUS WITH A FOCUS ON THE NEXT GENERATION OF RESEARCHERS

BENNETT LECTURE THEATRE 1 - 10:00- 00:00

Chair: Frank Proudlock

University of Leicester, United Kingdom
fap1@le.ac.uk

Symposium Organisers: Frank A. Proudlock (University of Leicester), Mervyn G. Thomas (University of Leicester), Jonathan T. Erichsen (Cardiff University)

This symposium aims to better understand the continuum of abnormal fixational eye movements, from unstable gaze in paediatric eye diseases up to more overt involuntary oscillations of the eyes in the form of nystagmus.

The session summarises the effects of unstable fixation and nystagmus on spatial and temporal aspects of functional vision. The symposium will also review the structural anomalies associated with disrupted foveal development, especially in relation to genetic causes of nystagmus and outline the impact of eye oscillations on clinical electrophysiological testing of underlying retinal abnormalities.

The session has been designed to provide an opportunity for an up-and-coming generation of researchers in the field of unstable fixation and nystagmus to present their work.

SYMPOSIUM: UNSTABLE FIXATION AND NYSTAGMUS WITH A FOCUS ON THE NEXT GENERATION OF RESEARCHERS

BENNETT LECTURE THEATRE 1 - 10:00- 10:20

Fixation Eye Movements in Pediatric Eye Diseases

Fatema Ghasia

Cleveland Clinic, United States
fatemaghasia@gmail.com

Dr. Fatema Ghasia is clinician-scientist with expertise in pediatric ophthalmology and binocular vision disorders and research interests in systems neuroscience, with emphasis on human and primate ocular motor control. She is Associate Professor and directs the Vision Neurosciences and Ocular Motility Laboratory at Cole Eye Institute, Cleveland Clinic. One of the main emphasis in the laboratory is studying the visual sensory and oculomotor effects of abnormal visual experience in early life that results in amblyopia and strabismus and investigating treatment effectiveness. We have shown that the fixation instability in amblyopia arises from nystagmus or alterations in physiologic fixation eye movement (FEMs). The systematic analysis of FEM traces has revealed several features that can be utilized to detect the presence and severity of amblyopia and angle and control of strabismus. We have found that FEM abnormalities correlate with reduced contrast sensitivities and depth perception, and inter-ocular suppression experienced by these patients. We have also found that assessing FEM characteristics can be a valuable tool to predict functional improvement post amblyopia and strabismus repair. She will share her clinical and scientific interests and her journey to date and highlight the importance of studying eye movements in a variety of childhood eye diseases.

SYMPOSIUM: UNSTABLE FIXATION AND NYSTAGMUS WITH A FOCUS ON THE NEXT GENERATION OF RESEARCHERS

BENNETT LECTURE THEATRE 1 - 10:20- 10:40

Accuracy and Precision of Fixation is Correlated with Gaze Angle

Onyeka Amiebenomo, Lee McIlreavy, Jonathan T. Erichsen and J Margaret Woodhouse

School of Optometry and Vision Sciences, Cardiff University, United Kingdom
AmiebenomoOM@cardiff.ac.uk

Purpose – In infantile nystagmus, the null zone tends to favour better oculomotor control even when it is eccentric. We investigated whether gaze position affects fixation in typical participants.

Methods – Nine emmetropes fixated vanishing optotype Landolt C targets at 4m, for 7 gaze angles (±45°; 15° apart) while performing a resolution threshold task. Eye movements were recorded at 1000Hz. Eye position accuracy and precision was derived from a bivariate probability density function. The isocontour surrounding the gaze positions with the highest 68% probability density was selected for further analysis. The length of the vector from the target position to the isocontour centre measured accuracy (perfect fixation is zero), and larger contour area reflected less precision.

Results – Mean eye position accuracy had a significant positive correlation with gaze angle [r(2) = 0.973, p = 0.027) as did precision [r(2) = 0.990, p = 0.010]. Mean contour shape (min/max diam.) had a significant negative correlation (less circular) with gaze angle [r(2) = -0.998, p = 0.002].

Conclusion – Fixation performance is progressively less accurate and precise with increasing eccentric gaze. Just as in people with nystagmus where nystagmus worsens outside the null zone, fixation appears to become more unstable as gaze shifts away from primary position.

SYMPOSIUM: UNSTABLE FIXATION AND NYSTAGMUS WITH A FOCUS ON THE NEXT GENERATION OF RESEARCHERS

BENNETT LECTURE THEATRE 1 - 10:40- 11:00

Investigating "Time to See" in Infantile Nystagmus

Katherine Ward, Fergal Ennis, Lee McIlreavy, Matt J. Dunn and Jonathan T. Erichsen

Cardiff University School of Optometry and Vision Sciences, United Kingdom
wardk6@cardiff.ac.uk

Infantile nystagmus (IN) is characterised by a continuous, involuntary oscillation of the eyes. Individuals with the condition may anecdotally report feeling slow to make visual discriminations, although the exact nature of this ‘time to see’ phenomenon has not yet been established. We hypothesise that the continuous oscillation (c. 3-4Hz) of the eyes in those with IN, which introduces an additional temporal component to their vision whereby their foveas are often moved off-target, is the cause for this increased ‘time to see’.

We are working to characterise ‘time to see’ in IN by using the novel approach of determining and comparing the presentation duration required by typical and IN participants to accurately resolve optotype targets. We will present preliminary results from our ongoing study of duration thresholds in IN, investigating the additional hypothesis that, within as well as across participants, longer duration thresholds will be associated with increasing nystagmus intensity (i.e. amplitude x frequency). Such a relationship could have potential applications in outcome measures for the efficacy of treatments which aim to reduce the intensity of nystagmus eye movements and their impact on visual perception.

SYMPOSIUM: UNSTABLE FIXATION AND NYSTAGMUS WITH A FOCUS ON THE NEXT GENERATION OF RESEARCHERS

BENNETT LECTURE THEATRE 1 - 11:00- 11:20

Phenotyping in Infantile Nystagmus

Helen Kuht and Mervyn G. Thomas

The University of Leicester, United Kingdom
hjk15@le.ac.uk

Infantile nystagmus is characterised by the involuntary rhythmic oscillation of the eyes. It often arises from mutations in genes expressed within the developing retina and brain. Genes implicated include those involved in melanin biosynthesis, solute carriers, transcriptional factors, G-proteins and ion channels. Afferent system defects are common and visualised using high resolution imaging techniques such as optical coherence tomography (OCT). Foveal hypoplasia represents arrested retinal development with varying grades of severity. The relationship between genotype and phenotype remains unclear.

In this talk, we will share the spectrum of eye movement disorders seen in infantile nystagmus, the techniques used to characterise foveal developmental defects and the relationship to genotype. We will utilise nystagmus twins to explore variability and penetrance of the nystagmus phenotype in shared genotypes. To understand the relationship between structure and function, we will correlate vision to the severity of the arrested foveal development and genotype.

SYMPOSIUM: UNSTABLE FIXATION AND NYSTAGMUS WITH A FOCUS ON THE NEXT GENERATION OF RESEARCHERS

BENNETT LECTURE THEATRE 1 - 11:20- 11:40

The Fovea is Horizontally Elongated in Infantile Nystagmus

Nikita Thomas, Jennifer H. Acton, Jonathan T. Erichsen, James Fergusson and Matt J. Dunn

Cardiff University, United Kingdom
nikitajadet@gmail.com

Infantile nystagmus (IN) develops in the first few months of life, prior to the appearance of the fovea. Despite the constant eye movements, visual perception in IN is usually stable. We hypothesised that the foveal pit would be horizontally elongated in adults with IN, corresponding to the streak of the retina over which visual attention constantly oscillates. Horizontal and vertically orientated foveal images were acquired with a long wavelength (λc 1040 nm) optical coherence tomography (OCT) system from 15 adults with idiopathic IN or IN associated with conditions not known to affect the fovea, and from 15 controls (age, sex, and ethnicity matched). Horizontal and vertical foveal pit diameter were calculated.

Foveal shape factor (vertical:horizontal pit diameter ratio) was significantly lower (more horizontal) in participants with IN, as compared to controls (0.88 vs. 0.96, $p = 0.02$, BF10 = 2.05). These results suggest that early-onset nystagmus may have a direct impact on foveal development, since IN typically develops before the appearance of the fovea. The findings have important implications for understanding the relationship between eye movements and visual development.

SYMPOSIUM: UNSTABLE FIXATION AND NYSTAGMUS WITH A FOCUS ON THE NEXT GENERATION OF RESEARCHERS

BENNETT LECTURE THEATRE 1 - 11:40- 12:00

Abnormal Electroretinography in Albinism and Idiopathic Infantile Nystagmus

Zhanhan Tu, Christopher Degg, Michael Bach, Rebecca McLean, Viral Sheth, Mervyn G. Thomas, Irene Gottlob and Frank Proudlock

University of Leicester Ulverscroft Eye Unit, United Kingdom
zhanhan.tu@le.ac.uk

Albinism and idiopathic infantile nystagmus (IIN) are two common forms of infantile nystagmus with involuntary oscillation of the eyes which are commonly associated with retinal diseases. Severe morphological abnormalities of the fovea, optic nerve head (ONH) and peripapillary retinal nerve fibre layer (ppRNFL) have been confirmed in albinism and IIN using optical coherence tomography (OCT). Full-field electroretinography (ffERG) can be used to diagnose abnormal retinal responses to photopic and/or scotopic light stimulation. In this talk, our primary aim was to determine whether ffERG responses are normal in albinism and IIN when measured in a large sample of adults using a robust methodology. A secondary aim was to investigate the effect of nystagmus on ffERG responses. Sixty-eight participants with albinism, 43 with IIN and 24 controls were recruited for comparing ffERG responses. Within-subject comparisons of ffERG responses when nystagmus was more or less intense were performed on 18 participants. Overall, our study found that individuals with IIN and albinism have abnormal ERG responses under photopic conditions. Nystagmus can negatively affect ERG recording and lower the ERG amplitude under the scotopic condition.

VISUAL SEARCH

BENNETT LECTURE THEATRE 1 - 13:20- 13:40

Age-related Changes in Oculomotor Indices of Top-down Selection during Visual Search

Fengjun Zhang, Hongyu Xie, Jingxin Wang and Doug Barrett

Academy of Psychology and Behavior, Tianjin Normal University, China., China
18863691911@163.com

Ageing has been associated with declines in the speed and accuracy of visual search. These have been attributed to reductions in processing speed, sensory acuity and top-down selection. The effect of these factors on oculomotor sampling, however remains poorly understood. The current study used one or two cues to manipulate the relevance of subsets of coloured stimuli during search in young (YA) and older adults (OA). Displays contained an equal number of red and blue Landholt stimuli. Targets were distinguished from distractors by a unique orientation and observers reported the direction of the gap on each trial. Single-target cues signalled the colour of the target with 100% validity. Dual-target cues indicated the target could be present in either coloured subgroup. The results revealed reliable group differences in the benefits associated with single- compared to dual-target cues. On single-target searches, OA made significantly more saccades than YA to stimuli in the uncued colour subset. Comparisons of z-transformed latencies also revealed a smaller reduction in the time between initial saccades and target fixations on single-compared to dual-target searches. These results support an age-related decline in the ability to restrict oculomotor sampling to a subset of relevant objects during visual search.

BENNETT LECTURE THEATRE 1 - 13:40- 14:00

Efficient Eye Movements during Search for an Object, Inefficient Eye Movements during Search for a Feature

Anna Nowakowska, Alasdair Clarke, Josephine Reuther and Amelia Hunt

University of Aberdeen, United Kingdom
a.nowakowska@abdn.ac.uk

Some models describe human search as optimal, others as a stochastic process. Across seven experiments, healthy observers searched through line segments, computer icons, mosaic patterns, polygons, and pens. In all experiments, the search array was split into easy and hard sides. The target was visible using peripheral vision on the easy side, but not the hard, such that only eye movements towards the hard side provided new information. An efficient strategy (that is, fixating the hard side) is no more or less difficult to implement across the stimulus sets. Nonetheless, strikingly different patterns of results emerged across different type of stimuli, which hinged largely on how the target of search was defined. Searching for an object oriented in a particular direction produced highly variable efficiency that, on average, did not differ from what would be expected from a stochastic strategy. Searching for a specified object produced far more uniform and efficient search behaviour. The results demonstrate that changes to seemingly irrelevant surface properties of the task can drastically alter measured strategy and performance. Moreover, searching for simple features is a useful and common laboratory task, but it may not always be representative of search for objects.

VISUAL SEARCH

BENNETT LECTURE THEATRE 1 - 14:00- 14:20

Categories of Eye Movement Errors and Their Relationship to Strategy and Performance

Letizia Caruso, Anna Nowakowska, Alasdair Clarke, Amy Irwin and Amelia R. Hunt

University of Aberdeen, United Kingdom
l.caruso.20@abdn.ac.uk

Visual search is a complex task involving perception, attention, decision, working memory, and strategy. Eye movements are a rich source of data that can reveal the complex interplay of these different components in determining the speed and accuracy of search performance. We analysed pre-existing data, focusing on how inefficient eye movements (defined as those directed to locations that could have been evaluated using peripheral vision) correlated with other identifiable eye movements errors such as revisiting previously fixated areas and “look-but-fail-to-see” (LBFTS) errors, in which participants fixated the target but then reported it absent. All three types of eye movement errors contributed to reaction time, and inefficient eye movements were independent of LBFTS errors. We also categorised scan-paths according to whether participants implemented obvious eye-movement “routines” (e.g. “reading” left to right, top to bottom), or appeared haphazard. There were large individual differences in the extent to which participants used routines. Routines were associated with slower search overall, and a higher prevalence of LBFTS errors. The relationships between strategies, identifiable eye movement errors, and search performance not only sheds light on the interplay of different components of search, but has implications for applied contexts involving information gathering and situational awareness.

VISUAL SEARCH

BENNETT LECTURE THEATRE 1 - 14:20- 14:40

Does Pre-crastination Explain Why Some Observers Make Sub-optimal Eye Movements in a Visual Search Task

Alasdair Clarke, Kyle Sauerberger, Anna Nowakowska, David Rosenbaum, Thomas Zentall and Amelia Hunt

University of Essex, United Kingdom
a.clarke@essex.ac.uk

A large proportion of variation in eye movements comes from individual differences. Many participants consistently move their eyes to locations that can be easily ascertained to neither contain the target, nor to provide any new information about the target’s location. Others engage in near-optimal search, executing eye movements to locations where central vision is most needed. In a pre-registered report, we test the hypothesis that inefficient search may represent a specific example of a larger tendency towards precrastination: starting sub-goals of a task before they are needed, and in so doing, spending longer doing the task than necessary. Participants perform two tasks: go and pick up two buckets and bring them back, and search for a line segment. Precrastination is defined as consistently picking up the closer bucket first, versus the more efficient strategy of picking up the farther bucket first. Search efficiency is the proportion of fixations directed to more cluttered regions of the search array. 146 participants have completed the experiment to date. We will reach the planned sample size of 200 by summer and will definitively address the hypothesis. Additional personality inventories will provide exploratory insights into individual differences in eye movement strategies.

VISUAL SEARCH

BENNETT LECTURE THEATRE 1 - 14:40- 15:00

Developing a Collaborative Framework for Naturalistic Visual Search

Charli Sherman, Anna Hughes and Alasdair Clarke

University of Essex, United Kingdom
c.s.sherman@essex.ac.uk

Interest in naturalistic visual search can be seen in a range of disciplines, tasks and settings, whether it is to determine where individuals look when driving, to find lost keys, or ways to maximise detection accuracy of tumours in x-rays. Yet much of this research has been deployed via screen based experiments, which may not directly assess the true behaviour carried out in these tasks. Our preliminary work aims to combine a series of visual search tasks to develop a 'naturalistic search task battery' which will allow us to understand search behaviour in more real-world contexts. Participants' behaviour and eye movements are recorded during the tasks. The tasks include a bookcase search where we explore top-down and bottom-up mechanisms by comparing target absent and present trials, and different levels of heterogeneity in the arrangement of distractors. We also investigate feature and conjunction searches and the effect of set size using Lego search tasks. Finally, look ahead fixations and strategy selection are investigated in a Lego building task and by making puzzles with or without a template. The aim is to form an open source, replicable and standardised set of tasks which can be used in a wide range of settings.

SYMPOSIUM: EYE MOVEMENTS AS A MEASURE OF HIGHER-LEVEL TEXT PROCESSING

BENNETT LECTURE THEATRE 1 - 16:30- 00:00

Chair: Mesian Tilmatine

Free University of Berlin & Radboud University, Germany
m.tilmatine@fu-berlin.de

Symposium Organisers: Jana Lüdtke (Freie Universität Berlin) & Mesian Tilmatine (Freie Universität Berlin)

Discourse as a core aspect of human cognition remains understudied in cognitive sciences (Mar, 2018). The use of eye-tracking technologies bears considerable potential for research on higher-level comprehension of written language (Cook & Wei, 2019). Consequently, there has been a trend in the past years to conduct more studies on higher-level processing of texts with a natural narrative flow, be that in the form of poetry, prose, or even newspaper articles. In this symposium, we will discuss our most recent contributions to this trend, mostly in the form of experimental data and improved or new models.

There are reasons why naturalistic texts are traditionally less prominent in eye-tracking research, mostly related to stimulus complexity. The symposium will thus review possible approaches to the methodological challenges associated with the use of naturalistic text stimuli. In that context, the talks will particularly focus on the role of individual differences in narrative and poetic perception (cf. Mak & Willems, 2019; Graf & Landwehr, 2015; Harash, 2021), as well as on possible ways to measure reading-related cognitive processes like mental simulation, mind-wandering, immersion, and foregrounding.

SYMPOSIUM: EYE MOVEMENTS AS A MEASURE OF HIGHER-LEVEL TEXT PROCESSING

BENNETT LECTURE THEATRE 1 - 16:30- 16:50

Mind-wandering during Reading of Siri Hustvedt’s Memories from the Future: Evidence from Eye-tracking

Johanna K. Kaakinen, Emilia Ranta and Jaana Simola

University of Turku, Finland
johkaa@utu.fi

Participants (N=52) read selected parts (135 paragraphs in total) of Siri Hustvedt's novel "Memories from the Future" while their eye movements were recorded. After 30 pre-selected paragraphs, participants responded to the 13-item mind-wandering scale (MWS, Turnbull et al., 2019) probing the focus and contents of current thoughts. A principal components analysis (PCA) of the MWS responses produced 4 components: 1) immersion, characterised by on-task focus, vivid and detailed imagery, and positive emotion; 2) off-task thoughts related to worrying about the future; 3) fluctuating thoughts about the past and others; and 4) voluntary verbalisations. In order to examine the associations between different types of mind-wandering episodes and eye movements, the PCA scores were used as predictors in linear mixed models for eye movement data. The results showed that readers' thoughts are reflected in their eye movements: high immersion and verbalisation increase the effects of word frequency and word length on eye fixation times. The results support the view that immersion to narrative text is an elementary part of reading experience, and that it is reflected in eye movement control during reading.

SYMPOSIUM: EYE MOVEMENTS AS A MEASURE OF HIGHER-LEVEL TEXT PROCESSING

BENNETT LECTURE THEATRE 1 - 16:50- 17:10

Reading Russian Poetry: An Expert–novice Study

Danil Fokin and Stefan Blohm

Uniwersity of Warsaw, Poland
d.fokin@uw.edu.pl

Studying the role of expertise in poetry reading, we hypothesized that poets' expert knowledge comprises genre-appropriate reading- and comprehension strategies that are reflected in distinct patterns of reading behavior.

We recorded eye movements while two groups of native speakers (n=10 each) read selected Russian poetry: an expert group of professional poets who read poetry daily, and a control group of novices who read poetry less than once a month. We conducted mixed-effects regression analyses to test for effects of group on first-fixation durations, first-pass gaze durations, and total reading times per word while controlling for lexical- and text variables.

First-fixation durations exclusively reflected lexical features, and total reading times reflected both lexical- and text variables; only first-pass gaze durations were additionally modulated by readers' level of expertise. Whereas gaze durations of novice readers became faster as they progressed through the poems, and differed between line-final words and non-final ones, poets retained a steady pace of first-pass reading throughout the poems and within verse lines. Additionally, poets' gaze durations were less sensitive to word length.

We conclude that readers' level of expertise modulates the way they read poetry. Our findings support theories of literary comprehension that assume distinct processing modes which emerge from prior experience with literary texts.

SYMPOSIUM: EYE MOVEMENTS AS A MEASURE OF HIGHER-LEVEL TEXT PROCESSING

BENNETT LECTURE THEATRE 1 - 17:10- 17:30

Unraveling the Social-cognitive Potential of Narratives using Eye-tracking

Lynn Eekhof, Kobie van Krieken, José Sanders and Roel Willems

Centre for Language Studies, Radboud University, Netherlands
lynn.eekhof@ru.nl

Narratives have a unique ability to disclose the inner worlds of others, leading various scholars to hypothesize about an intricate link between reading narratives and social-cognitive abilities such as perspective taking. One of the ways in which narratives can represent the inner worlds of characters is through the use of viewpoint markers, i.e., linguistic elements that provide readers access to the perceptions, thoughts, and emotions of characters. In this study (N = 90), we used eye tracking to study individual differences in the linguistic processing of these viewpoint markers. Having collected eye-tracking data from 90 participants who read a 5000-word non-fictional narrative, we found diverging reading patterns for perceptual viewpoint markers, that were processed relatively fast, and cognitive and emotional viewpoint markers, that were processed relatively slow. Moreover, perspective-taking abilities and self-reported perspective-taking traits facilitated processing both in general and for perceptual and cognitive viewpoint markers in particular. As such, our study extends findings that social cognition is of importance for narrative reading, showing that social-cognitive abilities are engaged specifically by the linguistic processing of narrative viewpoint. Moreover, these findings show that higher-level abilities such as social-cognitive abilities affect low-level reading processes.

SYMPOSIUM: EYE MOVEMENTS AS A MEASURE OF HIGHER-LEVEL TEXT PROCESSING

BENNETT LECTURE THEATRE 1 - 17:30- 17:50

Different Kinds of Simulation During Literary Reading: Insights from a Combined fMRI and Eye Tracking Study

Marloes Mak, Myrthe Faber and Roel Willems

Radboud University Nijmegen, Netherlands
marloes.mak@ru.nl

Mental simulation is an important aspect of narrative reading. In a previous study, we found that gaze durations are differentially impacted by different kinds of mental simulation. Motor simulation, perceptual simulation, and mentalizing as elicited by literary short stories influenced eye movements in distinguishable ways (Mak & Willems, 2019). In the current combined eye tracking and fMRI study, we investigated the existence of a common neural locus for these different kinds of simulation, using a fixation-related analysis for our fMRI data. We additionally investigated whether individual differences during reading, as indexed by eye movements, are reflected in domain-specific activations in the brain. We found a variety of brain areas activated by simulation-eliciting content, both modality-specific brain areas and a general simulation area. Individual variation in percent signal change in activated areas was related to measures of story appreciation as well as personal characteristics (i.e., transportability, perspective taking). Taken together, these findings suggest that mental simulation is supported by both domain-specific processes grounded in previous experiences, and by the neural mechanisms that underlie higher-order language processing (e.g., situation model building, event indexing, integration).

SYMPOSIUM: EYE MOVEMENTS AS A MEASURE OF HIGHER-LEVEL TEXT PROCESSING

BENNETT LECTURE THEATRE 1 - 17:50- 18:10

Effects of Centrality on Eye Movements: Predictions by Computational Language Models

Sascha Schroeder

University of Goettingen, Germany
sascha.schroeder@psych.uni-goettingen.de

Sentences differ in how much they contribute to the overall meaning of a text: central ideas are read more carefully and remembered better than more peripheral ideas. Centrality is usually operationalized using rating procedures in which participants explicitly evaluate the importance of a sentence in a text. In the present study, I used a computational measure of centrality based on a semantic vector space model. Centrality was defined as the cosine between the vector representing the overall meaning of a text and the vectors representing individual words or sentences. Eye movement data from the MECO corpus showed that centrality predicted participants' eye movements while controlling for word length and frequency. Centrality affected early reading variables in a complex manner: On the one hand, central words were more likely to be skipped during first-pass reading. On the other hand, however, they were also processed more deeply as indicated by longer first-pass reading times if fixated. I will discuss these findings in relation to other studies that have investigated centrality effects. In addition, I will compare the new centrality measure with other computational measures that have recently been used to predict eye movement behaviour.

SYMPOSIUM: EYE MOVEMENTS AS A MEASURE OF HIGHER-LEVEL TEXT PROCESSING

BENNETT LECTURE THEATRE 1 - 18:10- 18:30

Eye Movements as a Measure of Immersion and Foregrounding in Narrative Poetry Reading

Mesian Tilmatine

Free University of Berlin & Radboud University, Germany
m.tilmatine@fu-berlin.de

The cognitive process of foregrounding is considered to be crucial to the appreciation of literary language. However, it remains unclear how it relates to processes of narrative immersion, as these two processing routes are theorized to be separate mechanisms with different effects on the reader. For a direct comparison between textual elements evoking both processing routes, we conducted an eye-tracking study on narrative poetry (a story which is told in rhymes), namely on two excerpts of Goethe's "Faust". By comparing eye-gaze data and individual reading backgrounds with quantitative text data, subjective ratings, open statements, and tests of comprehension, we got more detailed insights into the exact workings of emotional activation for both routes. In the talk, we will present our methodology of integrating these rich data, as well as the most important results we found, and the implications for reader response theories and eye-tracking as a method in empirical aesthetics.

EYE MOVEMENT CONTROL IN READING I

BENNETT LECTURE THEATRE 2 - 10:00- 10:20

Understanding the Visual Constraints on Lexical Processing: New Empirical and Simulation Results

Aaron Veldre, Erik D. Reichle, Lili Yu and Sally Andrews

The University of Sydney, Australia
aaron.veldre@sydney.edu.au

Word identification is slower and less accurate outside central vision, but the precise relationship between retinal eccentricity and lexical processing is not well specified by models of either word identification or reading. In a seminal eye-movement study, Rayner and Morrison (1981) found that participants made remarkably accurate naming and lexical-decision responses to words displayed more than three degrees from the center of vision—even under conditions requiring fixed gaze. However, the validity of these findings is challenged by a range of methodological limitations. We report a series of gaze-contingent lexical-decision and naming experiments that replicate and extend Rayner and Morrison's study to provide a more accurate estimate of how visual constraints delimit lexical processing. Simulations were conducted using the E-Z Reader model (Reichle et al., 2012) to assess the implications for understanding eye-movement control during reading. Augmenting the model's assumptions about the impact of both eccentricity and visual crowding on the rate of lexical processing produced good fits to the observed data without impairing the model's ability to simulate benchmark eye-movement effects. The findings are discussed with a view towards the development of a complete model of reading.

EYE MOVEMENT CONTROL IN READING I

BENNETT LECTURE THEATRE 2 - 10:20- 10:40

Theorizing Dynamic Adjustment of Saccade Lengths in Reading and Dual-stage Progression of Visual Word Recognition

Jarkko Hautala

Niilo Mäki Institute, Finland
jarkko.hautala@nmi.fi

Prevalent theories of eye movement control in reading assume discrete selection of a saccade target word. Also prevalent dual-route models of visual word recognition assume a dichotomy: familiar words are recognized as wholes, whereas unfamiliar words are decoded by grapheme-phoneme conversion mechanism. However, these fundamental assumptions have been seldom subject of empirical testing.

Concerning the discrete saccade target selection process, analysis of landing position distributions in high skipping probability conditions found no evidence for bimodal landing position distribution, whose peaks would correspond to words n+1 or n+2. Instead, a dynamic adjustment model computing saccade length on the basis of n and n+1 length was sufficient to explain word length effect on landing position, refixation and skipping probabilities.

Concerning word recognition, analyses of eye movement measures during reading revealed that word frequency effect generally precedes word length effect, which was stronger for words of lower frequency. This pattern of results suggest that activation of orthographic word representations precedes decoding and that the decoding is faster for more activated words. Thus, the processing may be better characterized as dual stages than dual routes.

The implications of the findings for the architecture of computational models of visual word recognition and eye movement control in reading will be discussed.

EYE MOVEMENT CONTROL IN READING I

BENNETT LECTURE THEATRE 2 - 10:40- 11:00

Print Size as an Explanation for Inter-language Differences in Eye-movement Behavior during Reading: Empirical and Neurocomputational Evidence

Françoise Vitu, Hossein Adeli and Gregory J. Zelinsky

Laboratoire de Psychologie Cognitive, CNRS, Aix-Marseille Université, France
Francoise.Vitu-Thibault@univ-amu.fr

Readers' eye-movement behavior is very-much stereotyped. However, major differences have been reported between Western alphabetic languages and Eastern ideographic languages. In the framework of top-down models of eye-movement control during reading, these differences are commonly attributed to inter-word spacing. The assumption is that Chinese/Japanese scripts, which lack inter-word spaces, are read using less efficient word segmentation and/or different saccade-targeting strategies. Here we provide a much simpler explanation for inter-language differences, namely that they result from print size being two-to-four times greater in unspaced-language studies. Character size matters, but this tends to be ignored. Using a meta-analysis of dozens of studies and languages, we first found that benchmark eye-movement patterns, notably the Preferred Viewing Location (PVL) effect, only very mildly differ between languages when words are matched in angular extent and eye-movement behavior is measured in degrees of visual angle, rather than in letters as classically done. We then showed that MASC, our illiterate Model of Attention in the Superior Colliculus, predicts readers' eye-movement behavior in spaced and unspaced languages, simply using print size as a predictor. Our findings evidence universal visuo-motor principles of eye-movement guidance that generalize across languages and writing systems, while raising crucial theoretical and methodological issues.

EYE MOVEMENT CONTROL IN READING I

BENNETT LECTURE THEATRE 2 - 11:00- 11:20

Eye Movement Control during Reading and Skimming: Effects of Word Length

Shihui Wu, Kayleigh L. Warrington, Erik D. Reichle, Kevin B. Paterson and Sarah J. White

University of Leicester, United Kingdom
sw620@leicester.ac.uk

The effects of word length on word skipping and saccade targeting during reading for comprehension are well established. The present (OSF preregistered) study provides an experimental test of how reading goals (reading for comprehension vs. skimming for gist) modulate effects of word length. Critical short (3-4 letter) and long (8-9 letter) words were embedded into 96 sentence frames. The study had a within participants and within items 2(reading goal: read, skim) X 2(word length: short, long) design (64 participants). For both reading and skimming short words were significantly more likely to be skipped, less likely to be refixated, and had shorter gaze durations compared to long words. Crucially, there were significant interactive effects of reading goal and length for word skipping and initial landing position. Short words were especially likely to be skipped during first-pass when skimming compared to reading for comprehension. In addition, initial first-pass fixations landed further into long words during skimming compared to reading. Crucially, these results indicate that the visuo-oculomotor mechanisms underlying which words are fixated, and where words are first fixated, are modulated by readers' goals. The theoretical implications for the flexibility of the mechanisms underlying eye movement control during reading will be discussed.

EYE MOVEMENT CONTROL IN READING I

BENNETT LECTURE THEATRE 2 - 11:20- 11:40

A Cross-linguistic Study of Spatial Parameters of Eye-movement Control during Reading

Victor Kuperman

McMaster University, Canada
vickup@mcmaster.ca

Current theories of oculomotor control in reading differ in their accounts of saccadic targeting. Some argue that targets for saccades are solely selected on the basis of the rapidly changing sensory input, while others additionally allow for the reader's experiential biases to modulate saccade lengths. We investigated this debate using cross-linguistic data on text reading in 12 alphabetic languages from the MECO database. These languages vary widely in their word length distributions, suggesting that expected word lengths and corresponding biases towards optimal saccade lengths may also vary across readers of these languages. Regression analyses confirmed that readers of languages with longer words (e.g., Finnish) rather than shorter words (e.g., Hebrew) landed further into the word, even when sensory aspects relevant for saccade planning (e.g., word lengths) were controlled for. In the prevalent saccade type, one letter of a difference in mean word length between languages came with one-quarter letter of a difference in initial landing position and saccade length, and a decrease in 1.5% in refixation probability. Interpreted in the Bayesian framework, the findings highlight the relevance of global language-wide settings for accounts of spatial oculomotor control and lead to testable predictions for further cross-linguistic research.

EYE MOVEMENT CONTROL IN READING I

BENNETT LECTURE THEATRE 2 - 11:40- 12:00

Individual Differences and the Impact of Word Frequency on Eye Movements during Reading

Charlotte Lee, Hayward Godwin, Hazel Blythe and Denis Drieghe

University Of Southampton, United Kingdom
c.lee@soton.ac.uk

We examined the reading skill of 100 participants across a battery of individual differences tests and recorded their eye movements whilst they read neutral sentence frames containing either high or low-frequency words. Shorter fixation times and higher skipping rates were observed for high-frequency compared to low-frequency words. High scores in some tests (reading ability, print exposure and spelling) were associated with shorter gaze durations than low scores, and a subset of these tests (lexical knowledge and reading ability) were shown to influence the relationship between word frequency and gaze durations such that low-frequency words impacted fixation times and skipping rates less for participants scoring high on these tests. Next, common latent variables within tests were identified using a PCA. The factor lexical proficiency was associated with faster reading times, and a reduction in the impact of a low-frequency word, as was the case in total sentence reading times for a second latent variable indicative of overall processing speed. Our PCA also indicated that commonly used comprehension measures did not load on the same latent factor questioning whether they are indeed measuring the same reading skill.

READING DEVELOPMENT

BENNETT LECTURE THEATRE 2 - 13:00- 13:20

Seven Years Later – Executive Functioning Predicts the Development of the Perceptual Span during Reading

Johannes M Meixner and Jochen Laubrock

Brandenburg Medical School Theodor Fontane (MHB), Germany
johannes.meixner@mhb-fontane.de

The perceptual span indicates how much visual information can be processed within a single fixation. Here, we present data from two new waves of our longitudinal developmental study of the perceptual span during reading with more than 100 primary-school students, extending the data-collection period from first grade up to middle school. Overall, the size of the perceptual span did not significantly increase but rather stabilized during middle school – remaining at the level of sixth-graders. Matthew effects we reported earlier for early reading development resulted in stable and pronounced inter-individual differences at later grades for the high-level measures reading rate and perceptual span, whereas low-level oculomotor measures such as fixation duration and saccade length revealed compensatory patterns. Measures of executive functioning predicted reading performance seven years later: Children, who initially performed above average in both early reading and executive functioning, finally developed a much larger perceptual span and a higher reading rate compared to their initially below-average performing peers. This suggests that (in combination with linguistic skills) the efficient operation of executive functions such as shifting of attention, updating of working memory, and inhibition of pre-potent responses qualifies as a determinant of the development of the perceptual span.

READING DEVELOPMENT

BENNETT LECTURE THEATRE 2 - 13:20- 13:40

The Importance of the First Letter in Children's Parafoveal Pre-processing in English: Is it Phonologically or Orthographically Driven?

Sara Milledge, Simon P. Liversedge and Hazel Blythe

University of Central Lancashire, United Kingdom
smilledge@uclan.ac.uk

For both adult and child readers of English, the first letter of a word plays an important role in lexical identification. Using the boundary paradigm during silent sentence reading, we examined whether the first-letter bias in parafoveal pre-processing is phonologically or orthographically driven, and whether this differs between skilled adult and beginner child readers. Participants read sentences which contained either: a correctly spelled word in preview (identity; e.g., circus); a preview letter string which maintained the phonology, but manipulated the orthography of the first letter (P+ O- preview; e.g., sircus); or a preview letter string which manipulated both the phonology and the orthography of the first letter (P- O- preview; e.g., wircus). There was a cost associated with manipulating the first letter of the target words in preview, for both adults and children. Critically, during first-pass reading, both adult and child readers displayed similar reading times between P+ O- and P- O- previews. This shows that the first-letter bias is driven by orthographic encoding, and that the first letter's orthographic code in preview is crucial for efficient, early, processing of phonology.

READING DEVELOPMENT

BENNETT LECTURE THEATRE 2 - 13:40- 14:00

The Effect of Relevance in Children's Reading of Science Texts

Tuomo Häikiö, Oksana Kanerva, Johanna K. Kaakinen and Mirjamaija Mikkilä-Erdmann

University of Turku, Finland
tuilha@utu.fi

In the present study, 11-12-year-old Finnish children (N=34) read science texts designed for their reading level while their eye movements were registered as part of a larger project aimed at strengthening the scientific literacy of children. Prior to reading, the children were asked a question related to the topic of the texts, and instructed that they would need to write a short essay to answer the question. The texts contained both relevant and irrelevant parts with regard to the question. It was shown that during the first-pass reading, the probability of making a regression within a sentence was higher for the relevant sentences. This was mirrored in the first-pass rereading duration. Furthermore, the probability of making a regression out of the sentence was higher for relevant than irrelevant sentences. Faster reading manifested in shorter first-pass reading and rereading durations and higher probability of intra-sentence regression but not in differences in later rereading. The results indicate that 11-12-year-old readers are sensitive to relevant information. Furthermore, there were hints of strategic reading as the participants went back in text when they encountered relevant sentences. This is likely due to need to integrate the relevant information to the memory representation of the text.

READING DEVELOPMENT

BENNETT LECTURE THEATRE 2 - 14:00- 14:20

Children's Processing of Written Irony: An Eye-tracking Study

Henri Olkoniemi, Sohvi Halonen, Penny Pexman and Tuomo Häikiö

University of Oulu, Finland
hoolko@utu.fi

Ironic language is used frequently in communication. However, it is challenging for many to understand, e.g., for children. Comprehending irony is considered a major milestone in the development of children's social cognition, as it requires inferring intentions of the person who is being ironic. However, theories of irony comprehension turn a blind eye to developmental changes. The present study examined how children process and comprehended written irony in comparison to adults. Seventy participants took part in the study (35 10-year-old children, and 35 young adults). In the experiment, participants read ironic and literal sentences embedded in story contexts while their eye movements were recorded. They also responded to a text memory and an inference question after each story, and their levels of reading and empathy skills were measured. Results showed that comprehending ironic stories was harder for both age groups in comparison to literal stories, but the effect was larger for children. Readers took longer reading ironic than literal passages, and children already showed adult-like reading time patterns while resolving ironic meaning. Children's higher empathy skill was related to better irony comprehension and less rereading of ironic phrases. The results have implications for the current theories of figurative language comprehension.

READING DEVELOPMENT

BENNETT LECTURE THEATRE 2 - 14:20- 14:40

Concurrent and Predictive Validity of Reading Assessment by Eye-tracking and Machine-learning

Gustaf Ö. Seimyr, Elizabeth B. Meisinger, Hannah Manning and Mattias Nilsson

Lexplore, Sweden
gustaf@lexplore.com

We present a study on the concurrent and predictive validity of Lexplore - a fast, objective, and accurate method for reading assessment based on eye tracking and machine learning.

Lexplore was incorporated into the standard assessment battery in grade 1-6 at elementary schools in California (n = 1,484 students). The results from Lexplore in fall and spring was compared to i-Ready (a comprehensive benchmark widely used in US), oral reading fluency (ORF) scores, and the California end-of-year state tests (CAASPP) for grade 3-6. Concurrent and predictive validity was examined through correlational analyses, receiver operating characteristic (ROC) analyses, and classification accuracy statistics. The results show that Lexplore has a high concurrent validity as the results are very similar to the analysis of ORF ($r = .92$) and correlated well with i-Ready ($r = .75$). In both spring and fall, the correlation between Lexplore, ORF, and CAASPP ($r > .65$) was lower than i-Ready and CAASPP ($r > .80$), although both tests performed similarly when predicting readers below standard (ROC area under the curve > .79).

Given that Lexplore demonstrates comparable concurrent and predictive validity, we discuss what additional benefits eye tracking and machine learning can have for reading assessment in schools.

READING DEVELOPMENT

BENNETT LECTURE THEATRE 2 - 14:40- 15:00

The Eye-Voice Span in Children: Exploring Individual Differences

Julie Kirkby, Victoria Adedeji, Martin Vasilev and Timothy Slattery

Bournemouth University, United Kingdom
jkirkby@bournemouth.ac.uk

Oral reading is a complex task especially for developing readers, involving the concurrent recruitment of visual, oculomotor, lexical, semantic, memory, and articulatory processes (Godde et al., 2021; Kim et al., 2019). We measured these coordinative processes during oral reading using the eye-voice span (EVS), the distance between the eye and the voice while reading aloud (Adedeji et al., 2021; Buswell, 1921; Laubrock & Kliegl, 2015; Rayner, 1998). We obtained EVS data from 52 seven-to-ten-year-olds reading short passages, alongside offline ability measures of reading, spelling, vocabulary and RAN speed. We present reliabilities in individual differences in the EVS. Reading, vocabulary, and RAN, all predicted mean EVS, while spelling ability predicted the variability in the EVS. Spelling ability was also found to influence saccade length and reading ability influenced gaze duration. Neither vocabulary nor RAN speed predicted gaze duration or saccade length. Our pattern of results fits prior work (Parker & Slattery, 2021; Slattery & Yates, 2018; Veldre & Andrews, 2015, 2016) where spelling ability influences early letter encoding during reading, and fixation times are modulated by reading ability. We conclude that spatial EVS maybe more reflective of off-line measures related to reading ability than measures of gaze duration.

EYE MOVEMENT CONTROL IN READING II

BENNETT LECTURE THEATRE 2 - 16:30- 16:50

Word Difficulty Determines Regression Accuracy in Sentence Reading

Anne Friede, Christian Vorstius, Albrecht Inhoff and Ralph Radach

University of Wuppertal, Germany
afriede@uni-wuppertal.de

The occurrence of long-range regressive saccades during reading is related to linguistic processing difficulties. Two hypotheses regarding the accuracy of theses regressions appear viable. When reading difficult words, high processing demands may hinder the formation of a reliable memory trace for their location, leading to a lack of precision when regressions are executed. Alternatively, the location of such words may be specifically tagged in spatial memory as future saccade targets, enabling more effective regressions.

Participants were asked to read single-line sentences, identify a probe word that appeared to the right of each sentence, and to move the eyes back to the corresponding location in the sentence. Target words were either difficult or easy to read (low vs. high word frequency and orthographic regularity) and the target word location was either close to or far from the probe.

Difficult words substantially increased the precision of primary long-range regressions, being more often attained by accurate (single shot) regressions. If the target was missed, fewer additional saccades and less time were needed until the eyes fixated the target word. These results support the notion that orthographically and/or lexically difficult words assume a privileged status in visual-spatial memory as targets of future regressions.

EYE MOVEMENT CONTROL IN READING II

BENNETT LECTURE THEATRE 2 - 16:50- 17:10

Does Visual-similarity Cause more Regressions in Reading? An Eye-tracking Based Study

Mingqing Xie, Natalie Tze and Patrick Sturt

Durham University, United Kingdom
mingqing.xie@durham.ac.uk

Regressions account for 5-20% of eye movements in reading. Regressions are often found to accompany processing difficulty, which may be caused by syntactic ambiguity, semantic implausibility, or oculomotor targeting errors. In the present eye-tracking study, we examine regressions in sentences where processing difficulty arises due to implausibility, and we test the role of a context word's visual similarity relative to a plausible alternative. Sentences in the plausible condition (e.g., There was an old horse that John had ridden when he was a boy) provide a baseline to contrast with two implausible conditions. In the visually similar implausible condition, a lexical neighbour ("horse") appeared in the place of the plausible context word ("horse"), while in the visually distinct implausible condition a distinct word ("place") appeared, and each of these rendered the sentence implausible. A Bayesian analysis showed that, following the word ridden, participants made more regressions in the two implausible conditions than in the plausible condition, confirming the plausibility effect. However, during these regressive episodes, participants were no more likely to fixate the context word in the visually similar condition than in the visually distinct condition, with Bayes Factor evidence supporting a null effect of visual similarity, against our predictions.

EYE MOVEMENT CONTROL IN READING II

BENNETT LECTURE THEATRE 2 - 17:10- 17:30

When Functions Words Carry Content

Joao Vieira, Elisangela Teixeira, Erica Rodrigues and Denis Drieghe

University of Southampton, United Kingdom
joaomvieira@gmail.com

Eye movement studies on reading function words (FW) are rare, as are studies in Brazilian Portuguese (BP). While in most languages FW usually have less semantic information and are generally shorter, in BP they can carry gender and plurality marks and are often as long as content words (e.g. aquele/es/a/as, meaning “that one” or “those”). Though studies often report that FW are skipped more often than CW, a study in English from Schmauder, Morris and Poynor (2000) has shown that, with matched length and frequency, skipping rates and first fixation duration between CW and FW were similar. Here we report results from analyses on FW and CW using data from the RASTROS corpus of natural reading in BP (Vieira, 2020). We found that, in general, fixation duration decreased and skipping rates increased on shorter, more frequent and predictable words. On CW, predictability and length seem to affect processing independently. On FW, we found indications that only the longer FW were influenced by predictability. We argue that longer FW may be processed similarly to CW.

EYE MOVEMENT CONTROL IN READING II

BENNETT LECTURE THEATRE 2 - 17:30- 17:50

Does Omitting Mandatory Commas Affect the Reading Process?

Bernhard Angele, Ismael Gutiérrez Cordero, Manuel Perea and Ana Marcet

Bournemouth University, United Kingdom
bangele@bournemouth.ac.uk

Many orthographies mandate the use of commas to separate clauses and list items. However, casual writers routinely omit these mandatory commas; furthermore, commas are often misused (Trask, 2019). Even though the usage of commas (and their omission) is ubiquitous when reading texts, little research has been done on its effect on eye movements. One exception is the studies by Hirotani and colleagues (Hirotani, 2004; Hirotani et al., 2006), who found that using non-mandatory commas seemed to facilitate overall reading compared to omitting commas, although there were higher dwell times ahead of the commas. However, there was no evidence for longer global sentence reading times when mandatory commas were omitted. We present an eye-tracking experiment investigating the effect of omitting mandatory commas in five types of grammatical constructions in Spanish: concessive, adversative, listing, connective, and parenthetical. Sentences were presented with or without mandatory commas while readers' eye movements were recorded. We found no evidence for shorter global reading times due to comma presence. There was evidence for some differences in reading times (first-fixation duration, gaze duration, go-past time, total viewing time) for the pre-comma, post-comma, and subsequent regions, but there was no clear pattern suggesting a major advantage of comma presence.

EYE MOVEMENT CONTROL IN READING II

BENNETT LECTURE THEATRE 2 - 17:50- 18:10

TheroleofspacesinreadingFinnishtext

Raymond Bertram

University of Turku, Finland
rayber@utu.fi

In alphabetic languages, spaces are functional segmentation cues allowing for faster word recognition. Spaces delineate word boundaries and help position the eyes optimally in the word. It has been found that reading English text without spacing is 30-50% slower than reading with spaces (Rayner & Pollatsek, 1996; Rayner et al 1998). The current eye movement experiment compares reading Finnish sentences in spaced format and unspaced format. The main question was how specific text properties affect the detection of words and word boundaries in unspaced text and with that reading speed in general. The results showed that unspacing text in Finnish increases sentence reading times with about 35%, in line with what was reported earlier for English. More importantly, the results also indicated that unspaced sentence reading is affected by average word length and sentence length as well as by the average bigram frequency at word boundaries. The results thus indicate that even unexperienced readers of unspaced text are equipped to make use of specific word segmentation cues. Moreover, they indicate that there are some general text properties that facilitate unspaced text reading.

EYE MOVEMENT CONTROL IN READING II

BENNETT LECTURE THEATRE 2 - 18:10- 18:30

The Role of Visual Crowding in Eye Movements during Reading: Effects of Text Spacing

Tzu-Yao Chiu and Denis Drieghe

University of Southampton, United Kingdom
tyc1n21@soton.ac.uk

Visual crowding, generally defined as the deleterious influence of clutter on visual discrimination, is a form of inhibitory interaction between nearby objects. While the role of crowding in reading has been established in psychophysics research using RSVP paradigms, how crowding affects additional processes involved in natural reading, including parafoveal processing and saccade targeting, remains unclear. The current study investigates crowding effects on reading via two eye-tracking experiments. Experiment 1 is a sentence-reading experiment incorporating an eye-contingent boundary change in which letter spacing and preview validity are jointly manipulated. Experiment 2 is a passage-reading experiment with a line spacing manipulation. In addition to replicating previously observed letter spacing effects on global reading parameters, Experiment 1 found an interaction between preview validity and letter spacing indicating that benefits of reduced crowding on fixation duration were present only when parafoveal preview was intact. Experiment 2 found reliable but subtle influences of line spacing. Participants had shorter fixation durations, higher skipping probabilities, and less accurate return sweeps when line spacing was increased. These results extend the literature on how crowding affects reading and inform the question whether the observed benefits of reduced crowding reflect facilitated linguistic processing in the parafovea or improved oculomotor control.

DECISION-MAKING

BENNETT LECTURE THEATRE 8 - 10:00- 10:20

The Contribution of Visual Conduction Delay to Saccadic Reaction Time

Aline Bompas, Craig Hedge and Petroc Sumner

CUBRIC - School of Psychology, Cardiff University, United Kingdom
bompasae@cardiff.ac.uk

In fast sensory driven action decisions, reaction time is conceived as the sum of three components: sensory conduction, decision and motor execution. Over the last 10 years, we described a method to estimate the visual delay and decision time from reaction time distributions, that does not rely on modelling assumptions, but only on behavioural data and logic (Bompas, Campbell & Sumner, 2020 Psychological Review 127(4): 542-561). Here we gather 12 datasets from multiple labs to outline the defining properties of conduction delay in saccadic responses. We provide a first systematic description of the effect (or lack thereof) of well-documented factors, such as visual properties (contrast and colour) and speed-accuracy trade-off. Our work also documents robust individual differences in sensory conduction delays over 70 participant. Our conclusions are contrasted with those reached from simple decision models, such as the drift diffusion model and the linear ballistic accumulator, and highlights the need to better account for the influence of bottom-up signals and their dynamics in saccade generation.

DECISION-MAKING

BENNETT LECTURE THEATRE 8 - 10:20- 10:40

Uncertainty-driven Gaze Selection

Daniela Pamplona and Antoine Manzanera

ENSTA-Paris, France
daniela.pamplona@ensta.fr

The amount of visual information available in the environment is exorbitant and redundant. For real-time interaction with the world, embodied agents must select where and when to sample the information. This process is crucial for the well-being of animals, as ultimately, it can condition their survival. Generally, this sampling process considers various aspects: function, external state, internal state, and action motor costs. Here we focus on the agent's internal state, particularly on the uncertainty of its representations.

We postulate that those intrinsically motivated to be curious and decrease their uncertainty learn more efficiently than those who follow other motivations. To test this hypothesis, we consider an agent freely moving in a domestic virtual environment while learning efficient representations of its visual input. We compare the agent's model representation quality, coverage area, and learning efficiency when the agent is curious and selects gazing points to reduce the uncertainty of model parameters against baselines including random and bottom-up gaze selection strategies. We show that curiously sampling the environment improves the overall performance, but not equally for all uncertainty criteria. This work directly relates to the development of artificial systems, e.g. robots or avatars, and gives new insights into possible visual attention and eye movement mechanisms.

DECISION-MAKING

BENNETT LECTURE THEATRE 8 - 10:40- 11:00

Motivation by Reward Increases Performance Beyond the Speed-accuracy Tradeoff by Improving Distractor Suppression

Christian Wolf and Markus Lappe

University of Münster, Germany
chr.wolf@wwu.de

Saccadic selection is characterized by a tradeoff between speed and accuracy. Speed or accuracy of the selection process can be affected by higher-level factors, for example expecting a reward, obtaining task-relevant information, or seeing an intrinsically relevant target. Recently, it has been shown that motivation by reward can simultaneously increase speed and accuracy, thus going beyond the speed-accuracy-tradeoff. Here, we compared the motivating abilities of monetary reward, task-relevance and image content to simultaneously increase speed and accuracy of saccadic eye movements. We used a saccadic distraction task that required suppressing a distractor and selecting a target. Across different blocks successful target selection was followed either by (i) a monetary reward, (ii) obtaining task-relevant information, or (iii) seeing the face of a famous person. Each block additionally contained the same number of irrelevant trials lacking these consequences and participants were informed about the upcoming trial type. We found that only motivation by reward simultaneously increased speed and accuracy of the eye movement response. This was achieved by faster distractor suppression. Task-relevance increased speed but decreased selection accuracy, whereas post-saccadic vision of a face did neither affect speed nor accuracy, suggesting that image content does not affect saccadic selection via motivational mechanisms.

DECISION-MAKING

BENNETT LECTURE THEATRE 8 - 11:00- 11:20

Decision-making, Reward and Eye Movements

Eugene McSorley and Rachel McCloy

University of Reading, United Kingdom
e.mcsorley@reading.ac.uk

It has been reported that evidence used to support the decision to saccade to a target has a direct impact on that movement (McSorley & McCloy, 2009; McSorley et al, 2014). The path of a saccade has been found to deviate away from a non-selected target more as supporting evidence for the selected target improves. However, it is unclear what role reward plays in this process. To examine this, participants were asked to choose between two potential saccades targets as indicated by a briefly presented central motion coherence patch whose motion largely moved to toward one or the other. They were rewarded with points for correct choice and punished with points taken away for an incorrect answer. On occasion they could opt for a safe saccade target choice which gave a reward of a lower point value. It was found that performance improved as the motion coherence levels became higher, saccade latency decreased correspondingly, and the number of opt-out selection decreased. This shows that as the evidence supporting choice increases then the task becomes easier and participants' confidence increase (i.e., opt-out selections dropped). Overall saccade trajectories were found to be away from the non-selected target, showing that it has been inhibited in the target selection process. However, as motion coherence (the supporting evidence) increased, the saccade path was not found to deviate further away from the non-selected target. Thus, while the change in evidence supporting choice clearly influenced performance and eye movement control in terms of speed of response (percentage correct increase while opt-out choices decrease while saccade latencies decrease) the change did not impact on the spatial control of the saccade. We suggest that the reward associated with the choice, which does not change, rather than choice evidence itself, is the key factor which impacts on the inhibition of the non-selected target producing equivalent deviations in the path of the target directed saccade.

DECISION-MAKING

BENNETT LECTURE THEATRE 8 - 11:20- 11:40

What Drives Pupil Dilation during Decision-making – Surpise or Uncertainty?

Péter Pajkossy and Gábor Gesztesi

Department of Cognitive Science, Budapest University of Technology and Economics, Hungary
pajkossy.peter@ttk.bme.hu

Pupil dilation was linked previously to both environmental uncertainty and surprise. Importantly, in most cases these variables are correlated: more uncertain environments cause more surprising events, and in turn, surprise per se leads to uncertainty. We conducted two pupillometric studies to disentangle the effects of these factors on pupil dilation. In a probabilistic reversal task, participants engaged in a guessing game on computer, in which a fictional actor repeatedly hid a stone in one of his hands, and participants had to guess the location of the stone to attain reward. One of the locations was more often rewarded (e.g. stone was in 85% of the cases in the left hand) and the identity of this advantageous option was switched regularly (reversal). In Study 1, we varied uncertainty by changing reward probability linked to the advantageous option (e.g. 65% vs. 85%) and used a Bayesian model to estimate when participants detected a reversal (i.e. surprise). In Study 2, we investigated pupil dilation after we gave information about the preferred location in a specific manner, which enabled to disentangle the effects of uncertainty and surprise. Our results show that both uncertainty and surprise affects pupil size, but their effects can be dissociated.

DECISION-MAKING

BENNETT LECTURE THEATRE 8 - 11:40- 12:00

Error Inconsistency does not Generally Inhibit Saccadic Adaptation

Thomas Eggert, Katharina Kaltenbach and Andreas Straube

University Hospital, LMU Munich, Germany
eggert@lrz.uni-muenchen.de

Previous studies on adaptation of reaching movements observed that increased error inconsistency reduces the error sensitivity; the ratio between adaptive change and error size. Similar results were obtained in saccade adaptation raising doubts on the suitability of classical linear models of saccade adaptation.

We measured the gain of visually guided, horizontal saccades in 300 training trials with intrasaccadic target shifts (ITS) followed by a 300 washout trials. The adaptation dynamics were compared between two conditions (consistent/inconsistent) where the amplitude of the ITS formed a fixed/variable percentage of the primary target step. The mean ITS was identical in both conditions. The inter-trial standard deviation of the postsaccadic visual error was twice as large in the inconsistent than in the consistent condition.

The total adaptive changes during training or washout did not depend on error inconsistency. Initial adaptation speed was lower with inconsistent ITS. However, the effect on adaptation speed occurred only during amplitude reduction and not during enlargement or washout. It was also not sufficient to induce a significant effect on the adaptation time constant.

These results corroborate the linearity of saccade adaptation in that the mean error is the main factor determining the total adaptive change, independent of error consistency.

EYE-TRACKING METHODS

BENNETT LECTURE THEATRE 8 - 13:00- 13:20

Fixation Classification: How to Merge and Select Fixation Candidates

Ignace T. C. Hooge, Diederick C. Niehorster, Marcus Nyström, Richard Andersson and Roy S. Hessels

Utrecht University, Netherlands
i.hooge@uu.nl

Eye trackers are applied in many research fields. To give meaning to the eye-tracking data, researchers have a broad choice of classification methods to extract various behaviors (e.g., saccade, blink, fixation) from the gaze signal. There is extensive literature about the different classification algorithms. Surprisingly, not much is known about the effect of fixation and saccade selection rules that are usually (implicitly) applied. We want to answer the following question: What is the impact of the selection-rule parameters (minimal saccade amplitude and minimal fixation duration) on the distribution of fixation durations? To answer this question, we used eye-tracking data of high and low quality and seven different classification algorithms. We conclude that selection rules play an important role in merging and selecting fixation candidates. For eye-tracking data with good-to-moderate precision (RMSD < 0.5 deg), the classification algorithm of choice does not matter too much as long as it is sensitive enough and is followed by two rules: 1) select saccades with amplitudes > 1.0 deg, and; 2) select fixations with durations > 60 ms. Because of the importance of selection, researchers should always report whether they performed selection and the values of their parameters.

EYE-TRACKING METHODS

BENNETT LECTURE THEATRE 8 - 13:20- 13:40

Web-based Attention-tracking with an Eye-tracking Analogue is Reliable and Valid

Edwin S. Dalmaijer, Alexander L. Anwyl-Irvine and Thomas Armstrong

University of Bristol, United Kingdom
edwin.dalmaijer@bristol.ac.uk

Psychological research is increasingly moving to the internet, where larger and more diverse samples of participants can be reached. While much work has been done on webcam eye-tracking, the technique still suffers from high levels of attrition (~60%!) and imprecision. MouseView.js was developed to circumvent this issue. This software uses a mouse-locked aperture of high resolution (analogous to the fovea), while blurring the rest of a stimulus display. It can thus act as an analogue to eye tracking, for example to measure overt attention in online experiments. Here, we present findings of a validation study in which MouseView was compared directly with eye tracking (EyeLink 1000) in preferential looking tasks. We found that mouse-guided dwell time (collected via the internet) was at least as reliable as gaze dwell time (collected in the lab). In a second study, we show that there was a strong correlation between dwell time measured with MouseView and with eye-tracking (collected in the lab, within-participants). The only clear deviation between mouse-guided and gaze behaviour was in the first second of stimulus presentation, suggesting eye-tracking more accurately captured involuntary attention. We conclude that reliable and valid dwell data can be collected in web-based experiments using MouseView.js.

EYE-TRACKING METHODS

BENNETT LECTURE THEATRE 8 - 13:40- 14:00

Characterising Eye Movement Events with an Unsupervised Hidden Markov Model

Ingmar Visser, Šimon Kucharský and Malte Lüken

University of Amsterdam, Netherlands
i.visser@uva.nl

Eye-tracking allows researchers to infer cognitive processes from eye movements that are classified into distinct events. Parsing the events is typically done by algorithms. Here we aim at developing an unsupervised, generative model that can be fitted to eye-movement data using maximum likelihood estimation. This approach allows hypothesis testing about fitted models, next to being a method for classification. We developed gazeHMM, an algorithm that uses a hidden Markov model as a generative model, has few critical parameters to be set by users, and does not require human coded data as input. The algorithm classifies gaze data into fixations, saccades, and optionally postsaccadic oscillations and smooth pursuits. We evaluated gazeHMM's performance in a simulation study, showing that it successfully recovered hidden Markov model parameters and hidden states. Parameters were less well recovered when we included a smooth pursuit state and/or added even small noise to simulated data. We applied generative models with different numbers of events to benchmark data. Comparing them indicated that hidden Markov models with more events than expected had most likely generated the data. We also applied the full algorithm to benchmark data and assessed its similarity to human coding and other algorithms. For static stimuli, gazeHMM showed high similarity and outperformed other algorithms in this regard. For dynamic stimuli, gazeHMM tended to rapidly switch between fixations and smooth pursuits but still displayed higher similarity than most other algorithms. Concluding that gazeHMM can be used in practice, we recommend parsing smooth pursuits only for exploratory purposes. Future hidden Markov model algorithms could use covariates to better capture eye movement processes and explicitly model event durations to classify smooth pursuits more accurately.

EYE-TRACKING METHODS

BENNETT LECTURE THEATRE 8 - 14:00- 14:20

The Amplitude of Small Eye Movements can be Accurately Estimated with Video-based Eye-trackers

Marcus Nyström, Diederick C. Niehorster, Richard Andersson, Roy S. Hessels and Ignace T. C. Hooge

Lund University, Sweden
marcus.nystrom@humlab.lu.se

Estimating the gaze direction with a digital video-based pupil and corneal reflection (P-CR) eye tracker is challenging since 1) a video camera is limited in terms of spatial and temporal resolution, and 2) because the captured eye images contain noise. Through computer simulation, we evaluated the localization accuracy of pupil-, and CR centers in the eye image for small eye rotations (<<1 deg). We show how inaccuracies in center localization are related to 1) how many pixels the pupil and CR span in the eye camera image, 2) the method to compute the center of the pupil and CRs, and 3) the level of image noise. Our results provide a possible explanation to why the amplitude of small saccades may not be accurately estimated by many currently used video-based eye trackers. We conclude that saccades with arbitrarily small amplitudes can be accurately estimated using the P-CR eye-tracking principle given that the level of image noise is low and the pupil and CR span enough pixels in the eye camera, or if localization of the CR is based on the intensity values in the eye image instead of a binary representation.

EYE-TRACKING METHODS

BENNETT LECTURE THEATRE 8 - 14:20- 14:40

Event-level Evaluation of Eye Movement Event Detectors

Raimondas Zemblys and Mikhail Startsev

Smart Eye AB, Sweden
raimondas.zemblys@gmail.com

Dozens of eye movement event detectors exist to date, however the reported results of performance evaluation are usually neither directly comparable between the papers nor easily interpretable even by the field experts. To a large degree it is a direct consequence of the multitude of available evaluation methods and approaches. The number of reported metrics alone is impressive (sensitivity/specificity/F1 scores, accuracy or disagreement rates, Cohen's kappa, etc.), while the details of their application and implementation, lead to fundamental dissimilarities even when the same metric is used in the evaluation. This is especially prominent when considering event level evaluation. In this talk we review existing practices of evaluating eye movement event detection algorithms and present the empirical analysis of different combinations of eye movement event matching methods and metrics computed on the results of the matching step. We also give recommendations on improving the event detection evaluation pipeline that aim to ensure the high quality of future publications, as well as encourage inter-comparability and reproducibility in this field of research.

EYE-TRACKING METHODS

BENNETT LECTURE THEATRE 8 - 14:40- 15:00

Eye-tracking: Empirical Foundations for a Minimal Reporting Guideline

Roy S. Hessels, Kenneth Holmqvist, Saga Lee Örbom, Ignace T. C. Hooge, Diederick C. Niehorster, Robert G. Alexander, Richard Andersson, Jeroen S. Benjamins, Pieter Blignaut, Anne-Marie Brouwer, Lewis L. Chuang, Kirsten A. Dalrympleke, Denis Drieghe, Matt J. Dunn, Ulrich Ettinger, Susann Fiedler, Tom Foulsham, Jos N. van der Geest, Dan Witzner Hansen, Samuel B. Hutton, Enkelejda Kasneci, Alan Kingstone, Paul C. Knox, Ellen Kok, Helena Lee, Joy Yeonjoo Lee, Jukka M. Leppänen, Stephen Macknik, Päivi Majaranta, Susana Martinez-Conde, Antje Nuthmann, Marcus Nyström, Jacob L. Orquin, Jorge Otero-Millan, Soon Young Park, Stanislav Popelka, Frank Proudlock, Frank Renkewitz, Austin Roorda, Michael Schulte-Mecklenbeck, Bonita Sharif, Frederick Shic, Mark Shovman, Mervyn G. Thomas, Ward Venrooij and Raimondas Zemblys
Utrecht University, Netherlands
R.S.Hessels@uu.nl

In this paper, we present a review of how the various aspects of any study using an eye tracker (such as the instrument, methodology, environment, participant, etc.) affect the quality of the recorded eye-tracking data and the obtained eye-movement and gaze measures. We take this review to represent the empirical foundation for reporting guidelines of any study involving an eye tracker. We compare this empirical foundation to five existing reporting guidelines and to a database of 207 published eye-tracking studies. We find that existing reporting guidelines vary substantially and do not match with actual reporting practices. We end by deriving a minimal, flexible reporting guideline based on empirical research. This is an international collaboration involving 46 authors.

REAL WORLD AND VIRTUAL REALITY

BENNETT LECTURE THEATRE 8 - 16:30- 16:50

Characterization of Naturalistic Free-viewing Behavior Across the Lifespan

Rachel Yep, Brian White, Heidi Riek, Olivia Calancie, Ryan Kirkpatrick, Julia Perkins, Matthew Smorenburg, Donald Brien, Brian Coe, Laurent Itti and Douglas Munoz

Queen's University Centre for Neuroscience Studies, Canada
12ry@queensu.ca

Visual attention changes as we age, reflecting underlying maturational and degenerative processes in brain structure and function. Previous work investigating visual attention in aging is limited by the characterization of developmental and aging processes separately, and the reliance on task paradigms which fail to capture the visual complexity of the real world. The use of dynamic videos during unstructured free viewing provides a more ecologically valid means of investigating visual attention in individuals of different ages. The goal of the present study is to characterize naturalistic free viewing behaviour across the lifespan. We recorded saccade behavior from a large, cross-sectional cohort of normative individuals (n=497, aged 5-89) while they freely viewed naturalistic video clips that changed in content every 2-4 s. Averaged across clips, saccade amplitude and peak velocity decreased with age, while saccade frequency increased with age. Aligned to clip onset, the timing and magnitude of saccade rate and gaze clustering exhibited complex curvilinear trajectories with age. We propose that the trajectories of these saccade behaviors are mediated by structural and functional changes in underlying cortical and subcortical circuits. These findings have considerable implications for improved detection of neurological disorders that emerge during vulnerable windows of development and aging.

REAL WORLD AND VIRTUAL REALITY

BENNETT LECTURE THEATRE 8 - 16:50- 17:10

Visual Stability in Naturalistic Scenes

Jessica Parker and Caglar Tas

University of Tennessee-Knoxville, United States
jparke87@vols.utk.edu

The current study examines how visual stability is established in naturalistic scenes. Previous studies have shown that detection of position shifts is better when the saccade target object shifts rather than the background or the whole image shifting (Currie et al., 2000). Additionally, briefly removing the target object from the screen (blanking) improves shift detection (Deubel et al., 1996). We tested whether blanking would improve shift detection for contextual information in naturalistic scenes. Participants were presented with images and instructed to execute a saccade to a highlighted target object. During the saccade, the saccade target, the whole image, or the background shifted. In control trials, no shift occurred. Half of the trials had a 250ms target blank, context blank, or all blank that occurred when the saccade was detected. Participants reported whether they detected a move. Target shifts resulted in the highest detection rate, and the background shifts had the lowest detection rate. More importantly, we found that blanking only improved shift detection in target shift condition but not in background or whole image shifts, which suggests that the visual system uses a localized solution for establishing object correspondence across saccades that mainly relies on the saccade target for stability.

REAL WORLD AND VIRTUAL REALITY

BENNETT LECTURE THEATRE 8 - 17:10- 17:30

Finding Landmarks – An Investigation of Viewing Behavior during Spatial Navigation in VR Using a Graph-theoretical Analysis Approach

Jasmin L. Walter, Lucas Essmann, Sabine König and Peter König

University of Osnabrück, Germany
jawalter@uos.de

Vision provides the most important sensory information for spatial navigation. Recent technical advances allow new options to conduct more naturalistic eye tracking experiments in virtual reality (VR), but also require new analysis approaches. Here, we propose a method to quantify characteristics of visual behavior by applying graph-theoretical measures to eye tracking data.

The analysis is based on eye tracking data of 20 participants, who freely explored a virtual city for 90 minutes with an immersive VR headset with an inbuild eye tracker. We pre-process the data and define "gaze" events, from which we created gaze graphs. On these, we applied graph-theoretical measures to reveal the underlying structure of visual attention.

To investigate the importance of houses in the city, we apply the node degree centrality measure. Our results reveal 10 houses consistently outstanding in their graph theoretical properties. As these outstanding houses fulfilled several characteristics of landmarks, we named them "gaze-graph-defined landmarks". Furthermore, we find that these gaze-graph-defined landmarks were preferentially connected to each other.

Our findings do not only provide new experimental evidence for the development of spatial knowledge, but also establish a new methodology to identify and assess the function of landmarks based on eye tracking data.

REAL WORLD AND VIRTUAL REALITY

BENNETT LECTURE THEATRE 8 - 17:30- 17:50

An Online Experiment with Deep-learning Models for Tracking Eye Movements via Webcam

Shreshth Saxena, Lauren K. Fink and Elke B. Lange

Max Planck Institute for Empirical Aesthetics, Germany
shreshth.saxena@ae.mpg.de

Eye-tracking during online experiments allows collection of larger and more diverse datasets in a less restrictive and cost-effective manner. Computer vision methods that can track eye movements from webcam recordings have improved remarkably with the application of deep learning. However, their application is still restricted to offline setups due to the computation requirements and difficulty of estimating physical calibration parameters (e.g., camera intrinsics and user-camera distance). Here, we embrace these challenges by designing an online experiment with robust solutions for such constraints. The experiment consisted of 65 participants completing a battery of five eye-tracking tasks that we used to compare three state-of-the-art appearance-based gaze tracking methods and two blink-detection algorithms. We report on multiple measures, such as fixation accuracy, precision, smooth pursuit onset and angle, attended zone classification, saliency mapping, etc. and also evaluate different calibration strategies. Our results demonstrate a mean fixation error in the range of 2-3 visual degrees for the best model. While these errors are not as low as Eyelink100 (0.57°) and Pupil Core (0.82°) on these same tasks, they nonetheless encourage the use of unrestricted setups for accurate eye tracking in online studies (compare to e.g. 4.17° from Webgazer.Js).

The pre-registration for our study could be found at https://osf.io/qh8kx/.

REAL WORLD AND VIRTUAL REALITY

BENNETT LECTURE THEATRE 8 - 17:50- 18:10

Georeferencing of Eye Movement Data using ET2Spatial Software

Stanislav Popelka, Minha Noor Sultan and Josef Strobl

Department of Geoinformatics, Palacky University Olomouc, Czechia
standa.popelka@gmail.com

Eye-tracking in cartography has been active in the last few decades, particularly as the main pillar of cognition in geographic visualization. Stimuli used in this field of research are mostly static maps. With the evolution of techniques in geovisualization and technology itself, however, these stimuli have also evolved from analogue to digital and from static to dynamic. The interactive stimuli hence pose challenges when it comes to evaluating their usability through eye-tracking.

The submission aims to introduce a developed open-source tool called ET2Spatial intended for the analysis of eye-tracking data recorded on interactive web maps. The tool simplifies the labour-intensive task of analysis of screen recordings with overlaid eye-tracking data available in current eye-tracking systems. The tool's main function is to convert the screen coordinates of the participant's gaze to real-world coordinates and allow exports in commonly used spatial data formats (shapefile, geojson). These data can be loaded into Geographic Information System (GIS) software, where different visualization and analytical methods commonly used for spatial data can be applied. The tool and associated pilot studies aim to enhance the research capabilities in eye-tracking in cartography.

POSTER SESSION II, 1 ATTENTION

STUDENT UNION SQUARE HALL - 15:00- 16:30

Seeing Your Own Webcam Image Feels Distracting, But Does Not Hurt Learning: A Webcam-based eEye-tracking Study

Gesa van den Broek, Vincent Hoogerheide and Ellen Kok

Utrecht University, Netherlands
g.s.e.vandenbroek@uu.nl

In video-call platforms like Zoom or Microsoft teams, a thumbnail of one's own webcam video feed is often visible. In this experiment, we use webcam-based eye tracking to investigate whether this self-view distracts learners and reduces learning from a video lecture. In a within-subjects experiment, 78 participants (Mage = 22.3) watched two video lectures, one with and one without the self-view visible (in counterbalanced order). Webcam-based eye-tracking was implemented using the Gorilla Experiment Builder (www.gorilla.sc) and participants took part on their own computers. Participants first completed a 9 point calibration and 5 point validation procedure. They then watched the instructional video (either with the self-view visible or not), rated their experience of learning, and completed a post-test. This procedure was repeated for the other condition. Bayesian statistics were used to analyse the data. Participants felt more distracted and self-conscious in the self-view versus the no self-view condition. However, the conditions were comparable in self-reported mental effort and post-test performance. Analysis of the webcam-based eye-tracking data shows limited effects of the self-view on viewing behaviour. In this presentation, we additionally discuss the quality of webcam-based eye-tracking data for stimuli with very large AOIs.

POSTER SESSION II, 2 ATTENTION

STUDENT UNION SQUARE HALL - 15:00- 16:30

Attentional Biases in the Size of Fixational Saccades

Baiwei Liu, Anne Zonneveld and Freek van Ede

Vrije Universiteit Amsterdam, Netherlands
b.liu@vu.nl

It is well established that spatial attention can bias the direction of fixational saccades. It remains unknown whether and when attention may also bias the size (amplitude) of fixational saccades. To investigate this, we cued attention to one of multiple items in visual working memory while manipulating the spatial demands on attention. In one condition, trials contained two colored tilted bars (presented to the left and right) that were both either near or far from fixation. A color cue (presented during the delay) instructed participants to select either memorandum. Additionally, we included a load-four condition in which items occupied the near and far locations on both sides. Critically, in these trials, the direction is not sufficient to select the cued memorandum as there are always two memoranda in the same direction (one near, one far). Consistent with prior work, we confirm that the direction of fixational saccades was robustly biased by the memorized location of the attended memorandum. More importantly, our data reveal that the size of the directionally biased fixational saccades was also modulated by the spatial demands on attention. Specifically, fixational saccades became larger when selecting the far item, but only when direction was insufficient.

POSTER SESSION II, 3 ATTENTION

STUDENT UNION SQUARE HALL - 15:00- 16:30

Eye Movements in Three-dimensional Multiple Object Tracking

Qi Zhang, Juntian Lin, Man Jia, Jie Li and Yu Zhang

School of Psychology, Beijing Sport University, China, China
15537841357@163.com

Eye movements in multiple object tracking (MOT) tasks reflect the attention processing of an observer. Previous studies have revealed two gaze strategies during two-dimensional MOT tasks, respectively centroid-looking strategy and target-switching strategy. When tracking several moving targets amidst distractors in an MOT task, observers are more likely to gaze at the central areas between targets, and would frequently switch their gaze back and forth between the center and targets. However, little research has focused on where are observers looking and what influences eye movements during the three-dimensional multiple object tracking (3D-MOT). The present study registers eye movements by using a 3D-MOT task based on virtual reality technology, which could exquisitely reflect the interaction between humans and the real world with stereo vision. The aim of the present study is to examine observers' looking strategy in 3D-MOT, how it is affected by the depth of the 3D spaces, and how differs from the eye behaviors in 2D tracking. We postulate that the target-switching strategy would be in preference in 3D-MOT and observers would switch fixations more frequently to targets at greater depth in the 3D spaces.

POSTER SESSION II, 4 ATTENTION

STUDENT UNION SQUARE HALL - 15:00- 16:30

Modeling Task-Dependency of Eye Movement during Scene Viewing

Lisa Schwetlick, Daniel Backhaus and Ralf Engbert

University of Potsdam, Germany
lisa.schwetlick@uni-potsdam.de

Eye movements during scene viewing reflect how the visual system processes and prioritizes information. Selection of fixation locations is driven by image features, as well as by top-down factors, and task constraints. Additionally, dynamical aspects such as scan path history and fixation duration influence selection. The SceneWalk model is a biologically inspired dynamical model for predicting fixation sequences on the basis of static saliency maps. In the current work we added a spatial-temporal likelihood to the existing framework to jointly model fixation durations and location selection. To explore the influence of task and saliency, we investigated two model versions, using either general- or task-specific saliency maps as a basis. We separately fitted model parameters for each individual in two guessing and two searching tasks. Parameters were inferred using a fully Bayesian likelihood-based approach. We find that the optimal parameters differ significantly between the tasks. The parameters of our model represent interpretable quantities such as the attention span. Therefore, differences in parameters allow insight into how the visual system adapts to task demands. Posterior predictive checks show that the model can reproduce individual differences in scan path statistics. We also show that dynamic components of eye movement improve model fit more than task-specific saliency.

POSTER SESSION II, 5 ATTENTION

STUDENT UNION SQUARE HALL - 15:00- 16:30

Using Eye-Tracking Techniques for Oculomotor Signs of Neglect

Marina Shurupova, Kseniya Lizunkova, Alina Aizenshtein, Anna Dreneva and Alexander Latanov

Federal Center of Brain and Neurotechnologies, Department of Rehabilitation, Russia
shurupova@fccps.ru

The common outcome after a right hemisphere stroke is unilateral spatial neglect. These patients demonstrate visual attentional deficits towards to the contralesional side. The aim of our pilot study was to investigate oculomotor signs of neglect using a new experimental paradigm.

Six patients (mean age 54.5±9.8) and twelve age-matched controls participated in the study. Patients had subacute phase of stroke (1 hemorrhagic, 5 ischemic) and were diagnosed with neglect by a neuropsychologist. Patients performed an oculomotor task in which they had to select and fix a target stimulus (a blue star) appearing simultaneously with a distracting factor (a black dot) to the left or right of it. Eye movements were recorded at 250 Hz sample rate.

Patients showed a higher error rate when the target appeared on the left, although most patients made self-correction within two seconds after choosing a distractor. In addition, patients showed a higher delay to the appearance of the target on the left compared to the right. All patients needed more than one fixation to fix the target, while the control group needed one.

Our experimental results demonstrate oculomotor signs of neglect. Future studies will add more quantitative results and may be applicable in clinical practice.

POSTER SESSION II, 6 ATTENTION

STUDENT UNION SQUARE HALL - 15:00- 16:30

Oculomotor Control and Dual-Task Interference

Aleks Pieczykolan and Lynn Huestegge

Rheinische Fachhochschule Köln, Germany
aleks.pieczykolan@gmail.com

For a long time, oculomotor control was regarded as largely unaffected by additional actions in other effector modalities. However, recent research suggested that saccade control - although being prioritized over other simultaneous actions - can still exhibit substantial impairments. In the present study, we examined temporal dynamics of oculomotor performance decrements in dual tasks by applying the psychological refractory paradigm, in which we varied the stimulus onset asynchrony (SOA) between a saccade and a manual RT task. Across 4 experiments we examined differential effects of task order, task order instructions and spatial task compatibility on dual-task performance.

Results revealed that performance of both saccade and manual responses suffered at close temporal proximity indicating structural and content-based interference mechanisms. Structural interference was observed in form of longer RTs for Task2 at short SOAs suggesting a serial response selection bottleneck, and content-based interference emerged as longer RTs for incompatible than for compatible tasks suggesting mutual crosstalk during serial processing.

Based on these results, we reject the notion that oculomotor control is generally able to bypass central processing limitations and instead conclude that saccades are subject to the same sources of dual-task interference mechanisms as other actions, too.

POSTER SESSION II, 7 MEMORY

STUDENT UNION SQUARE HALL - 15:00- 16:30

Testing Memory Strength with Pupil Dilation as a Function of Strategic and Automatic Memory Retrieval

Ádám Albi and Péter Pajkossy

Department of Cognitive Science, Budapest University of Technonogy and Economics, Hungary
a.adam.munka@gmail.com

Previous research indicates that pupil responses during automatic recognition of previously seen information reflect the aggregate strength of a memory trace and not cognitive effort per se. In contrast, during recall or effortful recollection, the retrieval process is predominantly reliant on strategic processes and cognitive effort, as an active searching strategy is needed for successful retrieval. In such cases, consequently, we expected that as the strength of the memory trace increases, fewer mental effort is needed for the retrieval resulting in a negative link between memory strength and pupil dilation. Thus, memory strength might be differently related to pupil dilation in different forms of memory tests. To test this hypothesis, we implemented two testing paradigms on verbal stimuli: a paired-associate learning paradigm and the source-monitoring framework. We manipulated memory strength by presenting words different times (one vs. four times) on different spatial locations in source monitoring and by presenting word-pairs different times (one vs two times) in the paired-associate learning. In the subsequent memory test, we tested them on recognition and recall/recollection and measured pupil responses. Our preliminary results suggest that the link between memory strength and pupil dilation is modulated by the form of retrieval.

POSTER SESSION II, 8 MEMORY

STUDENT UNION SQUARE HALL - 15:00- 16:30

Eye-tracking in Innovative Neuropsychological Assessment of Visual Working Memory

Sanne Böing, Teuni Ten Brink, Alex Hoogerbrugge, Tanja Nijboer and Stefan Van der Stigchel

Utrecht University, Netherlands
s.boing@uu.nl

In both the laboratory and clinical neuropsychological assessment, visual working memory (VWM) is typically estimated by means of the maximum storage load. However, these assessment settings ignore that in daily life, information is generally available in the external world. We can easily sample information from the environment by making eye-movements, reducing the need to use the maximum VWM storage load. Vice versa, reliance on VWM capacity increases when accessing external information is difficult or costly. We investigated whether people reduce VWM load when sampling is possible, and whether they memorize more information when sampling is costly. Patients with severe memory problems (Korsakoff's syndrome) and controls were instructed to perform a copy task while their eyes were tracked. The availability of the example puzzle was manipulated by introducing a gaze-contingent waiting time to provoke different strategies (sampling vs. storing). Preliminary data confirms that controls successfully shifted from sampling to storing when information became less readily available. Although patients also showed less sampling indicating an attempt to adjust strategy, they could not memorize more items at once and made more errors. This suggests that successfully switching strategy from sampling to storing is dependent on VWM functionality.

POSTER SESSION II, 9 MEMORY

STUDENT UNION SQUARE HALL - 15:00- 16:30

Pupil Responses: Indices of Individual Memory Performance

Gábor László Bényei and Péter Pajkossy

Department of Cognitive Science, Budapest University of Technology and Economics, Hungary
benyeig@edu.bme.hu

Studies have suggested that pupil size changes reflect activity of the Locus Coeruleus (LC). Thus, by measuring fluctuations in pupil diameter over time, we can indirectly monitor ongoing attentive processes. An ample number of pupillometry studies have already investigated within-subject effects. In contrast, less research has focused on exploring how individual differences in pupil responses correlate with criterion variables. To this aim, in our present research, we inspected between-subjects variabilities in phasic pupil responses as possible predictors of individual memory performance. In one experiment we used an incidental memory task targeting mnemonic discrimination (Mnemonic Similarity Task), whereas in the other task, a 2-back design was used. We had the participants' pupils recorded during both tasks. For our correlational analyses, we measured baseline corrected event-related pupil dilation (ERPD). We conclude that individual differences in task-evoked pupil behavior can be used to predict cognitive performance. This might be caused by the modulating role of LC on attentional processes.

POSTER SESSION II, 10 MEMORY

STUDENT UNION SQUARE HALL - 15:00- 16:30

The When and Where of the Looking at Nothing Effect: Examining Eye Movements During Memory Retrieval

Ruhi Bhanap, Klaus Oberauer and Agnes Rosner

University of Zurich, Switzerland
ruhi.bhanap@psychologie.uzh.ch

Looking at Nothing (LAN) describes the behavior that people look at empty spatial locations when trying to retrieve information from memory which was previously associated to these locations. This study investigated LAN for retrieval from working memory. We tested whether LAN is directed to all or only some of the associated spatial locations, when LAN occurs, and its relation to retrieval performance. During encoding, participants saw four word-pairs in four different spatial locations on a screen. During retrieval, they heard two words and had to indicate whether the words came from one previously seen word-pair (positives) or from two different pairs (lures). We found that participants only showed LAN to the first probe's location, but this occurred only when hearing the second probe, irrespective of the correctness of the response. The results speak in favor of memory processes leading to LAN during the recollection of information in working memory.

POSTER SESSION II, 11 MEMORY

STUDENT UNION SQUARE HALL - 15:00- 16:30

The Context Effect on Implicit Sequence Learning Using an Ocular Version of the Serial Reaction Time (O-SRT) Task

Eli Vakil and Simone Schwizer Ashkenazi

Bar Ilan University, Israel
vakile@biu.ac.il

Objectives: We aimed to evaluate the effect of contextual information on implicit sequence learning (ISL) using an ocular version of the Serial Reaction Time task (O-SRT).

Participants and Methods: A total of 76 young adults were tested on the O-SRT using two alternating sequences simultaneously. Participants were randomly assigned to one of two versions of the task: with or without context. In the former each one of the two sequences was presented with a different context (shape and color), and in the latter both sequences were presented with the same context. Eye movements were recorded by an SMI-RED eye-tracker (250Hz).

Results: Correct Anticipations of next spatial location were analyzed applying Mixed Design ANOVA, with Group (with and without context) and Learning trials (1-6), between and within-subjects factors, respectively. The group with context showed significant learning in the later trials of training, compared to the group without context that showed significant improvement in the earlier trials.

Conclusions: Contextual information might impede ISL in the early learning phase, possibly because it fosters explicit exploration of task or because it requires the processing of additional information, compared to the no context condition, which is proved to be beneficial for the later learning stages.

POSTER SESSION II, 12 METHODS

STUDENT UNION SQUARE HALL - 15:00- 16:30

eyetRack - Shiny Application for Recurrence Quantification Analysis

Veronika Kalabusova, Kamila Facevicova and Stanislav Popelka

Palacký University Olomouc, Czechia
w.kalabusova@seznam.cz

eyetRack is a new R package and a Shiny application which facilitates the accessible analysis of eye-tracking data from SMI or Tobii eye-trackers. It offers a basic analysis for the initial conception of the number and duration of fixations. Barplot visualization allows to show a number of fixations in each Area of Interest and Dwell Time. The tool also offers visualization of the scanpath above the stimulus. The essential functionality of the application is the analysis through recurrence and recurrence quantification analysis. The recurrence plot can be displayed. However, visualization of recurrent fixations can often predispose to subjective bias when evaluating a set of results. For that reason, we used recurrence quantification analysis measures, which allow us to quantify data displayed in the recurrence plot. Using RQA, we can compare different tasks or compare multiple participants. The last functionality of the application is the calculation of coefficient K, which helps distinguish focal and ambient attention. The tool will be freely available at www.eyetracking.upol.cz/tools.

POSTER SESSION II, 13 METHODS

STUDENT UNION SQUARE HALL - 15:00- 16:30

An Open-source Device for Vestibular Stimulation and Eye-movement Tracking in Head-fixed Mice

Alexandra Tran Van Minh, Xavier Cano-Ferrer and Ede Rancz

The Francis Crick Institute, United Kingdom
alexandra.tranvanminh@crick.ac.uk

Visual virtual reality (VR) is widely used to study cortical processing in awake, behaving mice. It allows for tight control of animal-driven visual stimuli and provides the ability to change the coupling between behaviour and visual stimulus. However, most visual VR approaches render animals motionless in space (i.e., head-fixed), resulting in the vestibular system being taken out of play. Consequently, the head-direction (HD) system, which is primarily driven by vestibular input and plays a pivotal role in navigation, is severely compromised.

Here we present a novel experimental apparatus to overcome this limitation. Using an open-source approach, we have built a modular and affordable device allowing the rotation of head-fixed, behaving mice. It can be used in open-loop mode to study vestibular sensory representation and processing. In closed-loop mode, the apparatus allows animals to navigate in rotational space and self-generate vestibular input, providing a better substrate for 2D navigation in virtual environments.

We show that our approach is compatible with the electrical recording of brain activity at the cellular level and results in the robust recruitment of HD cells. We further demonstrate its utility by combining the tracking of vestibular and visually evoked eye-movements with optogenetic interference of specific neuronal populations.

POSTER SESSION II, 14 METHODS

STUDENT UNION SQUARE HALL - 15:00- 16:30

Yarbus in the Age of Webcam Eye-tracking

Divya Seernani, Amandine Grappe, Andrew Korepanov, Kåre Jensen, Kerstin Wolf, Jessica Wilson and Nadia Pedersen

iMotions, Denmark
divya.seernani@imotions.com

The iMotions webcam based eye tracking (WebET), combined with the hidden markov models (HMM) for fixations classification is a potential tool for applied human behaviour research and teaching eye-tracking to larger cohorts. The present study explores the feasibility of carrying out large scale, replicable research projects.

To verify how relevant the iMotions WebET can be to applied research and teaching, a part of the Yarbus (1967) study was replicated with N=10 participants. In an online study, participants were shown 'The Unexpected Visitor' in four conditions in a repeated measures design, each one asking a different question of the participants. Individual scanpaths and aggregated heatmaps were used for exploratory analysis. As in the seminal work by Yarbus, the present study asked if WebET can be used to distinguish where people look based on the question asked. Areas of interest (AOIs) were marked and calculated in iMotions to understand best practices for quantifying data from webET.

Results show that even with a small sample size, the iMotions WebET combined with the HMM fixation classification can accurately distinguish between scanpaths of different conditions. Larger, well-placed AOIs can give eye-tracking insights helpful in understanding top-down cognitive processing of participants in the present study

POSTER SESSION II, 15 METHODS

STUDENT UNION SQUARE HALL - 15:00- 16:30

GlassesValidator: Data Quality Tool for Eye-tracking Glasses

Diederick C. Niehorster, Roy S. Hessels, Jeroen S. Benjamins, Marcus Nyström and Ignace T. C. Hooge

Lund University, Sweden
diederick_c.niehorster@humlab.lu.se

According to the proposal for a minimum reporting guideline for an eye tracking study by Holmqvist et al. (2022), the accuracy (in degrees) of eye tracking data should be reported. Currently, there is no easy way to determine accuracy for wearable eye tracking recordings. To enable determining the accuracy quickly and easily, we have produced a validation poster and written accompanying Python software. Here we present this work.

We tested the poster and procedure with 61 subjects. In addition, the software has been tested with six different wearable eye trackers. The validation procedure can be administered within a minute per subject and provides accuracy, precision and data loss. Calculating the eye tracking data quality measures can be done offline on a simple computer and requires no advanced computer skills.

POSTER SESSION II, 16 METHODS

STUDENT UNION SQUARE HALL - 15:00- 16:30

A Field Test of Appearance-based Gaze Estimation

Niilo Valtakari, Roy S. Hessels, Diederick C. Niehorster, Chantal Kemner and Ignace T. C. Hooge

Utrecht University, Netherlands
niilovaltakari@gmail.com

Appearance-based gaze estimation (ABGE) refers to techniques that estimate gaze direction from video recordings of the eyes or face. Although many ABGE methods have been developed, most of their validations can only be found in the technical literature (e.g., computer science conference papers).

We aimed to 1) identify which ABGE methods are usable by the average experimental psychologist, and 2) validate those methods. We searched the existing literature for methods that don't require calibration and have clear documentation. Only OpenFace and OpenGaze were found to fill these criteria. We evaluated the methods by having adult participants fixate points displayed on a screen for three conditions with different degrees of head movement.

We demonstrate that (1) gaze estimation sufficed to distinguish between all fixated points for some but not all participants, (2) there was large variability in the accuracy and precision of gaze estimation, (3) gaze estimates were not independent from head orientation, and (4) OpenGaze outperformed OpenFace. We conclude that both methods can potentially be used in sparse environments with horizontally separated areas of interest.

POSTER SESSION II, 17 READING

STUDENT UNION SQUARE HALL - 15:00- 16:30

Interactive Effects of Semantic Diversity and Word Frequency in Natural Reading

Neslihan Caliskan, Sara Milligan, Milca Jaime Brunet and Elizabeth Schotter

University of South Florida, United States
nescaliskann@gmail.com

Word frequency exhibits one of the strongest influences on reading behavior - increasing skipping rates and reducing fixation durations (Rayner, 1998). However, some have argued that semantic/contextual diversity better represents word difficulty (Adelman et al., 2006) and has a stronger effect on fixation behavior when frequency is controlled (Plummer et al., 2013). We investigated whether these factors influence the reading process differently across the time course of reading behavior. We performed a secondary data analysis on a sentence reading study with target words that ranged in frequency and semantic diversity. We found that only word frequency affected skipping rates. However, there was an interaction between word frequency and contextual diversity wherein high frequency words were read faster when they were low in contextual diversity. Conversely, low frequency words were read faster when they were high in contextual diversity. This suggests that, for familiar words, having a specific meaning facilitates word recognition whereas for unfamiliar words, having more diverse semantic features makes recognizing at least one of those meanings more accessible. Our findings support prior literature by arguing that word frequency facilitates early stages of word recognition prior to meaning retrieval while semantic diversity influences more fine-grained semantic processing downstream.

POSTER SESSION II, 18 READING

STUDENT UNION SQUARE HALL - 15:00- 16:30

Individual Differences in Word Learning Associated with Reading Skill and Vocabulary: An Eye-movement Investigation

Emily J. Bellerby, Sara Milledge, Kristofor McCarty and Hazel Blythe

Department of Psychology, Northumbria University, United Kingdom
emily.bellerby@northumbria.ac.uk

A large proportion of an individual's vocabulary is learned incidentally, during reading. We examined individual differences in lexical acquisition during reading, and whether low-frequency words are processed differently to pseudowords during lexical acquisition. Rigorous pre-screening ensured that the low-frequency words were not known by our target population. Participants' eye-movements were measured as they read sentences containing unknown words (either low-frequency or pseudowords) in a learning phase and a subsequent test phase. First, each new word was presented in four meaningful sentences during the learning phase, providing a diverse semantic context. We then took individual assessments of both reading ability and vocabulary. In the test phase, each new word was presented in a further four meaningful sentences, and reading time measures provided an index of the ease with which participants were able to read the new words. Finally, participants completed a semantic categorisation task to examine whether semantic representations for the new words had been successfully formed. We predict that greater reading skill and larger vocabulary sizes, will be associated with more efficient lexical acquisition. We also predict that there will be no differences between low-frequency words and pseudowords, validating the use of pseudowords within word learning experiments.

POSTER SESSION II, 19 READING

STUDENT UNION SQUARE HALL - 15:00- 16:30

GECO-CN: Ghent Eye-Tracking COrpus of Sentence Reading for Chinese-English Bilinguals

Longjiao Sui

Ghent University, Belgium
caroline.sui1989@gmail.com

GECO-CN presents the very first eye-tracking corpus of Chinese-English bilinguals reading a novel in their two languages. Participants read half of the novel in Chinese as first language and the rest in English as second language, in a counterbalanced order. They also completed a series of language proficiency tests and a language background questionnaire (LEAP-Q). This work presents some important descriptive statistics and compares the reading performance of two languages on eye movement measures such as average reading times and skip rate. In addition, this study used the same reading material as GECO (Cop et al., 2017), which studies the performance of Dutch-English bilinguals. By comparing two bilingual eye-movement corpora in which the similarity of the second language to the native language is different, this corpus is useful to investigate the influence of different Eastern and Western first languages on reading in the second language. This unique eye-tracking corpus will be freely available online, enabling future research to examine theories of bilingual reading by investigating similarities, differences, and mutual influences between two different writing systems.

POSTER SESSION II, 20 READING

STUDENT UNION SQUARE HALL - 15:00- 16:30

The Role of Phonological and Orthographic Parafoveal Processing during Silent Reading in Russian Children and Adults

Vladislava Staroverova, Anastasiya Lopukhina, Nina Zdorova, Nina Ladinskaya, Olga Vedenina, Sofya Goldina, Anastasiia Kaprielova, Ksenia Bartseva and Olga Dragoy

V.M. Bekhterev National Research Medical Center of neurology and psychiatry, Russia
vika06.95@mail.ru

Parafoveal processing allows readers to recognize a word before fixating on it. However, there is still a debate about the type of information that people might get from the parafovea. Studies have shown that adults and children use phonological and orthographic parafoveal processing, but their role depends on age and language. In the present study, we investigated the development of phonological and orthographic parafoveal processing during silent reading in 56 Russian-speaking second graders, 48 fourth graders, and 65 adults. The participants read sentences with embedded target nouns, while their eye movements were recorded in a gaze-contingent boundary paradigm. The target nouns were presented in the parafovea in original, pseudohomophone, control for pseudohomophone, transposed-letter and control for transposed-letter conditions. The comparison of fixation durations between the conditions allowed us to assess the reliance on phonological and orthographic information in each age group. We found that adults used both phonological and orthographic information from the parafovea, whereas second graders and fourth graders relied on orthographic parafoveal information. These results might indicate that Russian-speaking children do not have fully developed phonological recoding skills by grade 4, but can recognize a word in the parafovea as a whole orthographic unit already in grade 2.

POSTER SESSION II, 21 READING

STUDENT UNION SQUARE HALL - 15:00- 16:30

Reading Search Page Results: Evidence from an Eye-tracking Study on 11-12-year-olds

Oksana Kanerva, Tuomo Häikiö, Johanna K. Kaakinen and Mirjamaija Mikkilä-Erdmann

University of Turku, University of Helsinki, Finland
oksana.kanerva@helsinki.fi

This study examined whether 11-12-year-old Finnish readers can differentiate task-relevant search page results from the irrelevant ones. The participants (N=34) read simulated search engine results pages (SERPs) while their eye movements were recorded. Each page included 8 search hits, which were described with a title and a short description, which could be either relevant or irrelevant to the search task given to the readers. The position of the relevant search results on the search page was manipulated, and average reading speed in a separate reading task was used as a measure of reading skill. The results of the fixation time on titles showed that skillful readers spent less time reading the titles towards the end of the search page, regardless of relevance. Less skilled readers did not show such a speed-up. As for the fixation time on descriptions, skilled readers spent less time on irrelevant segments towards the end of the pages, whereas the fixation time for relevant segments did not change as much. Less skilled readers' fixation times on irrelevant segments did not decrease across the pages. In sum, reading skill modulates how relevant and irrelevant search results are attended to on a search page.

POSTER SESSION II, 22 READING

STUDENT UNION SQUARE HALL - 15:00- 16:30

Beginning to Characterise Children's Eye Movement Control during Reading in English: A Corpus Study

Sara Milledge, Chuanli Zang, Hazel Blythe and Simon P. Liversedge

University of Central Lancashire, United Kingdom
smilledge@uclan.ac.uk

Past research examining beginner child readers' eye movement behaviour during silent sentence reading has primarily compared when such readers move their eyes relative to skilled adult readers. The other key question regarding eye movement behaviour during reading- where readers fixate- has received much less attention. We have created a corpus in English, based on the results of three experiments (adults n = 132; children n = 132), which we are using to characterise where typically developing 8- to 9-year-old child readers move their eyes during silent sentence reading. Our systematic analyses include assessments of differences in launch site, initial landing position, refixation probability, and skipping rates (in relation to foveal and parafoveal processing of words). The results will provide insight into how child readers typically encode information about words during reading, and how visual and linguistic characteristics of words determine where the eyes move and how such behaviour differs in comparison to adult readers. We believe that such understanding will be critical to the development of models that capture and represent on-line lexical processing and eye movement control in an ecologically valid way.

POSTER SESSION II, 23 READING

STUDENT UNION SQUARE HALL - 15:00- 16:30

The Processing Strategies for Illustrated Science Reading and Chinese Academic Words with Different Semantic Transparency Among Middle-school Students: An Eye-tracking Study

Pin-Hsien Kuo and Yu-Cin Jian

National Taiwan Normal University, Taiwan
hsienkuo1023@gmail.com

This study uses eye tracking to explore the cognitive process and strategies of seventh-grade students with different reading abilities in reading illustrated scientific texts, and how readers deal with academic words with high (paraphrase) and low (transliteration) semantic transparency. Seventh-grade students (N=65) were divided into groups of reading ability through a pre-test. After reading four science texts, they answered free recall and reading comprehension questions, and finally participated in cued retrospective think aloud (CRTA). The results show that reading ability is significantly positively correlated with reading comprehension and free-recall performance. When reading transliterated words, students of all abilities have a longer gaze duration than when reading paraphrasing words, indicating the difficulty in understanding the meaning of academic words from morphemes. Furthermore, regardless of the reading ability, students use the text part as the main source of reading comprehension. However, the students realize that the form illustration has a high amount of integrated information. The eye movement retrospective think aloud data shows that high-ability students often use inference and integrated reading strategies; middle-ability students often use information extraction strategies; low-ability students often use negative reading processing methods. It is recommended that the differences in students’ reading ability should be considered.

POSTER SESSION II, 24 READING

STUDENT UNION SQUARE HALL - 15:00- 16:30

Do Chinese Deaf Readers Develop a Unique Cognitive Mechanism during Visual Word Recognition? The Effect of Oral Language Experience and Reading Ability

Zebo Lan, Nina Liu, Xiaoyuan Yuan, Meihua Guo, Guoli Yan and Valerie Benson

Faculty of Psychology, Tianjin Normal University, China
lanzeb@126.com

For most deaf readers, learning to read is a challenging task. Visual word recognition is crucial during reading. However, little is known about the cognitive mechanism of Chinese deaf readers during visual word recognition. In the present study two experiments explored the activation of orthographic, phonological, and sign language representations during Chinese word recognition. Eye movements were recorded as participants read sentences containing orthographically similar words, homophones, sign language related words or unrelated words. All deaf readers showed shorter reading times for orthographically similar words compared to unrelated words. However, when reading ability was controlled, the homophone advantage was observed only for deaf readers with more oral language experience, whereas the sign language advantage was observed only for deaf readers with less oral language experience. When oral language experience was controlled, in comparison to deaf readers with lower reading fluency levels, those with higher reading fluency levels had more stable orthographic and sign language representations. Deaf college readers with more oral language experience activate word meanings through orthographic and phonological representation, whereas deaf college readers with less oral language experience activate word meanings through orthographic and sign language representation, reflecting a unique cognitive mechanism, and, reading ability moderates this process.

POSTER SESSION II, 25 READING

STUDENT UNION SQUARE HALL - 15:00- 16:30

Eye Movements and Reading in Children Who Survived Cerebellar Tumors

Sofia Mironets, Marina Shurupova and Anna Dreneva

Dmitry Rogachev National Research Center of Pediatric Hematology, Oncology and Immunology, Neurocognitive Laboratory, Russia
sofiamironets@gmail.com

Previous investigations have demonstrated that cerebellar tumor survivors tend to have a variety of oculomotor impairments, such as hypermetria and poor gaze stability. In the current study, we aimed to evaluate the oculomotor deficits and reading parameters in children who survived cerebellar tumors. Two groups of 65 patients and 47 healthy controls, all aged 8–17, participated in the study. We analyzed the performance in several oculomotor and reading tasks. Eye movements were recorded every 1/60 s monocularly using an Arrington eye tracker. We revealed pronounced reading impairments in the patients as compared to healthy children, including longer fixation durations, greater numbers of fixations and regressive saccades, longer reading time. The patients showed gaze fixation instability and long scanpath reflecting the return of the gaze to the already counted objects. We also observed significant correlations between basic oculomotor functions and reading parameters in both groups. All these tendencies indicate that cerebellar tumor and its treatment cause oculomotor changes which can lead to disturbances in higher cognitive functions, such as reading. Our results highlight the necessity of considering these deficits in current rehabilitation protocols for pediatric cerebellar tumor survivors.

POSTER SESSION II, 26 READING

STUDENT UNION SQUARE HALL - 15:00- 16:30

Scanpath Analysis of Eye movements during Reading in Children with High Risk of Dyslexia

Anastasiya Lopukhina, Olga Parshina, Sofya Goldina, Ekaterina Iskra, Margarita Serebryakova, Vladislava Staroverova, Nina Zdorova and Olga Dragoy

National Research University Higher School of Economics, Russia
nastya.lopukhina@gmail.com

The study presents a comparison of the global reading processes estimated via scanpath analysis (von der Malsburg & Vasishth, 2011) in Russian schoolchildren with and without reading difficulties. We tested 144 children from grades 1-5 (age range 7-11; 54 girls): 72 children were at high risk of dyslexia according to the Standardized Assessment of Reading Skills (Kornev, 1997), and 72 had no reading difficulties. The children read 30 sentences while their eyes were tracked. We identified five global reading processes that all children engaged in while reading. The processes differed in fixation durations, the probability of rereading single words, and the probability of rereading entire sentences. The comparison between grades and groups revealed that children without reading difficulties progressed quickly and by grade 4 engaged in a fluent adult-like reading process. Children with high risk of dyslexia started with the beginner reading process, then engaged in the intermediate and upper-intermediate reading processes in grades 2-to-4. They reached the advanced process in grade 5 but rarely adopted the fluent reading process. In sum, the scanpath analysis revealed that children in the high-risk group and typically developing peers adopt similar reading processes, but the former group progressed much slower, with a 2-3-year delay.

POSTER SESSION II, 27 READING/L2

STUDENT UNION SQUARE HALL - 15:00- 16:30

Metacognitive Modeling Effect of Reading Illustration First for EFL Readers: A Study of Eye Movement Evidence

Ming-Yi Hsieh and Sunny S. J. Lin

Kansas State University, United States
grace.myhsieh@gmail.com

Eye-trackers have been adopted to investigate the instructional effects of modeling in science reading and reveal underlying cognitive processes. However, fewer studies investigated how to help less capable EFL readers read illustrated narratives by utilizing eye-trackers. Modeling illustration reading first has been expected to form a macrostructure before reading the text. This study explored the metacognitive modeling effect of reading illustration first with metacognitive questions as prereading guidance for EFL readers with beginning language capacity.

Participants were randomly assigned into four groups (intervention: modeled/non-modeled; lexical difficulty of article: low/high), in which illustrated narratives were provided with story structure (prologue/climax/resolution). Modeled groups were instructed to read illustrations with 5Ws questions before reading text as the model did, while non-modeled groups read in their own manners. Two-way ANOVAs were conducted to analyze posttest performance and eye measures, fixation counts (FC), dwell time (DT), and run counts (RC).

Lexical level demonstrated substantial impacts on FC, DT, and RC in both text and illustration areas of prologue and climax structures. Intervention effect shows for FC and RC in both text and illustration, but only in prologue structure. Results found that the metacognitive modeling strategy is only influential in reading the beginning of a story.

POSTER SESSION II, 28 READING/L2

STUDENT UNION SQUARE HALL - 15:00- 16:30

Lexical Access in L2 Reading: Evidence from Self-paced Reading and Eye-tracking Data

Daria Chernova, Marina Norkina and Svetlana Alexeeva

St.Petersburg State University, Russia
chernovadasha@yandex.ru

We present a study on lexical access while reading in L2. We analyze word-reading data obtained using two paradigms: self-paced reading and eye-tracking. According to (Frank et al. 2013), eye-tracking measures in L1 highly correlate with reading times in SPR for the current word and for the following word, reflecting spillover in SPR and parafoveal preview in eye-tracking.

Chinese-speaking learners of Russian (A2-B1) read the Sentence Corpus for L2 learners of Russian (90 sentences) either in SPR mode (n=65) or in an eye-tracking experiment (n=30). They read for comprehension, comprehension questions asked after 30% of sentences.

In self-paced reading data we found significant effects of word frequency, word length and predictability on reading times. In eye tracking data, we analyzed 4 measures (Frank et al. 2013): first-fixation time, first-pass time, right-bounded time and go-past time, and found strong positive correlations between all these measures and reading time of the previous (not the following) word in SPR averaged over subjects.

It can be explained by the lack of parafoveal preview and strong spillover effect in eye-tracking mode compared to SPR mode in L2 reading.

Funded by the research grant no. ID: 92566385 from St Petersburg University.

POSTER SESSION II, 29 L2 READING

STUDENT UNION SQUARE HALL - 15:00- 16:30

The Role of the Left Perceptual Span in L2 Reading: An Eye-tracking Study

Agnesa Xheladini, Leigh Fernandez and Shanley Allen

Technische Universität Kaiserslautern, Germany
xheladin@rhrk.uni-kl.de

Substantial cognitive resources are required for processing the foveal area, leaving fewer cognitive resources available for parafoveal processing. Proficient first-language (L1) readers have a perceptual span of 3-4 characters to the left and 14-15 to the right of the foveal fixation1. Given that second-language (L2) processing requires more cognitive resources2, it stands to reason that L2ers will have a smaller perceptual span than L1ers. We hypothesize L2ers will have a smaller, more symmetrical perceptual span relative to L1ers, allowing them to make use of the left span to reconfirm what they previously read.

We test the symmetry of the perceptual span using the GCMWP3 and manipulate the information available (3,6,9 characters-left/3,9,15 characters-right). Additionally, we account for the influence of English skills with German L1ers/English L2ers reading in English (n=53). L2ers benefit from an increase of window size 3-6 to the left of fixation and from 3-9 to the right of fixation, with only higher-skilled L2ers further benefiting from an increase in window size up to 15 characters to the right of the fixation. We plan to compare our data to L1ers of different ages. Overall, our data suggest that only highly skilled L2ers exhibit an L1-like asymmetric perceptual span.

POSTER SESSION II, 30 READING/L2

STUDENT UNION SQUARE HALL - 15:00- 16:30

Silent or Oral Reading in L2: An Eye-Tracking Study

Tatiana Petrova and Michael Ivanov

Saint Petersburg State University, Russia
tatianapetrova4386@gmail.com

This study aims to answer two questions: is there any effect of reading modality (oral vs. silent) on L2 text processing, and what type of reading contributes to more successful text processing and translation into L1. According to Hale et.al. (2007) reading aloud facilitates understanding of the text, despite the fact that it has a larger processing load. Fuchs et al. (2001) suggested that oral reading fluency reflects overall reading competence. It was also shown that silent reading was stronger for retelling narratives (Schimmel & Ness 2017). Translation task can be used as method for checking reading comprehension skills (Karimnia, 2014). In a two-group experimental design, native speakers of Russian (N=20, B2-C1 level of English) read either orally or silently two English texts, estimated the subjective difficulty of each text, and then translated them into Russian. Both texts were of the same length, topic and level of readability (checked via http://readable.com). The eye movements of the participants were recorded (EyeLink 1000 Plus by SR Research). We measured the total reading time (TRT), total fixations count (TFC), average fixation duration (AFD), and regressions count (RC). The quality of the translations were assessed by Gilmullina's test (2016) of quantitative analysis. Mann-Whitney U test showed that reading was significantly slower (RT: p=0,008; TFC: p=0,027; AFD: p=0,036) when subjects read text aloud, as opposed to silently. No interaction was found between quality of the translations and subjective difficulty of the stimuli. So oral reading slows down text processing without any contribution to comprehension.

Supported by the research grant no. ID: 92566385 from St Petersburg University

POSTER SESSION II, 31 REAL-WORLD

STUDENT UNION SQUARE HALL - 15:00- 16:30

A Two-tier Taxonomy of Gaze Behaviours for Free-moving Participants

Mark Shovman, Anna Morozov and Ksenia Burgart

Eyeviation Ltd., Israel
mark.shovman@gmail.com

A Gaze Event Detector (GED) is an algorithmic component that parses a time series of eye positions and directions into meaningful gaze events (aka oculomotor behaviours). The best-known gaze events are fixations and saccades. There are also smooth pursuits (SPs), events caused by vestibulo-ocular and opto-kinetic reflexes (VOR and OKR), vergence shifts, and more.

Many GED algorithms have been developed in the last 50 years; most popular are versions of either IVT or IDT. Most of these are suited to the experimental paradigms with the participant sitting in front of a flat stimulus display. Thus they often equate gaze movement with eye movement, disregard head movement, cannot account for vergence changes,etc.

Nowadays, eyetracking technology rapidly becomes a ubiquitous component of most XR devices. One barrier for an adoption of gaze analysis in XR is a lack of GED algorithms that process coordinated eye-head-body gaze movements in complex 3D stimulus scenes.

We propose a novel two-tier taxonomy of gaze behaviours, that combines atomic eye-head-body movements into meaningful gaze behaviours, such as: focusing on a static target; following a moving target; shifting attention; internal thinking; etc.; and present a preliminary algorithmic implementation of it.

POSTER SESSION II, 32 REAL-WORLD

STUDENT UNION SQUARE HALL - 15:00- 16:30

Fixation Sequences When Walking Up and Down Stairs in Daily Life

Andrea Ghiani, Joost G. Driessen, Liz R. Van Hout and Eli Brenner

Vrije Universiteit Amsterdam, Netherlands
a.ghiani@vu.nl

In our daily life there are many situations in which it is not directly evident where gaze should be directed: not all tasks require constant visual guidance and there may be reasons to look elsewhere. Walking up or down a staircase is a good example of such a situation. We investigated participants' gaze behaviour when walking on stairs as part of a navigation task in their own house. Participants did not know that stairs were the focus of investigation. We analysed the order in which participants fixated the steps, confirming earlier reports that people often looked at each step sequentially. However, we found that participants also often made fixations back to steps that had already been fixated and that they regularly skipped looking at several steps to fixate further ahead. The main difference between ascending and descending the staircase was found when approaching the first step: when descending participants looked extensively at the beginning of the staircase, whereas when ascending they did not. This study shows that focussing on sequences of fixations is useful when investigating stair climbing with a variety of staircases in different environments.

POSTER SESSION II, 33 REAL-WORLD

STUDENT UNION SQUARE HALL - 15:00- 16:30

Investigating the Effects of Task and Body Movement on the Generalizability of Scene-viewing Experiments

Daniel Backhaus and Ralf Engbert

University of Potsdam, Germany
backhaus@uni-potsdam.de

Scene-viewing experiments conducted in the laboratory attempt to understand human gaze behavior and to generalize findings. This generalizability has often been questioned. Recent advances in eye tracking technology allow for experiments outside the laboratory with a high degree of mobility and enable participants to have more freedom of movement.

In the current study we investigate eye movements using mobile eye tracking devices, but remain in the laboratory in order to investigate how distinct experimental conditions affect eye movements. Here we present the effects of A) the given scene viewing task and, B) the possibility of head movements, on scan path statistics.

The given task clearly affects both temporal and spatial gaze parameters. We find differences in behavior even for apparently minor changes in task instructions such as free viewing and guessing tasks with only little task constraints. In our experiments, the subjects' freedom of movement hardly affects temporal gaze parameters but noticeably affects spatial parameters.

Our results are consistent with the view that laboratory factors such as a chin rest do not cause artefacts that limit the generalizability of laboratory findings. However, the absence of a task, or a free viewing task, significantly affects gaze behavior.

POSTER SESSION II, 34 REAL-WORLD

STUDENT UNION SQUARE HALL - 15:00- 16:30

Automated Discrimination of Stable and Non-stable Gaze Events in Dynamic Natural Conditions

Ashkan Nejad, Eva Postuma, Gera de Haan, Joost Heutink and Frans Cornelissen

Royal Dutch Visio, Netherlands
a.nejad@rug.nl

Introduction

It is challenging for people with visual field defects to perform daily tasks that rely on having a good visual overview. For helping people with such a condition, an essential step is to quantify their scanning behavior. However, there are no accurate gaze event detectors suitable for use in dynamic natural conditions, limiting research in such settings. We aim to design a gaze-event detector for free head and body movements conditions.

Methods and Results

Our event detector interprets environmental movements using optic flow estimation. Additionally, it employs point tracking on surrounding patches of gaze location to analyze the gaze path. By combining this information, the detector can discriminate between and describe and visualize stabilizing and non-stabilizing gaze events.

We tested our method on samples recorded using a Pupil Invisible in 15 participants who conducted a series of predefined activities, including simultaneously moving and following an object of interest. The method successfully discriminated between our two classes of events and is being improved.

Conclusions

We conclude that our gaze event detection method is suitable for examining visual scanning behavior in dynamic natural settings. Researchers can benefit from this method in investigating the scanning behavior in people with visual field defects.

POSTER SESSION II, 35 SOCIAL COGNITION

STUDENT UNION SQUARE HALL - 15:00- 16:30

Investigating Face Perception during Free-viewing in a Naturalistic Virtual Environment

Debora Nolte, Marc Vidal De Palol and Peter König

University of Osnabrück, Germany
debnolte@uos.de

Face perception is commonly investigated in standardized lab settings with high experimental control during which eye movements are generally restricted and the fixated stimuli are predetermined. While faces are considered prevalent and important stimuli (e.g., Wheatley et al., 2011), little research has explored the perception of faces in naturalistic settings. The current study combines high experimental control with natural viewing and movement behavior by investigating face perception in a virtual environment. Our virtual city consists of houses, various background stimuli, and notably static and moving pedestrians. Participants freely explore the virtual scene while eye-tracking and EEG data are recorded. We investigate participants' distribution, duration, and distance of gaze events on faces and the participant's movement path. Preliminary results indicate large between-subject differences in the number of gazes on the bodies and faces of pedestrians. Additionally, big differences in the subjects' movement patterns can be observed. The findings of this study will provide insights into face perception in a naturalistic virtual environment.

Wheatley, T., Weinberg, A., Looser, C., Moran, T., & Hajcak, G. (2011). Mind perception: Real but not artificial faces sustain neural activity beyond the N170/VPP. PloS one, 6(3), e17960.

POSTER SESSION II, 36 SOCIAL COGNITION

STUDENT UNION SQUARE HALL - 15:00- 16:30

Looking for Speaking: What Determines Language-specific Expressions in Motion Event Descriptions

Yuko Yoshinari

Gifu University, Japan
yyoshi@gifu-u.ac.jp

This cross-linguistic experimental study examines the relationship between event construal and linguistic expressions using the eye-tracking method. We focus on descriptions of motion events elicited from a picture book by English, Italian and Japanese speakers. Because these languages are grouped into two based on the typology of motion expression.

Slobin (1991, 1996) presented a modified form of linguistic relativity: “thinking for speaking”. He claimed that the language we learn shapes the way we perceive reality based on the linguistic experiment of various languages. Our study replicated Slobin’s experimental method and investigated the speakers’ eye movement when describing pictures. We examined how the speaker’s construal of an event tends to reflect in language expressions used in describing the event.

We have obtained the data from 9 participants from each language and analysed a scene where “a boy fell off the cliff into the pond”. We found individual tendencies in each language, what kind of path components (DOWN, FROM, INTO) are expressed frequently, and how eye movements, such as fixation duration, correspond to them. A comparison of patterns of eye movement and language expressions revealed the relation between perception and speech production to be either universal or individual.

POSTER SESSION II, 37 SOCIAL COGNITION

STUDENT UNION SQUARE HALL - 15:00- 16:30

Gaze Aversions Serve as Social Signals Conveying the Performer's Cognitive State

Amit Zehngut, Dekel Abeles and Shlomit Yuval-Greenberg

Tel-Aviv University, Israel
amitz@mail.tau.ac.il

When engaged in effortful cognitive processing, we often avert our gaze to the periphery. Studies explained this phenomenon as an attentional mechanism of distraction avoidance. Here we propose that, in addition to its contribution to attentional processes, gaze aversion also serves as a signal in social interaction, conveying information regarding the performer's cognitive state. As a first step in investigating this hypothesis, we examined how well perceivers infer other people's cognitive states in social interaction, and how this ability depends on eye movements. In two experiments, participants (N=40 each) watched short (5s) muted videos depicting individuals during social interactions. Results of the first experiment showed that participants succeeded in identifying when other individuals were engaged in cognitive processing, relative to listening or tapping their feet. Furthermore, participants were more likely to correctly identify an individual as engaged in cognitive processing, when this individual was shifting their gaze. In a second experiment, we found that when individuals performed gaze aversions while they were engaged in an effortful cognitive task, they were rated by others as more concentrated in the task, and more likely to provide a correct response. Together, our findings suggest that effortful cognitive processing is communicated via gaze aversions.

POSTER SESSION II, 38 SOCIAL COGNITION

STUDENT UNION SQUARE HALL - 15:00- 16:30

Gaze Aversion in Human-Robot Interaction: Case Studies in Physical and Virtual Settings

Cengiz Acarturk, Bartlomiej Sniezynski, Bipin Indurkhya, Piotr Nawrocki and Sinan Kalkan

Jagiellonian University; Middle East Technical University, Poland
cengiz.acarturk@uj.edu.pl

Human Robot Interaction (HRI) is an interdisciplinary research domain that focuses on verbal and nonverbal communication between physical or virtual robotic agents and human interlocutors. As in human-human dyads, gestures and gaze have been primary nonverbal communication modalities in HRI. This study presents our findings from an HRI framework, which has been designed and implemented to support developing applications that employ gaze and other nonverbal modalities for interacting with people. In experimental investigations, we investigated gaze contact and aversion in communication between humans, between humans and physical robots, and between humans and virtual agents. We have been developing the framework to model gaze-mediated communication between two agents, where an agent can be a robot or an avatar, to allow the agent to interact naturally and intuitively with a human user. This study aims at presenting a snapshot of the state of the art in HRI, the experience gained from experimental investigations, and the items for future work.

POSTER SESSION II, 39 SOCIAL COGNITION

STUDENT UNION SQUARE HALL - 15:00- 16:30

Semantics of g`aze: Deciphering the Meaning of a Listener's Gaze Direction, Gaze Position Changes, and Blink Frequency

Lynn Huestegge, Eva Landmann, Christina Breil and Anne Böckler-Raettig

Würzburg University, Germany
lynn.huestegge@uni-wuerzburg.de

In the present study, we developed a novel methodology to understand the semantics of perceived gaze patterns. Using a qualitative approach, we presented participants with videos showing a person that is involved in listening to brief (neutral vs. emotional) stories. The eye movements of the listening person were systematically manipulated regarding gaze direction, changes of gaze position, and blink frequency. After each presentation of a subset of these videos, participants were asked how they would verbally characterize the different gaze patterns. By applying semantic categorization procedures, we were able to link particular gaze patterns to distinct semantic categories (e.g., attentiveness, nervousness, empathy etc.). The resulting exploratory findings are subsequently submitted to rigorous experimental testing. Limitations in the generalizability of the present findings to other situational and social contexts will be critically discussed.

SYMPOSIUM: EYE MOVEMENTS IN MEMORY PROCESSES: BETWEEN WORKING MEMORY AND LONG-TERM MEMORY

BENNETT LECTURE THEATRE 1 - 10:00- 00:00

Chair: Shlomit Yuval-Greenberg

Tel Aviv University, Israel
shlomitgr@tau.ac.il

Organiser: Shlomit Yuval-Greenberg (Tel Aviv University)

From the day we are born and until our death we are constantly engaged in exploration of our ever-changing environment. In the continuous process of visual exploration, our eye movements play a critical role as they repeatedly shift the center of gaze towards locations of interest. However, eye movements are not only driven by the physical presence of stimulation, but also by internal representations of stimuli that are not physically present. Such internally-driven eye movements are thought to play a key role in memory processes at both shorter- and longer-term time scales. This symposium will bring together scholars studying different types of eye movements, including saccades, microsaccades and smooth pursuit, in both working memory and episodic long-term memory tasks. The first three talks will focus on working memory and discuss how different types of eye movements can be used as windows into working memory processes. The next three talks will focus on episodic memory and examine the dynamics of gaze during encoding and retrieval and their neural correlates. The goal of this symposium is to lead to a discussion comparing the various types of eye movements and their roles in short and long-term memory.

SYMPOSIUM: EYE MOVEMENTS IN MEMORY PROCESSES: BETWEEN WORKING MEMORY AND LONG-TERM MEMORY

BENNETT LECTURE THEATRE 1 - 10:00- 10:20

Utilising Directional Microsaccade Biases as a 'Tool' to Track Selective Attention inside Working Memory in Time and Space

Freek van Ede

Vrije Universiteit Amsterdam, Netherlands
freek.van.ede@vu.nl

Selective attention can not only be directed to external sensations, but also to internal representations within working memory. We have recently uncovered how such internally directed selective attention is associated with directional biases in microsaccades – extending the role of the oculomotor system to internal orienting of attention. In my talk, I will show how we have started to utilise directional biases in microsaccades as a novel tool to track internal attention along 3 dimensions: to track (1) whether internal attention is deployed, (2) when it is deployed, and (3) where it is deployed. Doing so, I will illustrate how the study of microsaccades can be used to uncover new insights into the mechanisms of internally directed selective attention in dynamic and immersive settings.

SYMPOSIUM: EYE MOVEMENTS IN MEMORY PROCESSES: BETWEEN WORKING MEMORY AND LONG-TERM MEMORY

BENNETT LECTURE THEATRE 1 - 10:20- 10:40

What the Variations in Saccade Metrics and Visual Memory Across the Visual Field Tell About Saccadic Selection in Visual Working Memory

Sven Ohl

Humboldt-Universität zu Berlin, Germany
sven.ohl@hu-berlin.de

Saccades select content that is currently maintained in visual working memory—resulting in better memory at locations that are congruent with the saccade target than at incongruent locations. Using a data set of nine experiments (including eight published experiments) with more than 100k trials, we further substantiate the claim of a fundamental saccadic selection mechanism in memory by assessing whether saccadic selection is effective in all participants and at all tested locations.

In the experiments, we briefly presented arrays of oriented stimuli at eight possible locations that were arranged on an imaginary circle around central fixation. Several hundred milliseconds later, a movement cue prompted saccades to one of these locations. Next, participants were prompted to report the orientation (clockwise vs. counterclockwise) of the memory item at a randomly highlighted location.

Using Bayesian hierarchical models, we observed saccadic selection in memory at all tested locations. Individual differences in saccadic selection were compatible with a population-wide model of effective saccadic selection. Moreover, saccade metrics and visual working memory varied strongly across the visual field. Trial-by-trial variations in saccade metrics were associated with memory performance, providing additional evidence for a bi-directional link between the oculomotor system and visual working memory.

SYMPOSIUM: EYE MOVEMENTS IN MEMORY PROCESSES: BETWEEN WORKING MEMORY AND LONG-TERM MEMORY

BENNETT LECTURE THEATRE 1 - 10:40- 11:00

Eye Movements as a Window into Time-dependent Memory Processes

Miriam Spering, Annick Langlois, Philipp Kreyenmeier, Lisa Kroell, Anna Heuer and Martin Rolfs

University of British Columbia, Vancouver, Canada
miriam.spering@ubc.ca

An important capacity of human memory is to help us perceive, recognize, and keep track of visual objects and events. This ability enables us to perceive a stable visual world despite dynamic changes caused by object motion or by body movement. To achieve perceptual stability, our memory system must create an episodic representation of successive states of objects and events in our environment. How visual information is integrated across space and time to form such representations is not well understood. Here I will present a series of experiments that utilizes smooth pursuit eye movements as a sensitive probe into time-dependent memory processes. Observers tracked a cluster of moving, coloured objects that temporarily disappeared behind an occluder. Upon reappearance, object color was either the same, new, or switched between two objects. Observers' reaction time and performance were best when the display was the same, and worst when the color switched. Smooth pursuit velocity at object reappearance immediately reflected perceptual judgments, indicating that eye movements can indicate processes of memory formation at high temporal resolution.

SYMPOSIUM: EYE MOVEMENTS IN MEMORY PROCESSES: BETWEEN WORKING MEMORY AND LONG-TERM MEMORY

BENNETT LECTURE THEATRE 1 - 11:00- 11:20

Gaze Behavior Supports Episodic Memory: insights from Electrophysiological Data

Mikael Johansson, Inês Bramão, Andrey Nikolaev and Roger Johansson

Lund University, Sweden
mikael.johansson@psy.lu.se

Episodic memory allows us to re-experience past events in exquisite detail in the “mind’s eye”. Previous research suggests that eye movements may play a functional role during encoding by binding together event details into a coherent memory representation and during retrieval by facilitating the reconstruction of the past event. However, less is known about the interplay between gaze and memory mechanisms as encoding and retrieval unfold in time. The talk will describe two studies that combined eye-tracking and electrophysiological recordings of brain activity (EEG) to capture the temporal dynamics of gaze-memory interactions. The first part concerns encoding and the neural mechanisms that subserve episodic memory formation across saccades to event elements during free viewing. The second part examines retrieval and the neural correlates of the looking-at-nothing effect, i.e., superior performance when gaze location overlaps between encoding and retrieval. The results support claims of a functional role of eye movements in memory and reveal the time course of memory-related oscillatory neural activity. : i) synchronization in the theta band during encoding predicts subsequent relational memory for event elements, ii) desynchronization in the alpha/beta band during retrieval covaries with facilitated episodic remembering at congruent gaze locations.

SYMPOSIUM: EYE MOVEMENTS IN MEMORY PROCESSES: BETWEEN WORKING MEMORY AND LONG-TERM MEMORY

BENNETT LECTURE THEATRE 1 - 11:20- 11:40

The Intersection of Memory and Active Vision in Aging

Jennifer Ryan, Jordana Wynn, Kelly Shen and Zhong Xu Liu

Rotman Research Institute, Canada
jryan@research.baycrest.org

The oculomotor and hippocampal memory systems interact in a reciprocal manner. Eye movements accumulate information from the visual world, contributing to the formation or updating of coherent memory representations. Conversely, memory influences ongoing viewing by increasing the efficiency of active vision. Eye movements contribute to memory retrieval by reconstructing the rich, vivid, spatiotemporal details from memory (gaze reinstatement). However, the interactions between the oculomotor and memory systems are altered in aging. In older adults, neural activity in the hippocampus is not modulated by gaze fixations to the same extent as observed in younger adults, despite the fact that older adults typically enact more gaze fixations than younger adults. Older adults also exhibit less unique patterns across different stimuli, and across repeated viewings of the same stimulus. Together, these findings suggest that the memory representations formed by older adults may be less complete and/or less distinct than those of younger adults. Consequently, even when older adults engage in gaze reinstatement at retrieval, such reinstatement does not necessarily support accurate memory performance, and instead, may explain memory errors that are observed with aging. This work highlights how age-related changes in the hippocampal memory system may have a broad impact on active vision.

SYMPOSIUM: EYE MOVEMENTS IN MEMORY PROCESSES: BETWEEN WORKING MEMORY AND LONG-TERM MEMORY

BENNETT LECTURE THEATRE 1 - 11:40- 12:00

What Makes Eye Movements a Memory Retrieval Cue?

Shlomit Yuval-Greenberg and Keren Taub

Tel-Aviv University, Israel
shlomitgr@tau.ac.il

We normally move our eyes when we wish to focus our gaze on objects or locations of interest. However, there are also times when we do so even in the absence of visual stimulation. Sometimes we shift our gaze towards places where we remember having seen something. This behavior was previously suggested to reflect the role of eye-movements as retrieval cues, but it is hitherto unknown what are the factors contributing to this role. In a series of studies, we examined this question by contrasting two hypotheses. The motor hypothesis states that a crucial factor contributing to the role of eye-movements as retrieval cues is the match in the pattern of muscle contraction between encoding and retrieval. The visual hypothesis states that the crucial factor contributing to memory, is the encoding-retrieval match of the visual image that falls on the retina following an eye-movement. Our findings show that both the visual and the motor factors of eye movements may contribute to memory performance as retrieval cues, depending on the task. Furthermore, we find that people vary in their ability to gain from eye-movement-related cues, and that the gain from visual cues is tightly linked to the gain from motor cues.

CHINESE READING

BENNETT LECTURE THEATRE 2 - 10:00- 10:20

Word Length and Frequency in Chinese Reading: Evidence from Eye Movements

Ying Fu, Simon P. Liversedge, Maleeha Moosa, Xuejun Bai and Chuanli Zang

Tianjin Normal University, China
yfu11@uclan.ac.uk

Previous studies on alphabetic languages showed mixed results regarding how word length and frequency jointly affect reading. A probable reason is that the relationship between word length and frequency varies across the (wide) range of word lengths in alphabetic languages. Unlike alphabetic languages, almost all Chinese words are one or two characters long, resulting in much less such variability. How, then, does word length interact with frequency during Chinese reading? We orthogonally manipulated word frequency (high or low) and word length (a one-character word, a two-character word with equivalent total stroke number, or a two-character word with its first character stroke number equivalent to that of the one-character word). Uniquely, this represents a manipulation of length without visual complexity confounds. The results showed reliable effects of word length on skipping, landing positions, gaze and total fixations and robust word frequency effects on reading times. There were no reliable interactions on any measures. These effects appeared regardless of whether stroke number for the overall word, or its first character was matched. The results suggest character level processing in word identification and independent word length and frequency influences on eye guidance during Chinese reading.

CHINESE READING

BENNETT LECTURE THEATRE 2 - 10:20- 10:40

The Role of Radicals during Parafoveal Processing of Chinese Characters

Federica Degno, Lixin Wei, Simon P. Liversedge, Manman Zhang, Mengsi Wang and Chuanli Zang

University of Bournemouth, United Kingdom
fdegno@bournemouth.ac.uk

Although character-level phonological and semantic preview benefits have been observed during Chinese reading, less clear is the role of phonological and semantic coding at the sub- lexical level. We conducted two eye movement experiments and manipulated the parafoveal preview of a two-radical Chinese character using the boundary paradigm to examine whether parafoveal processing of phonetic (P) and semantic (S) radicals is based on their function or position. In Experiment 1, the character had either a left-to-right (SP) or a right-to-left (PS) structure and was presented in the parafovea with one, both, or none of the radicals being masked by a meaningless radical. In Experiment 2, the character had a SP structure, and both identity and position of the radicals were manipulated, such that an identical or meaningless mask was presented in the parafovea, and one, both, or none of the radicals were presented in the correct position. The data from both experiments suggest that the phonetic radical is especially important during parafoveal processing, and its disruption is more costly to processing than disruption of the semantic radical. We will discuss our findings in relation to psychological models of Chinese word recognition and eye movement control during reading.

CHINESE READING

BENNETT LECTURE THEATRE 2 - 10:40- 11:00

Reading Classical Chinese fables with Implicit Moral Point: Eye-movement Evidences of Lexical Difficulty, Paragraph Focus and Order Effects

Zheng-Hong Guan and Sunny S. J. Lin

National Yang Ming Chiao Tung University, Taiwan
a0935220867@gmail.com

Fables consist of the story and moral point and aim to convey moral messages via the story. Classical Chinese fables are very difficult; not only because its terseness style but also the ancient moral lessen might be less explicit for the modern readers. Undergraduates were asked to read two Classical Chinese fables (lexical easy vs. difficult), each consisting of two paragraphs (story and moral point). Two issues were examined: (1) Which paragraph did the reader focus more when reading fables with implicit moral points? (2) Whether a fable with different paragraph orders or various lexical difficulty would affect comprehension outcomes and eye-movement measures? The results showed when reading lexical easy or story-first order fable, readers had better comprehension. For eye-movement measures, the main effect shows implicit moral point gained more attention than story. However, the effect was moderated by order and lexical difficulty. When reading moral point-first fable, readers spent more first-pass reading time and rereading time on moral point than story, especially for lexical difficult fable. When reading story-first fables with easy or difficult lexicon, there is no significant different total reading time between moral point and story, implying that stories helped readers to successfully achieve decoding and integration.

CHINESE READING

BENNETT LECTURE THEATRE 2 - 11:00- 11:20

Foveal and Parafoveal Processing of Chinese Four-character Idioms and Phrases in Reading

Chuanli Zang, Shuangshuang Wang, Xuejun Bai, Guoli Yan and Simon P. Liversedge

University of Central Lancashire, United Kingdom
czang@uclan.ac.uk

Research has demonstrated that Chinese three-character idioms are represented and processed foveally and parafoveally as Multi-Constituent Units (MCUs, see Zang et al., 2021). Chinese four-character idioms and frequently used four-character phrases extend further into the parafoveal region during natural reading. Are they also processed as MCUs? Using the boundary paradigm (Rayner, 1975), we manipulated the preview of the first, and the second, two character constituents of four-character idioms (Experiment 1) and frequently used phrases (Experiment 2). Previews were identities or pseudocharacters. Both experiments produced greater preview benefit for the second constituent when the first constituent was an identity preview compared with when it was a pseudocharacter preview suggesting that the presence of the first constituent licensed parafoveal processing of the second. In a third experiment, we compared preview effects in frequently used four-character phrases (judged as single four-character words), ambiguous four-character strings (judged equally often as single four-character words and as two two-character words), and strings that were unambiguously two two-character words. Preview effects for the second constituent were more pronounced for the former than the latter two strings. Together these results indicate that four-character idioms and frequently used phrases are processed foveally and parafoveally as single, unified lexical representations.

CHINESE READING

BENNETT LECTURE THEATRE 2 - 11:20- 11:40

Flexible Parafoveal Encoding of Character Order Supports Word Predictability effects in Chinese for Both Young and Older Adult Readers

Min Chang, Kuo Zhang, Lisha Hao, Kevin B Paterson, Kayleigh L. Warrington and Jingxin Wang

Tianjin Normal University, China
tjsfchangmin@126.com

Eye-movement studies in Chinese show both that (a) character order is encoded flexibly during parafoveal processing, and (b) target word predictability can influence this early stage of a word’s processing. However, it is unclear whether these effects change in older age. Accordingly, we investigated this issue in an eye movement experiment using the boundary paradigm (Rayner, 1975) with 36 young (18-30 years) and 36 older (65-75 years) adults. These participants read sentences containing two-character target words with high or low contextual predictability. Prior to the reader's gaze crossing an invisible boundary, each target word was shown normally (i.e., a valid preview) or with its two characters either transposed or replaced by unrelated characters to create invalid nonword previews, which reverted to the target word as soon as the reader's gaze crossed the invisible boundary. The results replicated previous findings of a transposed-character effect (larger preview benefits for transposed-character than unrelated previews), and a word predictability effect (shorter reading times for words with high than low predictability) following valid and transposed-character previews, but not unrelated previews. We take these findings to show that both flexible character order processing and an early influence of contextual predictability is preserved in older Chinese readers.

CHINESE READING

BENNETT LECTURE THEATRE 2 - 11:40- 12:00

Word Length Effect in Developing Chinese Readers during Sentence Reading

Nina Liu, Xiyuan Li, Yongsheng Wang, Guoli Yan, Ascensión Pagán and Kevin B. Paterson

Tianjin Normal University, China
lnnair@163.com

Word length has a fundamental role in determining where and when the eyes move during reading in both Chinese and alphabetic languages. However, surprising little is known about how the influence of word length develops in Chinese reading, since more difficult to segment words from unspaced text to obtain word length information for children readers. Accordingly, to gain insight into its use during reading development, we examined the effect of word length of a specific target word (one-, two-, or three-character word) in sentences on the eye movements of developing Chinese readers (children in 3rd and 5th grade of primary school and adults). The findings show that longer words were fixated for longer and less likely to be skipped and the saccade targeting was closer to the right side of the word than that of short words for both children and adults. More importantly, the effects of word length decreased with age on fixation times, but reversed developmental patterns on saccade targeting; and developing readers with stronger word knowledge were more skillful at using word length to modulate both fixation times and saccade targeting. We discuss implications for models of eye movement control and developing reading ability in Chinese children.

SPECIAL POPULATIONS

BENNETT LECTURE THEATRE 2 - 13:00- 13:20

Activation of ASL Signs during Sentence Reading for Deaf Readers: Evidence from Eye-tracking

Emily Saunders, Jonathan Mirault and Karen Emmorey

San Diego State University, United States
ecsaunders@sdsu.edu

Prior research has established that bilinguals activate both of their languages as they process written words, regardless of orthographic system or modality (i.e., spoken or signed). These effects have been documented in single-word reading paradigms; however, less is known about co-activation when reading in context. The present study used eye-tracking to determine whether deaf bilingual readers activate American Sign Language (ASL) translations as they read English sentences. Stimuli sentences contained a target and one of two possible primes: a related prime which shares phonological parameters (location, handshape, or movement) with the target when translated into ASL, or an unrelated prime which has no form overlap in English or ASL. Eye-tracking measures from 23 deaf native signers revealed that first fixation durations and total gaze durations on target words were shorter when the target was preceded by primes with shared parameters in their ASL translations. These data suggest that ASL phonology is activated when deaf signers read English, facilitating access to related words even without phonological relationships in the written language. These effects were not moderated by reading skill, suggesting that degree of ASL activation does not decrease with increased proficiency in English, in contrast to previous studies.

SPECIAL POPULATIONS

BENNETT LECTURE THEATRE 2 - 13:20- 13:40

Skilled, Efficient Reading in Deaf Child Signers: A Small-scale Eye-tracking Study

Frances Cooley and Elizabeth Schotter

University of South Florida, United States
cooley.frances@gmail.com

Deaf adult signers who are skilled readers read more efficiently than hearing non-signers, resulting in shorter fixations, longer saccades, and fewer fixations and regressions [1,2,3,4]. The only study investigating reading behavior in native-signing deaf children used an unnatural moving window paradigm, but suggests that reading-age matched deaf signers make fewer, longer fixations compared to hearing children1. We report reading behavior data from 10 deaf native signers and 14 hearing non-signers ages 10-13 who completed a passive reading paradigm on an EyeLink 1000. Unlike the previous study, while hearing readers read above age-expected levels, and deaf readers performed at age-expected levels. Regression analyses predicting the number and duration of fixations based on reading fluency5 and participant group demonstrated that, on average, deaf children made fewer ($p = 0.019$*) and shorter fixations ($p = 0.029$*) than hearing children. In addition, for deaf readers fixation durations decreased with increase reading fluency ($p = 0.03$*; Figure 1), but the fixation durations of hearing readers did not differ based on reading fluency. These results confirm that deaf children make fewer and longer fixations, but also suggest that their eye movements reflect a transition to skilled, efficient reading during early middle school.

1. Bélanger, N. N., Lee, M., & Schotter, E. R. (2018). Young skilled deaf readers have an enhanced perceptual span in reading. Quarterly Journal of Experimental Psychology, 71 (1), 291-301 .

2. Bélanger, N. & Rayner, K. (2015). What eye movements reveal about deaf readers. Association for Psychological Science, 24 (3), 220-226.

3. Costello, B., Caffarra, S., Fariña, N., Andoni Duñabeitia, J., & Carreiras, M. (2021). Reading without phonology: ERP evidence from skilled deaf readers of Spanish. Scientific Reports, 11 (1), 1 - 11

4. Traxler, J. M., Banh, T., Craft, M. M., Winsler, K., Brothers, T. A., Hoversten, L. J., Piñar, P., & Corina, D. P. (2021). Word skipping in deaf and hearing bilinguals: Cognitive control over eye movements remains with increased perceptual span. Applied Psycholinguistics, 2021, 1-30. doi:10.1017/S0142716420000740

5. Woodcock, R. W., McGrew, K. S., & Mather, N. (2001). Woodcock-Johnson III Tests of Cognitive Abilities. Rolling Meadows, IL: Riverside.

SPECIAL POPULATIONS

BENNETT LECTURE THEATRE 2 - 13:40- 14:00

Gender and the Formation of Co-reference Links during Reading in Autism

Philippa Howard, Valerie Benson and Simon P. Liversedge

University of Bristol, United Kingdom
philippa.howard@bristol.ac.uk

Autistic people often experience reading comprehension challenges. This experiment assesses competing theoretical predictions of how co-reference processing, which is often essential for comprehension, might differ for autistic and non-autistic people. This study examines 1) the efficiency of co-reference link formation, 2) whether referential processing is modulated by the type of information that informs a link (lexical vs. world knowledge), and 3) whether co-reference links are maintained in the mental representation of text. Data from (at least) 24 autistic and 24 non-autistic participants will be presented. Both participant groups completed a battery of cognitive assessments and read passages of text as their eye movements were recorded. Texts were manipulated to include occupation nouns with a definitional gender (e.g., fireman) or a stereotypical gender (e.g., firefighter), that were followed by a target reflexive pronoun that either matched (e.g., himself), or mismatched (e.g., herself) the definitional/stereotypical gender. Preliminary gaze duration and total time data for the target pronoun (currently 19 autistic, 11 non-autistic participants) indicate that all participants experienced disruption to reading when a gender mismatch occurs, but that the time course of these effects may differ between groups. Full findings will be presented and discussed in relation to cognitive theories of autism.

VISUO-MOTOR

BENNETT LECTURE THEATRE 8 - 10:00- 10:20

Neglect-like Visual Exploration by Gaze-contigent Manipulation of Scenes

Andreas Sprenger, Lisa Kunkel Genannt Bode, Christoph Helmchen and Bjoern Machner

University of Luebeck, Dept. of Neurology, Germany
andreas.sprenger@neuro.uni-luebeck.de

Selective spatial attention is a crucial cognitive process that guides us to the behaviorally relevant objects in a complex visual world by using exploratory eye movements. The spatial location of objects, their (bottom-up) saliency and (top-down) relevance is assumed to be encoded in one “attentional priority map” in the brain, using different egocentric (eye-, head- and trunk-centered) spatial reference frames. In patients with hemispatial neglect, this map is supposed to be imbalanced, leading to a spatially biased exploration of the visual environment. As a proof of concept, we altered the visual saliency (and thereby attentional priority) of objects in a naturalistic scene along a left-right spatial gradient and investigated whether this can induce a bias in the exploratory eye movements of healthy humans (N = 28; all right-handed; mean age: 23 years, range 19–48). We developed a computerized mask, using high-end “gazecontingent display (GCD)” technology, that immediately and continuously reduced the saliency of objects on the left—“left” with respect to the head (body-centered) and the current position on the retina (eye-centered). In both experimental conditions, task-free viewing and goal-driven visual search, this modification induced a mild but significant bias in visual exploration similar to hemispatial neglect. Accordingly, global eye movement parameters changed (reduced number and increased duration of fixations) and the spatial distribution of fixations indicated an attentional bias towards the right (rightward shift of first orienting, fixations favoring the scene’s outmost right over left). Our results support the concept of an attentional priority map in the brain as an interface between perception and behavior and as one pathophysiological ground of hemispatial neglect. Consequently gaze-contingent manipulation of scenes might be used for diagnostic and therapeutical purposes.

VISUO-MOTOR

BENNETT LECTURE THEATRE 8 - 10:20- 10:40

Familiar Objects Benefit More from Transsaccadic Feature Predictions

Nedim Goktepe and Alexander C. Schütz

Philipps-Universität Marburg, Germany
goektepe@staff.uni-marburg.de

Transsaccadic predictions of how object appearance changes at different eccentricities can be made on the basis of object-specific peripheral-foveal associations. However, it is unclear if transsaccadic predictions are limited to familiar objects where these associations can be acquired through experience. In two experiments, we tested whether there is an advantage for familiar compared to novel objects in peripheral-foveal matching and transsaccadic change detection tasks.

In both experiments, observers unknowingly familiarized with a subset of objects. Subsequently, observers in the first experiment completed a peripheral-foveal matching task, where the foveal object matching to the peripheral probe had to be selected. Observers in the second experiment performed a transsaccadic change detection task, where they had to detect whether a peripheral target was or was not exchanged immediately after the saccade or after a 300 ms blank period.

In both experiments, familiar objects had an advantage over novel objects. In the first experiment, we found that the familiarity effect depended on foveal-peripheral predictions. In the second experiment, we showed that peripheral-foveal associations explained the advantage of familiar objects. A postsaccadic blank improved the change detection overall but more for familiar objects. In conclusion, we found that transsaccadic predictions are facilitated for familiar objects.

VISUO-MOTOR

BENNETT LECTURE THEATRE 8 - 10:40- 11:00

This Vortex Cannot be Pursued

Krischan Koerfer, Tamara Watson and Markus Lappe

University of Muenster, Germany
krischan.koerfer@uni-muenster.de

Non-rigid motion of water, clouds, smoke, fire etc. is omnipresent in our world and its visual perception is intermingled with eye movements. Here, we investigated the pursuit of non-rigid motion by presenting a vortex motion pattern in a random dot distribution. The vortex moved across the screen, independent of the first-order motion within it. We asked 15 participants to pursue this vortex. We found pursuit gain was almost zero and the frequent catch-up saccades were too short, landing where the vortex had been during saccade planning. Furthermore, participants reported that the vortex appeared to jump during pursuit. In contrast, the vortex was perceived as moving smoothly when, at each saccade landing, it jumped backwards to the position where it had been during saccade planning. In a control fixation task, participants perceived the motion as smooth, without jumps. We conclude that the pursuit system cannot incorporate the movement of motion patterns, despite our earlier reported findings that participants can accurately perceive such motion. Additionally, a subsequent direction discrimination task with varying stimulus duration showed that non-rigid motion processing takes longer than rigid motion processing. We propose a separate non-rigid motion processing pathway that does not feed into the pursuit system.

VISUO-MOTOR

BENNETT LECTURE THEATRE 8 - 11:00- 11:20

Nasal-temporal Differences in the Remote Distractor Effect: How the Presence of Placeholders Affects Saccade Latencies

Soazig Casteau, Robin Walker and Daniel Smith

Durham University, United Kingdom
soazig.casteau@durham.ac.uk

The remote distractor effect (RDE) is a robust phenomenon whereby an increase in saccade latency is observed when a remote distractor appears simultaneously with a target. However studies with hemianopes reported conflicting findings, whilst Rafal et al. (1990) described an inflated RDE when distractors were presented in the temporal hemifield, Walker et al. (2000) reported no RDE when distractors were presented to either the blind nasal or the blind temporal hemifield. To understand these opposite results, in a first study, we investigated the inhibitory effect of a distractor on saccade latency in normal human subjects. Participants were tested monocularly and we compared the effect of a nasal/temporal distractor in the presence or absence of a placeholder. Interestingly, when placeholders were used, we observed a RDE solely when the distractor was nasal. One explanation for this finding is that the sudden onset of the placeholders triggered a transient reflexive shift of attention, followed by a sustained Inhibition of Return. In a second study, we tested this assumption using the same paradigm but manipulating the timing of the placeholder onset. Overall, our results suggest that placeholders may sustain the engagement of additional inhibitory/attentional processes that bias selection towards stimuli in the nasal hemifield.

VISUO-MOTOR

BENNETT LECTURE THEATRE 8 - 11:20- 11:40

Neural Correlates of Handedness Related Modulation of the Vestibular-Ocular Reflex

Qadeer Arshad, Angela Bonsu, Mishaal Sharif, Giuseppe Gava and Adolfo Bronstein

Division of Brain Science, Imperial College London; inAmind laboratory, University of Leicester., United Kingdom
qa15@leicester.ac.uk

Stable visual perception during head movements is facilitated by the vestibular-ocular reflex. This reflex performs this function by generating compensatory eye movements that are of the same velocity but in the opposite direction to the head acceleration. Our previous research has demonstrated that when we combine viewing of binocular rivalry (i.e. different visual stimuli are presented to each eye so the brain cannot fuse the images into a single coherent percept) with vestibular stimulation that preferentially recruits the left hemisphere, it results in an asymmetrical modulation of the vestibular-ocular reflex (Arshad et al., 2013 JoNS). The observed modulation is handedness dependent, with right-handers suppressing left-beating nystagmus and left handers suppressing right-beating nystagmus. Here we sought to investigate the neural correlates of this modulation using 32-channel electroencephalography whilst subjects received combined visuo-vestibular stimulation. To assess modulation of eye movements we used video-oculography to measure the suppression in peak slow phase eye velocity. We observed that the stimulation technique modulated alpha activity, specifically focused over parietal areas. Critically there was a correlation between alpha power modulation and the degree of nystagmus suppression. Our results demonstrate that top-down modulation of the vestibular-ocular reflex is associated with alpha rhythm activity and this may be mediated by cortico-thalamic interactions.

VISUO-MOTOR

BENNETT LECTURE THEATRE 8 - 11:40- 12:00

Sound Influences Visually-guided Eye and Hand Movements during Manual Interception

Anna Schroeger, Philipp Kreyenmeier, Markus Raab, Rouwen Cañal-Bruland and Miriam Spering

Justus Liebig University Giessen; Friedrich Schiller University Jena, Germany
annaschroeger@gmail.com

Accurate processing of motion information is necessary in order to intercept moving objects. Most research emphasizes the role of visual input for motion prediction, neglecting the contribution of other sensory cues. For example, humans naturally associate loud batting sounds to harder hits and higher ball velocities. We hypothesized that observers integrate auditory cues with visual motion information to inform eye and interceptive hand movements. We presented the initial 100 or 300 milliseconds of a simulated baseball flight curve, accompanied by a batting sound of varying intensities. After tracking the ball with their eyes, participants intercepted it at a predicted location using their right index finger. Eye and hand positions at the moment of interception were affected by sound intensity: louder sounds resulted in overshooting of the target trajectory, implying that target speeds were overestimated. Interestingly, this finding was only observed for the short presentation time (100 ms), indicating that auditory cues are mostly used when visual information is sparse. Eye position data revealed that the influence of the batting sound emerged approximately 250 ms prior to interception. Our findings suggest that observers integrate visual and auditory cues for motion prediction, especially under visual uncertainty.

BILINGUAL READING I

BENNETT LECTURE THEATRE 8 - 13:00- 13:20

Bilingual Parafoveal Processing During Reading: Orthographic Preview Benefits in L1 and L2

Simon Tiffin-Richards and Ascensión Pagán

Univerity of Würzburg, Germany
s.tiffin-richards@posteo.net

We examined the amount of orthographic information extracted from the parafovea during sentence reading in German-English unbalanced bilinguals and monolingual English-speakers, using the boundary paradigm (Rayner, 1975). Participants read cognate target words embedded in sentences while their eye movements were recorded. Ninety sentences were created with identical word order in English and German. Three previews were generated from each target word: identity (Hamster/hamster), transposed-letter (Hasmter/hasmter), and substituted-letter (Harvter/harvter). Bilinguals read half of the sentences in English and half in German, while monolinguals read all sentences in English. Results showed an orthographic preview benefit in single, first fixation, and gaze durations: Fixation durations were shorter for the identity and transposed-letter previews than the substituted-letter previews. Bilinguals exhibited this benefit in both L1 (German) and L2 (English), although the orthographic preview benefit was greater in their L1, suggesting that language proficiency influences parafoveal processing. Although bilinguals showed slower reading times overall for English sentences than English-monolinguals, their orthographic preview benefit was similar. These findings suggest that proficient unbalanced bilinguals can extract orthographic parafoveal information in L2 as efficiently as English-monolinguals. This study extends previous evidence by comparing parafoveal processing between readers’ first and second language and between L1 and L2 language-users.

BILINGUAL READING I

BENNETT LECTURE THEATRE 8 - 13:20- 13:40

Semantic and Orthographic Parafoveal Processing in Bilingual Readers

Leigh Fernandez, Christoph Scheepers and Shanley Allen

University of Kaiserslautern, Germany
leigh.fernandez@sowi.uni-kl.de

Recently, a GCB1 study investigating parafoveal processing in L1-English and L1-German/L2-English, found that L2ers derived an interference from a non-cognate translation parafoveal mask (arrow vs. pfeil(translation:arrow)), but derived a benefit from an orthographic parafoveal mask (arrow vs. pfexk) in English2. Suggesting bilingual readers incurred a switching cost from the complete German parafoveal word, but derived a benefit by keeping both lexicons active from the partial German parafoveal word. In this registered report (IPA)3, we replicate and expand on this with three additional masks/comparisons. (1) English pseudo-word (Clain) to test whether facilitation from the orthographic mask was due to orthographic overlap, or because the mask is “word-like”3. (2) English-word condition (Array) to test whether the semantic information between the German word and English translation (Pfeil and Arrow) leads to the inhibition (language switch) or whether it’s the non-identical word is in the parafovea that leads to inhibition. (3) Comparison between the English translation and the two English non-words (Arrow vs. Arrzm+Clain) to test whether the non-word masks cause less inhibition than a real L2 word. This would provide further evidence of semantic facilitation and interestingly evidence that, unlike L1ers, L2ers show inhibition for L2 words and facilitation from non-words.

BILINGUAL READING I

BENNETT LECTURE THEATRE 8 - 13:40- 14:00

Your Eyes Tell Your Story: How Eye-movement Patterns during Natural Reading Develop with L2 Proficiency

Shiyu He, Dagmar Divjak and Petar Milin

University of Birmingham, United Kingdom
SXH1202@student.bham.ac.uk

The effects of length and frequency of the currently fixated word (n) (Reichle et al., 1998) and neighbouring words n-1 and n+1 (Heister et al.,2012) on fixations on word n in L1 skilled readers are well-studied. However, little is known about how these effects differ in L2 readers, particularly, how fixations change as proficiency in L2 changes. This study compares the eye-movements of 40 native speakers and two groups of Chinese L2 English readers with different English proficiency levels (27 intermediate, 12 advanced) while reading news excerpts. Nested linear mixed-effects models reveal that the length and frequency of word n, as well as the frequency of words n-1 and n+1 exhibited similar effects on the First Fixation Duration, Gaze Duration, and Total Reading Time on fixated word n across the three groups. However, the length of words n-1 and n+1 had distinct effects on three measures for both Chinese groups, with the more advanced group resembling the native group more closely. Our results present different parafoveal processing and attentional distributions of three groups which suggest gradual shaping of native-like patterns in L2 readers. Practice effectively helps eye-movement control even in the case of a rather disparate L1/L2 combination.

SYMPOSIUM TO HONOUR ALEXANDER POLLATSEK'S LEGACY TO EYE MOVEMENT RESEARCH

BENNETT LECTURE THEATRE 1 - 09:30- 12:00

Chair: Jukka Hyönä

University of Turku, Finland
hyona@utu.fi

Symposium Organiser: Jukka Hyönä (University of Turku)

The suggested symposium pays tribute to Alexander Pollatsek's legacy and contribution to eye movement research. He belongs to the pioneers on whose shoulders we have been able to stand when designing our own studies and making our own contributions. He has been very instrumental in putting forth formal models of eye movements in reading, both for alphabetic and logographic script (see Li's presentation). He has also published highly influential studies on the activation of the phonological code and orthographic neighbors (see Perea's presentation) during word recognition in reading, as well as on the recognition of compound and other morphologically complex words (see Hyönä's and Liversedge's presentations). Sandy also successfully applied the eye-tracking method to study visual cognition, particularly object and scene perception (see Castelhano's presentation). Finally, his statistical expertise is widely appreciated (see Drieghe's presentation). In the proposed symposium, we pay tribute to different aspects of his research.

SYMPOSIUM TO HONOUR ALEXANDER POLLATSEK'S LEGACY TO EYE MOVEMENT RESEARCH

BENNETT LECTURE THEATRE 1 - 09:30- 09:50

Reading Compound Words in Finnish and Chinese: An Eye-tracking Study

Jukka Hyönä, Lei Cui, Birgitta Paranko and Timo Heikkilä

University of Turku, Finland
hyona@utu.fi

Two eye-tracking experiments are reported that examined the recognition of two-constituent compound words in alphabetic Finnish and logographic Chinese. In both languages, the majority of dictionary entries are compound words comprising two (or more) morphemes. In Finnish, two-constituent compound words vary greatly in length (aikaero = time difference vs. autovuokraamo = car rental), whereas in Chinese they are identical in length (时差 = time difference vs. 车行 = car rental). According to the visual acuity principle (Bertram & Hyönä, 2003), short Finnish compound words and all two-character Chinese compound words fit in the foveal vision and are thus recognized holistically, whereas long Finnish compound words are recognized via their components. The results are in line with the visual acuity principle. In Finnish, the effect of first constituent frequency indexing processing via components was significant for long compounds in gaze duration and probability of refixation, but not for short compounds. In Chinese, the first-constituent frequency effect was non-significant in gaze duration for the whole word and for the 1st and 2nd character. The Chinese results are also compatible with the Chinese Reading Model (Li & Pollatsek, 2020), according to which whole-word representations overrule the activation of individual characters.

SYMPOSIUM TO HONOUR ALEXANDER POLLATSEK'S LEGACY TO EYE MOVEMENT RESEARCH

BENNETT LECTURE THEATRE 1 - 09:50- 10:10

Scene Perception through Time and Space

Monica S. Castelhano

Queen's University, Canada
monica.castelhano@queensu.ca

Although known for reading, Sandy Pollatsek also explored how the effects of scene semantics affected attention, memory, and object recognition. From his early studies to much later ones, he used his knowledge of reading and eye movements to explore how parallel semantic associations may help to explain processing of scene images. Here, I will speak about his past work, the studies we conducted together on this topic and more recent work that was directly inspired from interactions with Sandy over the years. By examining how different aspects of scenes are processed over time, from the spatial layout to the identification of individual objects, I will show the over overlooked legacy of Sandy Pollatsek to the understanding of scene perception.

SYMPOSIUM TO HONOUR ALEXANDER POLLATSEK'S LEGACY TO EYE MOVEMENT RESEARCH

BENNETT LECTURE THEATRE 1 - 10:10- 10:30

Words, Letters, and the Front-end of Word Identification and Reading

Manuel Perea and Ana Marcet

Universitat de València, Spain
mperea@uv.es

This talk offers a quick snapshot of the travel from sensory information to letters and words during reading, one of the many topics tackled by Sandy Pollatsek. In 1998-1999, Sandy and I showed that one-letter different neighbors (space for the word spice) of a target word affected sentence reading. These insights yielded an ampler definition of a “neighbor” (e.g., addition or deletion-letter neighbors [horse vs. house], transposed-letter neighbors [calm vs. clam]). Related to the previous point and following Sandy’s ideas, it is critical to focus not only on English but also on very diverse orthographies. In this line, new efforts are devoted to clarifying the models' front-end by examining the role of diacritical vowels on word recognition and reading. We have recently shown that the omission of diacritics does not hinder (or only minimally) sentence reading in Spanish—in Spanish, diacritics only mark the stressed vowels. Critically, this pattern may change for those languages in which diacritics have other functions (e.g., vowel quality, length, tone, etc.). Further work in different languages and paradigms is essential to shed more light on the front-end of models of reading.

SYMPOSIUM TO HONOUR ALEXANDER POLLATSEK'S LEGACY TO EYE MOVEMENT RESEARCH

BENNETT LECTURE THEATRE 1 - 10:30- 10:50

A Multiverse Exploration of Choices in Cleaning and Analysing Eye Movements during Reading

Denis Drieghe, Charlotte Lee and Hayward Godwin

University of Southampton, United Kingdom
D.Drieghe@soton.ac.uk

Datasets from eye tracking research during reading inevitably involves processing raw data into a form suitable for statistical analyses. For a reading experiment, this will typically include deciding whether to merge small fixations located close to each other, and whether to remove very short and/or very long fixations. Outlier analyses can include removing fixations 3 or 2.5 standard deviations from the grand mean, the overall participant mean or the mean of a participant for a specific condition. Subsequently, Linear Mixed Models are often reported on untransformed, log-transformed or using GLMMs which do not assume an underlying normal distribution (Lo & Andrews, 2015). Here we conducted a multiverse analysis, which consisted of running thousands of LMMs representing all reasonable combinations of choices in cleaning and analysing fixation data. In this talk, we will explore the impact of these choices focusing on the reported size of the frequency effect during a reading experiment using an EyeLink 1000. Amongst our findings is that the frequency effect in single fixation durations can vary by more than 5 milliseconds depending on the data processing choices made, even though we restricted ourselves to cleaning and analysis methods that would be considered acceptable practice by researchers.

SYMPOSIUM TO HONOUR ALEXANDER POLLATSEK'S LEGACY TO EYE MOVEMENT RESEARCH

BENNETT LECTURE THEATRE 1 - 10:50- 11:10

Operationalisation of Processes Over Linguistic Units in Reading: Cross-linguistic, Acuity and Lexical Processing Considerations

Simon P. Liversedge

University of Central Lancashire, United Kingdom
spliversedge@uclan.ac.uk

To mark the immense contribution to the field of eye movements and human visual cognition that Sandy Pollatsek made during his career, I will try to deliver a talk that has an empirical basis, challenges current thinking, and hopefully, stimulates discussion (all things he valued). I will consider how visual and cognitive processes are operationalised over linguistic units across fixations during natural reading. The empirical basis of the talk will be results from a series of eye movement experiments that my Chinese colleagues and I have recently conducted to explore the Multi-Constituent Unit Hypothesis (Zang, 2020). These experiments have caused us to think that processing occurs serially and sequentially, but with respect to lexical units that may be larger than individual words. Such a suggestion might apply for unspaced languages (e.g., Chinese), agglutinate languages (e.g., Finnish) as well as (non-agglutinate) spaced languages (e.g., English). Additionally, I will suggest that current models of lexical processing do not adequately map onto natural reading circumstances. At a minimum, ecologically valid models of lexical identification require processing mechanisms to support successive (fixation-by-fixation) episodic delivery of visual information that is modulated by acuity limitations based on (launch and landing) fixation sites.

SYMPOSIUM TO HONOUR ALEXANDER POLLATSEK'S LEGACY TO EYE MOVEMENT RESEARCH

BENNETT LECTURE THEATRE 1 - 11:10- 11:25

Sandy Pollatsek’s Legacy to Visual Cognition

John Henderson

University of California, Davis, United States
johnhenderson@ucdavis.edu

There is no abstract for this contribution.

SYMPOSIUM TO HONOUR ALEXANDER POLLATSEK'S LEGACY TO EYE MOVEMENT RESEARCH

BENNETT LECTURE THEATRE 1 - 11:25- 11:40

Eye Glance Behaviors: Their Role in Theory and Practice

Donald Fisher

University of Massachusetts Amherst, United States
fisher@ecs.umass.edu

Our theoretical understanding of eye glance behaviors in scene recognition, visual search, and attention has advanced considerably since the 1970s. Our understanding of the role of eye glance behaviors in the more practical tasks such as reading has also advanced. Much more recently, those behaviors have been shown to be critical to understanding just how engaged drivers are in the dynamic driving task, why novice drivers are more likely to crash than experienced drivers, why drivers with ADHD are more at risk than neurotypical drivers, why older drivers are especially at risk in intersection crashes, and whether drivers can be trained to decrease their crash risk, to name just a few examples. No one has put together the various theoretical advances with the more applied advances. Alexander Pollatsek has been critical to this integration. His many seminal contributions to this integration are discussed and highlighted.

SYMPOSIUM TO HONOUR ALEXANDER POLLATSEK'S LEGACY TO EYE MOVEMENT RESEARCH

BENNETT LECTURE THEATRE 1 - 11:40- 11:55

Exploring Mechanisms of Chinese Reading with Sandy Pollatsek

Xingshan Li

Institute of Psychology, Chinese Academy of Sciences, China
lixs@psych.ac.cn

As a logographic writing system, Chinese has many unique properties compared with alphabetic writing systems such as English. One important unique property is that there are no interword spaces to demarcate words in Chinese. This raises important questions regarding how Chinese readers segment words and how they choose their saccade targets. To understand how Chinese readers conquer this challenge, Sandy Pollatsek and I conducted a series of experiments together in the last 15 years. Based on the results of these findings, we also constructed a computational model of word processing and eye movement control during Chinese reading (CRM). These works have been valuable to understand the unique mechanism of Chinese reading. In this talk, I will share some of my memories on working with Sandy on these projects.

HIGHER-LEVEL II

BENNETT LECTURE THEATRE 1 - 13:00- 13:20

Search for the Unknown: Guidance of Visual Search in the Absence of an Active Template

Oryah Lancry-Dayan and Yoni Pertzov

Hebrew University of Jerusalem, Israel
oryah.lancry@mail.huji.ac.il

Can you efficiently look for something even without knowing what it looks like? According to theories of visual search, the answer is no: A template of the search target must be maintained in an active state to guide search for potential locations of the target. Here, we tested the need for an active template by assessing a case in which this template is improbable: the search for a familiar face among unfamiliar ones when the identity of the target face is unknown. Since people are familiar with hundreds of faces, an active guiding template seems unlikely in this case. Nevertheless, participants were able to guide their gaze towards the target as long as extrafoveal processing of the target features was possible. Additionally, individual's differences in the ability to process familiarity through extrafoveal vision were correlated with proficiency in the search task. These results challenge current theories of visual search by showing that guidance can rely on long-term memory and extrafoveal processing rather than on an active search template.

HIGHER-LEVEL II

BENNETT LECTURE THEATRE 1 - 13:20- 13:40

Attenuation of Visual Exploration Due to Accessing of Internally Stored Representations

Tal Nahari, Eran Eldar and Yoni Pertzov

Hebrew University of Jerusalem, Israel
tal.nahari@mail.huji.ac.il

Have you recently failed to notice your roommates' new poster or plants, or worse – your friends' new haircut? We hypothesized that such failures are the result of attenuation of visual exploration that happen due to an internally stored memory that is the subject of our investigation. In order to test this hypothesis, we designed an experiment including either visually familiar or non-familiar stimuli, in situations of encoding and retrieval. We show that both during encoding and retrieval, a unique pattern of gaze behavior towards the familiar percept is reflected by fewer and longer fixations. Thus, the results suggest that visual exploration is indeed attenuated as the informational gaps are filled by accessing existing mental representations. This research sheds light on the relationships between internal and external exploration mechanisms by bringing forward the tradeoff between the two processes.

HIGHER-LEVEL II

BENNETT LECTURE THEATRE 1 - 13:40- 14:00

Investigating the Role of Theory of Mind on the Processing of Dramatic Irony Scenes in Film

Cynthia Cabañas, Atsushi Senju and Tim J. Smith

Birkbeck, University of London, United Kingdom
ccaban01@student.bbk.ac.uk

Watching cinema involves interpreting characters' mental states through a process known as Theory of Mind (ToM). To investigate these processes and how the viewer's mental model of the character's mental state influence how they attend to and process the film, we focused on dramatic irony scenes, where the audience knows something that one of the characters does not. In a between-subjects design, we provided critical information that the characters did not possess to some viewers (dramatic irony group) and not to others (control group, which had the same information as characters), thereby creating different event models of the characters' mental states. We hypothesized that if the dramatic irony group build more complex ToM event models that these processing differences will be evident during the film viewing as deeper cognitive processing. Combining eye tracking with a self-paced film-viewing task we will examine: (a) pupil dilation as an indirect marker of processing load and (b) the analysis of spatio-temporal distribution of overt attention, i.e. what part of the images participants are processing differently across conditions, informing us about how participants extract information about characters' mental states, and whether there are longer viewing times when event models are switched/updated to follow character perspectives.

HIGHER-LEVEL II

BENNETT LECTURE THEATRE 1 - 14:00- 14:20

Mental Detection Using Eye Movements. ~ Eyes Tell You the Mental Status ~

Ayumi Takemoto, Inese Aispuriete, Laima Niedra and Lana Franceska Dreimane

University of Latvia, Latvia
ayutakemo@gmail.com

Since after covid pandemic began, the population of patients with depression has been skyrocketing. Sometimes depression is hard for patients to recognize if they have it or not by themselves, especially in the early stage. Our main goal is to develop systems to detect the early stage of depression using eye movements data while they are talking to virtual avatars or humans such as their friends, families, or colleagues, in their daily life.

We will report the result which focused on whether eye movements reflect depression while people are talking to virtual avatars in the non-clinical interviews. We collected eye-tracking data of participants with or without depression while they were interacting with a virtual avatar or human interviewer and computed statistical analysis. As the result, eye movements are possible to be one of the criteria to classify people as being with or without depression.

This research was supported by European Regional Development Fund (ERDF) for Post-doc projects grant agreement No 1.1.1.2/VIAA/4/20/668.

BILINGUAL READING II

BENNETT LECTURE THEATRE 1 - 15:30- 15:50

Evaluating the Vocabulary Coping Strategies of L2 Readers Through Eye-tracking

Caleb Prichard and Andrew Atkins

Okayama University, Japan
calprich@gmail.com

Language learners often struggle reading in the target language due to a lack of vocabulary (Grabe & Stoller, 2013). Effective vocabulary coping strategies are essential and include three main options: trying to infer word meaning, ignoring the word, and dictionary use. However, educators have differing philosophies on the most effective strategies, and learners may be overly reliant on a certain strategy. Prior research on this area has been dominated by surveys and think-aloud protocols. However, participants may not be fully aware of their strategy use, or they may report based on what they perceive is the ideal response.

This presentation overviews how eye tracking has been utilized to more empirically examine vocabulary coping strategies. Two studies involved Japanese college-aged learners of English (N = 57 and 39) in task-based reading. Eye tracking revealed that, in cases where a novel word was mid-sentence, most participants did not even fixate on context cues following the word before checking the dictionary. Participants who made regressions after fixating on a pseudoword to re-read the context before looking up the word performed better on the post-reading tasks. Other eye movement data and implications for researchers and educators will be overviewed.

BILINGUAL READING II

BENNETT LECTURE THEATRE 1 - 15:50- 16:10

Processing and Comprehension of Arguments by Chilean Primary School Students

Claudia Guerra and Romualdo Ibañez

Pontificia Universidad Católica de Valparaíso, Chile
claudia.guerra@pucv.cl

In the majority of arguments used in Chilean primary school textbooks, major premises -the ones that guarantee the logical link between the minor premises and the conclusion- are kept implicit. This pattern slows down processing and hinders comprehension, especially among poor readers (Malaia, Tommerdahl & McKee, 2015; Prado, Spotorno, Koun, Hewitt, Van der Henst, Sperber, Noveck, 2015). To explain the phenomenon, it has been hypothesized that, faced with the absence of the major premise, readers do not elaborate the logical connections (Britt, Kurby, Dandotkar & Wolfe, 2008). Given that readers may have an insufficiently precise representation of the text, discourse markers may facilitate this reading (van Silfhout, Evers-Vermeul & Sanders, 2015). To assess whether this is the case, Chilean 6th and 8th graders read short argumentative texts while their eye movements were recorded. Each text included one of four conditions, considering two levels for each variable: major premise (implicit and explicit) and marker (implicit and explicit). Preliminary results indicate that the target sentences, minor premise and conclusion, were read faster and adequately understood with the conditions explicit premise-connector and implicit premise-explicit connector. This means that there was no effect on the degree of explicitness of the major premises. The explicitness of this premise does not result in more efficient processing of arguments, nor does it guarantee adequate comprehension, as compared to the presence of connectors.

READING COMPREHENSION

BENNETT LECTURE THEATRE 2 - 13:00- 13:20

How Do We Resume Our Reading After an Interruption? Effects of Interruption on Eye Movements and Reading Comprehension

Véronique Drai-Zerbib and Chevet Guillaume

University of Burgundy, France
Veronique.Drai-Zerbib@u-bourgogne.fr

Reading is increasingly taking place on digital support, which is vector of interruptions that disrupt the attention during reading. This study reports the consequences of an interruption on reading behavior, and text comprehension for the information being read precisely when the interruption occurs. 38 participants read four long procedural texts while we recorded their eye-movements (mean number of fixations, regressive fixations and mean fixation duration). The readers were interrupted by an arithmetic verification task either in the middle of the paragraph (intra-paragraph condition) or between two paragraphs (inter-paragraph condition). In the intra-paragraph condition, the eye movement analysis revealed more re-reading behavior when an interruption occurred. We assessed comprehension with 48 questions and separated our participants into two groups (high and low comprehenders) based on the median of the error rate. The high comprehenders showed more re-reading behavior (fixation and regressive fixation) in the sentence including the target information than low comprehenders. However, independently of the position of the interruption (inter or intra-paragraph) the comprehension performances were not degraded. Those results are discussed in regard to long term working memory (Ericsson & Kintsch, 1995).

READING COMPREHENSION

BENNETT LECTURE THEATRE 2 - 13:20- 13:40

Effects of “Desired Difficulty” on Eye Movements and Comprehension in Reading

Christian Vorstius, Laura Schwalm and Ralph Radach

Bergische Universität Wuppertal, Germany
vorstius@uni-wuppertal.de

The concept of desired difficulty assumes that perceived increased task difficulty may lead to deeper processing and benefit memory performance. During reading, this idea can take the form of using typographically altered fonts, e.g., Sans Forgetica (SF), a typeface specifically designed for this purpose. The present study examines the impact of these letter alterations in terms of moment-to-moment processing and text comprehension. Participants read non-fiction texts while their eye movements were recorded. After practice with the new font, each participant read two texts in Arial and two in SF. Following the Construction-Integration-Model (Kintsch, 1988), text comprehension was calculated on the levels of propositional text base and situation model. We found a substantial inflation of early and late viewing time parameters when reading the SF font. These effects were most prominent at the beginning of the experiment. Interestingly, an expected interaction between font type and word length was lacking. In addition, no difference on either level of comprehension was found between the two fonts. Apparently, linguistic processing and oculomotor control was adjusted to meet a certain comprehension criterion. We show how this global adjustment (Radach, Huestegge & Reilly, 2008) is being implemented in local fixation patterns and discuss theoretical implications.

READING COMPREHENSION

BENNETT LECTURE THEATRE 2 - 13:40- 14:00

The Role of Context in the Processing of Semantic Ambiguities: Eye-tracking Evidence from Younger and Older Adults

Tami Kalsi, Kevin Paterson and Ruth Filik

University of Nottingham, United Kingdom
tami.kalsi@nottingham.ac.uk

Doubly quantified sentences like “Every kid climbed a tree” are ambiguous regarding whether there is one tree or multiple trees. Previous research has shown that grammatical factors (e.g., the order in which the quantifiers appear) do not affect the general preference for a singular entity, evidenced in shorter reading times for “The tree was” than “The trees were” in a subsequent sentence. However, the influence of contextual information has received little attention. In the current study, 48 younger (18-30) and 48 older adults (65+) had their eye movements monitored while they read sentences in which the context was biased towards either singular (e.g. “Every student in the class is listed on a register.”) or plural entities (e.g. “Every school pupil in the country has their attendance marked on a register.”). A singular (“This register…”) or plural continuation (“These registers…”) then followed. Results showed that contextual bias influenced on-line linguistic processing, with longer reading times for continuations which were incongruent with the context. Also, evidence of greater processing difficulty was identified when contextual factors were in opposition with grammatical factors, especially for older adult readers. The present findings support a parallel constraint-satisfaction approach under which multiple factors interact during on-line processing.

READING COMPREHENSION

BENNETT LECTURE THEATRE 2 - 14:00- 14:20

Effects of Reading Goals on Processing of Syntactic Ambiguity, Semantic Plausibility and Sentence Wrap-up: Insights from Eye Movement Behaviour

Fawziah S. Qahtani, Kayleigh L. Warrington, Kevin B. Paterson and Sarah J. White

University of Leicester, United Kingdom
fshq1@leicester.ac.uk

While many studies have examined the mechanisms underlying reading for comprehension, relatively few have directly examined the effects of readers' goals on eye movement behaviour (e.g., Fitzsimmons et.al., 2020; Strukelj & Niehorster, 2018). Here we present three experiments that specifically examined how reading goals modulate the processes involved in sentence integration. Specifically, we investigated how different goals (reading for comprehension versus scanning for a topic [Experiment 1 & 2], skimming for gist versus scanning for a topic [Experiment 2]), modulate effects of syntactic ambiguity (Experiment 1), semantic plausibility (Experiment 2) and sentence wrap-up (Experiment 3). Reading times were longer for reading compared to scanning for a topic, and longer when skimming for gist compared to when scanning for a topic. Crucially, the results showed that effects of ambiguity, plausibility and sentence wrap-up were similar during the initial processing of critical words across all reading goals. However, for measures sensitive to rereading, (regression path duration), effects were larger for reading compared with scanning for a topic. The results build on previous work (Weiss et. al., 2018) suggesting that sentence integration processes can be modulated by task demands. We discuss the implications for theories of eye movement control during reading.

READING COMPREHENSION

BENNETT LECTURE THEATRE 2 - 14:20- 14:40

How Does Word Order Influence Natural Reading?

Petar Atanasov, Simon P. Liversedge and Federica Degno

University of Central Lancashire, United Kingdom
PAtanasov@uclan.ac.uk

The parallel processing OB1 Reader Model (Snell et al., 2018) postulates that multiple words are lexically processed in parallel and can be assigned to their correct position by a cognitive mechanism to form a valid sentence frame regardless of their order of presentation during natural reading. In this talk, we present an eye movement study that tested how word transpositions and grammatical well-formedness affect reading. In the current experiment, we presented 5-word sentences in which the positions of the third and fourth words (the target pair of words) and the identity of the final word (the post-target) were orthogonally manipulated to produce grammatical violations. Our results showed that the word transposition did not cause any disruption to the processing of the first two words in the sentence, consistent with the suggestion that lexical and syntactic information was not extracted from the parafovea and that words are lexically processed in a serial rather than parallel fashion. Moreover, the transposition produced significant disruption on the third word of the sentence, with longer reading times when the two targets were transposed compared to not transposed, suggesting that the order in which words are presented is important for natural reading.

Snell, J., van Leipsig, S., Grainger, J., & Meeter, M. (2018). OB1-reader: A model of word recognition and eye movements in text reading. Psychological Review, 125(6), 969- 984. https://doi.apa.org/fulltext/2018-37844-001.html

READING COMPREHENSION

BENNETT LECTURE THEATRE 2 - 14:40- 15:00

How Early Do Readers Extract the Meaning of an Emoji?: Evidence from Eye Movements

Heather Sheridan, Andriana Chistofalos, Eliza Barach and Laurie B. Feldman

State University of New York, Albany, NY, United States
hsheridan@albany.edu

Parafoveal-on-foveal (PoF) effects, in which a parafoveal word (n+1) influences processing of the foveal word (n), have previously been interpreted as possible support for parallel instead of serial processing during word recognition. Here, we document the influence of an emoji's meaning on its preceding target. We examined eye movements while participants read sentences containing a target word (e.g., coffee in the sentence "I enjoyed my tall coffee") that was immediately followed either by no emoji, a semantically congruent (e.g., coffee cup) or an incongruent (e.g., beer mug) emoji. First-pass fixation durations were shorter on the foveal target word (n) when the parafoveal emoji (n+1) was semantically congruent rather than incongruent (i.e, an emoji-elicited semantic PoF effect), which suggests that emojis and text can potentially be processed in parallel. Building on these results, we conducted E-Z Reader simulations to explore if a serial model of eye movement control could be extended to emojified text. Our simulations indicated that emojis require more time for perceptual and cognitive processing compared to three-letter nouns that were equated to the emojis in length. We discuss how models of eye movement control during reading could be modified in the future to accommodate emojified text.

EYE MOVEMENT CONTROL IN READING III

BENNETT LECTURE THEATRE 2 - 15:30- 15:50

Reader Targeting of Words is Guided by the Distribution of Information in the Lexicon

Jon Carr and Davide Crepaldi

SISSA, Italy
jcarr@sissa.it

Skilled readers typically identify words more accurately when fixating them slightly left of the central character, the so-called optimal viewing position. There are two main explanations for this effect, which are not mutually exclusive. The first claims that the optimal viewing position lies left-of-center due to the particular constraints of the human perceptual system. The second explains the effect in terms of how words compete with each other; specifically, the beginnings of words tend to be more unique and therefore more informative about word identity, making a left-of-center fixation more advantageous. We explore this effect through the lens of a Bayesian cognitive model and two experiments using artificial lexicons in which we can carefully control how information is distributed across wordforms. Our results suggest that readers are sensitive to the distribution of information, targeting different positions depending on whether the language they learned is more informative on the left or right. Furthermore, readers do not simply target the position that contains the most information; rather, they target the position that will yield the best view of the word overall, accounting for both information distribution and the asymmetry of the human visual span.

EYE MOVEMENT CONTROL IN READING III

BENNETT LECTURE THEATRE 2 - 15:50- 16:10

Prismatic Glasses Affect the Binocular Coordination during Reading

Stephanie Jainta and Joëlle Joss

SRH University of Applied Sciences in North Rhine-Westphalia, Germany
stephanie.jainta@srh.de

Binocular coordination (saccade disconjugacy, vergence drifts and fixation disparity) and binocular advantages in reading (shorter total reading times and fixation durations in binocular compared to monocular reading) typically vary with the amount of individual, horizontal heterophoria, i.e., a resting position of vergence eye movements. Since prismatic glasses (identified via MCH-method) change heterophoria, we investigated whether prismatic glasses also affect different aspects of binocular coordination during reading. We collected binocular eye movement data (Eyelink II) for 54 participants in four reading conditions (30 sentences each): 1) monocular, 2) binocular, 3) binocular with added disparity (caused by MCH prisms) and 4) binocular with added disparity after 6 months. During the 6 months delay, 29 participants wore refractive glasses including prismatic effects (mean heterophoria: 2.1 pdpt) and 25 participants wore refractive glasses only (mean heterophoria: 2.5 pdpt). While all participants showed some binocular advantage for all binocular reading conditions, the binocular advantage tentatively increased further for our prism group after 6 months. Furthermore, saccade disconjugacy increased slightly and objective fixation disparity was significantly reduced after 6 months. Thus, after some time, individual prismatic glasses (MCH) changed aspects of the binocular coordination in reading and slightly increased the binocular advantage for our heterophoric participants.

EYE MOVEMENT CONTROL IN READING III

BENNETT LECTURE THEATRE 2 - 16:10- 16:30

The Role of the Periphery in Comic Reading

Clare Kirtley, Christopher Murray, Phillip Vaughan and Benjamin Tatler

University of Aberdeen, United Kingdom
clare.kirtley1@abdn.ac.uk

While there is growing evidence that reading comics is sequential and ordered, like reading, we currently lack an understanding of the processing mechanisms that underlie comic reading. A key issue is the influence of peripheral information on the reading process. Using the moving-window and invisible boundary paradigms, across three studies we investigate whether comic reading is disrupted when peripheral information is removed in single line comic strips (Experiment 1) and multi-line comics (Experiment 2); as well as what more detailed image information is extracted from upcoming panels (Experiment 3). The studies show that readers are aware of the content of at least the next panel, extracting both morphological and semantic information from the images, and identifying the location of text before moving to the next panel. The findings add further evidence to the idea of reading comics as an ordered process, and the existence of a visual language, with its own narrative grammar.

CLINICAL AND APPLIED II

BENNETT LECTURE THEATRE 8 - 13:00- 13:20

Active Vision in Sight Recovery Individuals with a History of Long-lasting Congenital Visual Deprivation

José P. Ossandón, Paul Zerr, Idris Shareef, Ramesh Kekunnaya and Brigitte Röder

University of Potsdam, Germany
laubrock@uni-potsdam.de

Restoring vision after congenital visual deprivation often results in incomplete functional recovery. Here we tested whether early visual experience is necessary for the development of systematic visual exploration. A free-viewing paradigm using real-world images was used to investigate the exploratory behavior of ten individuals born with bilateral dense cataracts whose sight had been restored after a prolonged period of severe visual deprivation. Their behavior was compared to three control groups: normally sighted age-matched controls, developmental cataract reversal individuals, and individuals with infantile nystagmus without a history of visual deprivation. Sight-recovery individuals presented moderate-to-severe visual impairments and gaze instability (nystagmus). However, their visual exploration patterns were successfully predicted by those of normally sighted controls and were indistinguishable from individuals with infantile nystagmus. Similar to all control groups, visual exploration in sight-recovery individuals was based on the low-level (luminance contrast) and high-level (object components) visual content of the images. Sight-recovery individuals' systematic visual exploration was associated with better object recognition, suggesting that active vision might be a driving force for visual system development and recovery. Overall, the present results argue against a sensitive period for the development of the neural mechanisms associated with active visual exploration.

CLINICAL AND APPLIED II

BENNETT LECTURE THEATRE 8 - 13:20- 13:40

Environmental Demand Influences Scanning Behaviour in People with Hemianopia

Eva Postuma, Ashkan Nejad, Gera de Haan, Joost Heutink and Frans Cornelissen

University of Groningen, Netherlands
e.m.j.l.postuma@rug.nl

Introduction

People with homonymous hemianopia (HH) can use scanning strategies that compensate for their visual field defect to overcome difficulties with mobility activities such as walking. However, environmental demands may influence the use of compensatory scanning. This study aims to examine the effect of increased environmental demands on compensatory scanning in people with HH.

Method

Ten participants with HH and ten with normal vision walked for 2 minutes in a quiet neighbourhood and a shopping centre while their gaze was recorded using a mobile eye tracker (Pupil Invisible). For both participant groups, we evaluated scanning behaviour in terms of exploration, length of scans, and distribution of scanning.

Results

Our preliminary results show that both people with HH and with normal vision increased their distribution of scanning and exploration when walking in the shopping centre compared to the quiet neighbourhood.

Conclusion

During walking, people with HH as well as people with normal vision adapt their compensatory scanning depending on environmental demand. Future research should investigate whether and how people with HH benefit from these changes in compensatory scanning and examine differences in changes in scanning behaviour between people with HH and normal vision.

CLINICAL AND APPLIED II

BENNETT LECTURE THEATRE 8 - 13:40- 14:00

Body (Dis)satisfaction in Transgender and Cisgender people: A Novel Eye-tracking Study to Explore Attentional Bias

Filipe Cristino and Beth A. Jones

Nottingham Trent University, United Kingdom
filipe.cristino@ntu.ac.uk

Previously, transgender people have been found to experience more body dissatisfaction in comparison to cisgender people. A link between attention, body perception and body dissatisfaction has been found but the role of eye movements is unclear. Particularly, it is not known if attention is deployed to particular body parts. Therefore, this study aimed to determine whether transgender and cisgender people show an attentional bias toward body parts they are dissatisfied with. To test this aim, we recruited 90 participants (30 transgender, 30 cisgender males and 30 cisgender females) and tracked their eye movements while viewing 3D scans of bodies. Participants were unaware of the true aim of the experiment and were asked to perform a body recognition task (learn a set of 12 3D bodies from different viewpoints and then perform a 2AFC recognition task). After this task, they were asked to complete a self-report questionnaire about body (dis)satisfaction. A novel method to map eye movement onto 3D bodies was used, allowing us to aggregate data across bodies and viewpoints. The three groups of participants performed similarly during the body recognition task, but significant differences were found when comparing gaze patterns between groups. Most importantly, we found a significant direct correlation between body dissatisfaction and fixations patterns, across all groups, but particularly for the transgender and female group. We found that the more a participant disliked a body part (e.g. the stomach area) the more they fixated that body part showing a clear cognitive bias towards disliked body parts.

CLINICAL AND APPLIED II

BENNETT LECTURE THEATRE 8 - 14:00- 14:20

Saccadic Temporal Prediction in Typically Developing Youth and in Psychiatric Adolescents with Impulsivity

Olivia Calancie, Donald Brien, Jeff Huang, Brian Coe, Linda Booij, Sarosh Khalid-Khan and Douglas Munoz

Centre for Neuroscience Studies, Queen's University, Canada
0oc7@queensu.ca

The motor learning process underlying the identification of a periodic stimulus and initiation of movements to synchronize to its arrival is known as temporal prediction. Temporal prediction is needed for motor coordination, yet little is known about its timeline of maturation across typical and atypical neurodevelopment. Here, we recorded saccades to regular and irregularly timed visual targets in typically developing youth (N=115; aged 6-24 years) and in adolescents with psychiatric diagnoses of the impulsivity domain (N=44; aged 11-18) to elucidate the typical development of predictive saccades (saccade reaction time [SRT] <90 ms; in anticipation of visual target) to 5 interstimulus intervals and compare behaviour among healthy and psychiatric groups. In addition to saccade metrics (e.g., reaction time, peak velocity, amplitude), we recorded participants' pupil size and blinks throughout the tasks to query how these behaviours varied according to whether participants predicted or reacted (SRT > 90 ms) to periodic targets. Indeed, when predicting targets, saccades were hypometric, pupil size was reduced, and blinks were suppressed prior to target appearance. The age of maturation for predictive saccades was ~14. Psychiatric adolescents, compared to age-matched controls, produced an increased frequency of predictive saccades towards irregular targets.

CLINICAL AND APPLIED II

BENNETT LECTURE THEATRE 8 - 14:20- 14:40

Pro- and Anti-saccade Parameters Reveal Discrete Neural Processes and Differentially Associate with Cognitive Domains in Neurodegenerative Disease

Heidi C. Riek, Brian Coe, Donald Brien, Jeff Huang, Agessandro Abrahao, Stephen Arnott, Derek Beaton, Malcolm Binns, Sandra Black, Elizabeth Finger, Morris Freedman, Donna Kwan, Anthony Lang, Brian Levine, Wendy Lou, Connie Marras, Mario Masellis, Paula McLaughlin, J. B. Orange, Angela Roberts, Stephen Strother, Kelly Sunderland, Richard H. Swartz, Brian Tan, Carmela Tartaglia, Angela Troyer, Lorne Zinman, The Ondri Investigators and Douglas Munoz
Queen's University, Canada, heidi.riek@queensu.ca

Saccades may provide effective biomarkers for neurodegenerative disease due to overlap between oculomotor circuitry and disease-affected areas. Accurate characterization of disease-related saccade behaviour, and its links to global and specific aspects of cognitive impairment, is crucial to biomarker development. To achieve this, we used data from the Ontario Neurodegenerative Disease Research Initiative, which recruited multiple large, directly comparable cohorts of patients with Alzheimer’s disease, mild cognitive impairment, amyotrophic lateral sclerosis, frontotemporal dementia, Parkinson’s disease, or cerebrovascular disease. Patients (n=450, age 40-87) and healthy controls (n=149, age 42-87) completed a randomly interleaved pro- and anti-saccade task; patients also completed an extensive neuropsychology battery. We explored the relationships of saccade parameters (e.g. task errors, reaction times) to one another and to neuropsychology-based cognitive domain scores (e.g. executive function, memory). Task performance consistently worsened with global cognitive impairment; subsets of saccade parameters were interrelated and also differentially related to cognitive domain scores (e.g. antisaccade errors and reaction time associated with each other and with executive function). This suggests the subsets may index temporally distinct brain processes that connect saccades to cognition and neurodegeneration, and may have implications for use of IPAST as a cognitive screening tool in these diseases.

CLINICAL AND APPLIED II

BENNETT LECTURE THEATRE 8 - 14:40- 15:00

Utility of Eye-tracking in Visual Cortical pProstheses – Preliminary Patient Testing Results

Avi Caspi

Jerusalem College of Technology, Israel
avi.caspi@jct.ac.il

Restoring functional sight requires that the electrical stimulation should convey information to the brain that is associated with the correct spatial location in the scene. A visual cortex stimulator can bypass the eye and the optic nerve and create phosphenes (perception of light) without light entering the eyes. Nonetheless, recently we demonstrated that eye movements dominate the perceived location of cortical stimulation-evoked phosphenes, even after years of blindness. In the current presentation we will present results from patients demonstrating the correlations between eye movements and the visual percepts.

We instruct patients to conduct an eye movement toward the phosphene and to use eye movements as a marker to construct the spatial map of the implanted electrodes. Experiments were conducted with blind patients implanted with the NeuroPace Responsive Neurostimulator (RNS) and the Orion visual cortical prosthesis devices.

In contrast to a retinal prosthesis, in a cortical visual prosthesis, the layout of the implanted array does not match a retinotopic map and it is necessary to find the visual-field location of the percept of each implanted electrode. To establish the spatial map of the electrodes, users were instructed to conduct an eye movement to the location of the phosphene generated by electrical stimulation of the occipital lobe. Two different schemes were compared. In the first, a brief stimulation was presented and the subject moved their eyes after the end of the stimulation toward the phosphene's remembered location. In the second, a longer stimulation was presented and the subject moved their eye during the stimulation to track the phosphene's location. In the latter case, because the stimulation is continuously mapped based on eye position, an eye movement during the stimulation caused the phosphene to move. Results show that

subjects were able to conduct a smooth pursuit motion as a result of constant stimulation.

These experimental setups demonstrate that the integration of eye-tracking recording can be used to create the spatial map of a cortical visual implant.

PUPILLOMETRY

BENNETT LECTURE THEATRE 8 - 15:30- 15:50

What Does the Pupillary Light Response Tell Us About the Mechanisms Underlying Object-based Attention?

Yaffa Yeshurun, Felipe Luzardo and Wolfgang Einhäuser

University of Haifa, Israel
yeshurun@research.haifa.ac.il

Observers react faster when the target appears on the same object as a precue than when they appear on different objects. The ‘Attentional shifting’ account of such object-based effects underscores the higher cost of shifting attention between objects than within an object. The ‘Attentional spreading’ account attributes these effects to automatic spreading of attention along the object. We tested these accounts using attentional modulations of the pupillary light response (PLR). To test the spreading account, we employed objects composed of white-to-gray and black-to-gray luminance gradients. If attention spreads along the objects, pupil size should differ when the different types of objects are cued. To test the shifting account, we displayed a single object that could be black or white. A precue appeared inside or outside the object, and a target followed at the same or different location. If disengaging attention from a location inside the object is slower, then changes in PLR should start later when the precue appears inside the object and the target appears outside the object. We found evidence for the attentional shifting account but not for involuntary spreading of attention. Evidence for attentional spreading along the object emerged only when such spreading matched the observers’ goals.

PUPILLOMETRY

BENNETT LECTURE THEATRE 8 - 15:50- 16:10

Effects of Luminance and Arousal Related Baseline Amplitude on the Auditory Phasic Pupil Dilation Response

Andreas Widmann, Wolfgang Einhäuser, Nicole Wetzel and Phillip M. Alday

Leipzig University, Germany
widmann@uni-leipzig.de

The amplitude of the phasic pupil dilation response (PDR) is assumed to be independent of luminance-related baseline pupil diameter. There is, however, little empirical support for this assumption. Instead PDR amplitudes have been reported both to decrease and to increase with baseline diameter. Here, we systematically examined this relation in an auditory oddball paradigm including rare to-be-detected target and rare task-irrelevant novel sounds among frequent standard sounds. Display luminance was manipulated in six blocked conditions between 0.3 and 100cd/m2. The amplitude of the early parasympathetic inhibition-related principal component of the PDR decreased linearly with increasing luminance-related baseline diameter. The late sympathetic activation-related component showed an inverse u-shaped relationship with small PDR amplitudes at low and high luminance levels, but large amplitudes at medium luminance. For the spontaneous, non-luminance-related fluctuations of baseline pupil diameter, we observed decreasing amplitudes of both PDR components with increasing baseline. Importantly, this decrease was stronger for task-irrelevant novel sounds compared to task-relevant target sounds. We discuss possible explanations for the observed results in terms of the dynamics and mechanical properties of the pupillary system. Divisive baseline correction is discouraged in pupillometry as the underlying assumption does not hold. We propose regression-based baseline correction as feasible alternative.

PUPILLOMETRY

BENNETT LECTURE THEATRE 8 - 16:10- 16:30

Warming Up an Eye-tracker Alleviates System Drift in Gaze Position and Pupil Size

Richard Andersson and Kenneth Holmqvist

Tobii AB, Sweden
richard.andersson@tobii.com

We investigated system drift by tracking firmly mounted artificial eyes using five different eye trackers; SMI HiSpeed 240, SMI RED250Mobile, SR Research EyeLink 1000+, SR Research EyeLink II, and Tobii Pro Spectrum. The system drift in the gaze signal ranged from 0.03 to 2.71 degrees over two hours, depending on the eye tracker. A closer investigation of the eye images from the SMI HiSpeed 240 uncovered one potential mechanism for this, with images changing brightness over time. We show that this change in eye-camera images affects the contour estimation of the features in the eye image (pupil and corneal reflection), resulting in system drift not only for the gaze position but also for the pupil size. The artifactual changes in pupil size are on the same order as the largest cognitively driven pupil changes and could be a concern for experiments focused on temporal effects, such as learning, fatigue, or vigilance. The system drift was observed after starting the eye tracker from room temperature, but was considerably less noticeable after about an hour of use. Based on our findings, we recommend warming up eye trackers for at least one hour before recording data.

Author index

Abeles, Dekel, 175
Acarturk, Cengiz, 176
Acton, Jennifer, 90
Adedeji, Victoria, 25, 115
Adeli, Hossein, 106
Aispuriete, Inese, 216
Aizenshtein, Alina, 143
Albi, Ádám, 145
Alday, Phillip, 236
Alexander, Robert, 133
Alexeeva, Svetlana, 166
Aljassmi, Maryam, 37
Allen, Shanley, 167, 202
Almajed, Saad, 81
Alvarez, Marta, 75
Amiebenomo, Onyeka, 87
Andersson, Richard, 128, 131, 133, 237
Andrews, Sally, 35, 104
Angele, Bernhard, 119
Anisimov, Victor, 62
Antúnez, Martín, 33
Arco, Nicole, 40, 60
Areshenkoff, Corson, 42
Armstrong, Thomas, 129
Arnott, Stephen, 232
Arshad, Qadeer, 199
Artemenko, Elena, 54
Ashkenazi, Simone, 149
Atanasov, Petar, 223
Atkins, Andrew, 217
Babanova, Ksenia, 62
Bach, Michael, 91
Backhaus, Daniel, 142, 171
Bai, Xuejun, 24, 67, 68, 185, 188
Barach, Eliza, 224
Barber, Horacio, 33
Barrett, Doug, 92
Barton, Hayley, 64
Bartseva, Ksenia, 66, 158
Basvitch, Itay, 39
Beaton, Derek, 50, 232
Bellerby, Emily, 156
Benedek, Mathias, 76
Benjamins, Jeroen, 14, 133, 153
Benson, Valerie, 11, 48, 49, 162, 194
Bényei, Gábor, 147
Berg, Esther, 51
Bertram, Raymond, 120
Bhanap, Ruhi, 148
Biliotti, Carolina, 77
Bills, Katherine, 61
Binns, Malcolm, 232
Black, Sandra, 50, 232
Blignaut, Pieter, 133
Blohm, Stefan, 99
Blythe, Hazel, 109, 111, 156, 160
Bode, Lisa, 195
Böing, Sanne, 51, 146
Bompas, Aline, 122
Bonsu, Angela, 199
Booij, Linda, 231
Boudelaa, Sami, 37
Bramão, Inês, 182
Breen, David, 42
Breil, Christina, 177
Brenner, Eli, 170
Brien, Donald, 42, 50, 134, 231, 232
Brink, Teuni, 51, 146
Broek, Gesa, 139
Brouwer, Anne, 133
Bruland, Rouwen, 200
Bruner, Emiliano, 10
Brunet, Milca, 155
Bucci, Maria, 32
Burgart, Ksenia, 169
Burle, Boris, 30
Byrne, Sean, 77
Cabañas, Cynthia, 215
Cabestrero, Raúl, 52
Calancie, Olivia, 134, 231
Caliskan, Neslihan, 155
Carr, Jon, 26, 225
Caruso, Letizia, 94
Caspi, Avi, 233
Casteau, Soazig, 198
Castelhano, Monica, 9, 206
Castet, Eric, 30
Catrysse, Leen, 41
Cepero, Laura, 52
Chamorro, Eva, 71, 75
Chang, Min, 189
Chernova, Daria, 166
Chernova, Uliana, 46
Chernozatonskiy, Konstantin, 62

Chevet, Guillaume, 59
Chistofalos, Andriana, 224
Chiu, Tzu, 121
Christofalos, Andriana, 29, 60
Chuang, Lewis, 133
Clarke, Alasdair, 93, 94, 95, 96
Cleva, José, 71, 75
Coe, Brian, 42, 50, 134, 231, 232
Contreras, Mª, 52
Cooley, Frances, 192
Cordero, Ismael, 119
Cornelissen, Frans, 172, 229
Crawford, Trevor, 56
Crepaldi, Davide, 26, 225
Cristino, Filipe, 230
Cui, Lei, 205
Dagit, Gerald, 17
Dalmaijer, Edwin, 80, 129
Dalrympleke, Kirsten, 133
Dare, Zoya, 21
Dayan, Oryah, 213
Degg, Christopher, 91
Degno, Federica, 34, 186, 223
Deković, Maja, 13
Dewar, Jane, 50
Dias, Elisa, 29
Divjak, Dagmar, 203
Doorn, Andrea, 14
Dotor, Paulina, 71
Dragoy, Olga, 66, 158, 164
Dreimane, Lana, 216
Dreneva, Anna, 46, 143, 163
Drieghe, Denis, 109, 118, 121, 133, 208
Driessen, Joost, 170
Duke, Philip, 81
Dunn, Matt, 88, 90
Ede, Freek, 140, 179
Eekhof, Lynn, 100
Eggert, Thomas, 127
Einhäuser, Wolfgang, 235, 236
Eldar, Eran, 214
Emmorey, Karen, 191
Engbert, Ralf, 142, 171
Ennis, Fergal, 88
Erdmann, Mirjamaija, 112, 159
Erichsen, 85, 87, 88, 90
Erichsen, Jonathan, 85, 87, 88, 90
Ermolova, Maria, 46
Essmann, Lucas, 136
Faber, Myrthe, 101
Facevicova, Kamila, 150
Feldman, Laurie, 224
Fergusson, James, 90
Fernandez, Leigh, 167, 202
Ferrer, Xavier, 151
Filik, Ruth, 221
Finger, Elizabeth, 50, 232
Fink, Lauren, 137
Fisher, Donald, 211
Fleming, Roland, 73
Fokin, Danil, 99
Fontoura, Pablo, 19
Freedman, Morris, 50, 232
Friede, Anne, 116
Fu, Ying, 68, 185
Furman, Reyhan, 65
Gago, Maria, 10
Garcia, Clara, 75
Gava, Giuseppe, 199
Gesztesi, Gábor, 126
Ghasia, Fatema, 7, 86
Ghiani, Andrea, 170
Gilchrist, Iain, 45
Gill, Donna, 24
Glandorf, Domink, 27
Gnetov, Daniil, 57
Godwin, Hayward, 109, 208
Goktepe, Nedim, 196
Goldina, Sofya, 66, 158, 164
Gonzalez, Amelia, 71
Gottlob, Irene, 91
Grande, Pablo, 71, 75
Grappe, Amandine, 152
Green, Christine, 65
Greenberg, Shlomit, 175, 178, 184
Grimes, David, 42
Guan, Zheng, 187
Guerra, Claudia, 218
Guillaume, Chevet, 219
Guo, Meihua, 162
Gutzeit, Julian, 74, 83
Haan, Gera, 172, 229
Hadwin, Julie, 11
Häikiö, Tuomo, 112, 113, 159
Halbmayr, Helene, 45
Halonen, Sohvi, 113
Hao, Lisha, 189
Hautala, Jarkko, 105
He, Shiyu, 203
Hedge, Craig, 122
Heikkilä, Timo, 38, 205
Helmchen, Christoph, 195

Henderson, John, 210
Hessels, Roy, 13, 14, 128, 131, 133, 153, 154
Heuer, Anna, 181
Heutink, Joost, 172, 229
Hodgson, Timothy, 10
Höfler, Margit, 45
Hofmann, Markus, 31
Holleman, Gijs, 13, 14
Holmqvist, Kenneth, 133, 237
Hooge, Ignace, 13, 14, 128, 131, 133, 153, 154
Hoogerbrugge, Alex, 80, 146
Hoogerheide, Vincent, 139
Hout, Liz, 170
Howard, Philippa, 194
Hsieh, Ming, 165
Huang, Jeff, 42, 50, 231, 232
Huestegge, Lynn, 74, 83, 144, 177
Hughes, Anna, 96
Huijding, Jorg, 13
Hunt, Amelia, 93, 94, 95
Hyönä, Jukka, 38, 204, 205
Ibañez, Romualdo, 218
Indurkhya, Bipin, 176
Inhoff, Albrecht, 116
Ioannidou, Flora, 10
Irvine, Alexander, 129
Irwin, Amy, 94
Isel, Frédéric, 32
Iskra, Ekaterina, 164
Itti, Laurent, 134
Ivanov, Michael, 168
Ivanov, Michail, 44
Jainta, Stephanie, 84, 226
Jarodzka, Halszka, 41
Javitt, Daniel, 29
Jensen, Kåre, 152
Jia, Man, 141
Jian, Yu, 161
Jiskoot, Lize, 51
Jog, Mandar, 42
Johansson, Mikael, 182
Johansson, Roger, 182
Jones, Beth, 230
Joss, Joëlle, 84, 226
Judge, Jeannie, 24
Kaakinen, Johanna, 98, 112, 159
Kalabusova, Veronika, 150
Kalkan, Sinan, 176
Kalsi, Tami, 221
Kaltenbach, Katharina, 127
Kanerva, Oksana, 112, 159
Kaprielova, Anastasiia, 158
Kekunnaya, Ramesh, 228
Kemner, Chantal, 13, 154
Kerckhoffs, Andrienne, 41
Khan, Sarosh, 231
Kim, Junhui, 72
Kirkby, Julie, 23, 25, 61, 115
Kirkden, Richard, 34
Kirkpatrick, Ryan, 134
Kirtley, Clare, 227
Koenderink, Jan, 14
Koerfer, Krischan, 197
Kok, Ellen, 139
Koltsova, Olessia, 54
König, Peter, 136, 173
König, Sabine, 136
Konovalova, Anastasiia, 63
Korda, Ziva, 76
Korepanov, Andrew, 152
Korhummel, Carola, 18
Körner, Christof, 45, 76
Kosikova, Alisa, 44
Kreyenmeier, Philipp, 181, 200
Krieken, Kobie, 100
Kroell, Lisa, 181
Krutz, Alexander, 43
Kucharský, Šimon, 130
Kuht, Helen, 89
Kuo, Pin, 161
Kuperman, Victor, 57, 108
Kürten, Jens, 74, 83
Kwan, Donna, 42, 50, 232
Ladinskaya, Nina, 66, 158
Laks, Madison, 29, 60
Lan, Zebo, 162
Landmann, Eva, 177
Lang, Anthony, 42, 50, 232
Lange, Elke, 137
Langlois, Annick, 181
Lappe, Markus, 124, 197
Latanov, Alexander, 143
Latyshkova, Alexandra, 62
Laubrock, Jochen, 43, 110
Ledgeway, Timothy, 79
Lee, Charlotte, 109, 208
Levine, Brian, 42, 232
Li, Jie, 141
Li, Xingshan, 212
Li, Xiyuan, 190
Lin, Juntian, 141

Lin, Sunny, 165, 187
Liu, Baiwei, 140
Liu, Nina, 162, 190
Liu, Zhong, 183
Liversedge, Simon, 24, 34, 65, 67, 68, 111, 160, 185, 186, 188, 194, 209, 223
Lizunkova, Kseniya, 143
Lopukhina, Anastasiya, 66, 158, 164
Lou, Wendy, 232
Lowman, Michael, 61
Lu, Zijia, 67
Lüken, Malte, 130
Luzardo, Felipe, 235
Lyapunov, Ivan, 44
Lyapunov, Sergey, 44
Machner, Bjoern, 195
MacInnes, Joseph, 46
Mak, Marloes, 101
Malsburg, Titus, 28
Mancini, Régis, 30
Manning, Hannah, 114
Manoli, Athina, 11
Manzanera, Antoine, 123
Marcet, Ana, 119, 207
Marras, Connie, 42, 50, 232
Masellis, Mario, 42, 232
Maturi, Kinnera, 40
Mayas, Julia, 52
McArthur, Genevieve, 28
McCarty, Kristofor, 156
McCloy, Rachel, 125
McGowan, Victoria, 2, 58, 64
McGraw, Paul, 79
McIlreavy, Lee, 87, 88
McLaughlin, Paula, 42, 50
McLean, Rebecca, 91
McSorley, Eugene, 125
Meghanathan, Radha, 12
Meisinger, Elizabeth, 114
Meixner, Johannes, 110
Méndez, Laura, 52
Meng, Michael, 55
Mézière, Diane, 28
Milin, Petar, 203
Milledge, Sara, 111, 156, 160
Milligan, Sara, 33, 155
Minh, Alexandra, 151
Mirault, Jonathan, 191
Mironets, Sofia, 163
Miscena, Anna, 16, 22
Momose, Keiko, 82
Montoro, Pedro, 52
Moosa, Maleeha, 185
Morozov, Anna, 169
Müller, Katharina, 15
Munoz, Douglas, 134, 231
Murray, Christopher, 227
Nahari, Tal, 214
Nawrocki, Piotr, 176
Nejad, Ashkan, 172, 229
Niedra, Laima, 216
Niehorster, Diederick, 128, 131, 133, 153, 154
Nijboer, Tanja, 80, 146
Nikolaev, Andrey, 182
Nilsson, Mattias, 114
Nolte, Debora, 173
Norkina, Marina, 166
Nowakowska, Anna, 93, 94, 95
Nyström, Marcus, 128, 131, 153
Oberauer, Klaus, 148
Ohl, Sven, 180
Olah, Zoril, 80
Olkoniemi, Henri, 113
Orange, Jb, 42
Örbom, Saga, 133
Orenes, Isabel, 52
Ossandón, José, 228
Pagán, Ascensión, 2, 34, 190, 201
Pajkossy, Péter, 126, 145, 147
Palol, Marc, 173
Pamplona, Daniela, 123
Papenmeier, Frank, 17
Paranko, Birgitta, 205
Parker, Jessica, 135
Parmentier, Fabrice, 61
Parshina, Olga, 164
Paterson, Kevin, 2, 34, 37, 58, 64, 107, 190, 222
Pavlou, Katerina, 11
Pedersen, Nadia, 152
Pedrero, José, 71
Peltsch, Alicia, 42
Perea, Manuel, 37, 119, 207
Perkins, Julia, 134
Perra, Joris, 70
Pertzov, Yoni, 213, 214
Pescuma, Valentina, 26
Petrova, Tatiana, 168
Pexman, Penny, 113
Pieczykolan, Aleks, 144
Pikunov, Andrey, 62
Plunkett, Anna, 58

Polden, Megan, 56
Pollmann, Stefan, 12
Polonio, Luca, 77
Poos, Jackie, 51
Popelka, Stanislav, 138, 150
Postuma, Eva, 172, 229
Potthoff, Jonas, 47
Premeti, Aikaterini, 32
Prichard, Caleb, 217
Prieto, Antonio, 52
Proudlock, Frank, 85, 91
Qahtani, Fawziah, 222
Raab, Markus, 200
Radach, Ralph, 31, 36, 116, 220
Raettig, Anne, 177
Raettig, Tim, 83
Raijmakers, Maartje, 53
Rancz, Ede, 151
Ranta, Emilia, 98
Reichle, Erik, 28, 104, 107
Reitstätter, Luise, 18
Reuther, Josephine, 93
Riccaboni, Massimo, 77
Richards, Simon, 201
Riek, Heidi, 42, 134, 232
Roberts, Angela, 42
Rodán, Antonio, 52
Röder, Brigitte, 228
Rodrigues, Erica, 118
Rolfs, Martin, 181
Rölke, Andre, 31
Rosenbaum, David, 95
Rosenberg, Raphael, 18, 21, 22
Rosner, Agnes, 148
Ryan, Jennifer, 183
Sajjid, Asha, 65
Sanders, José, 100
Sauerberger, Kyle, 95
Saunders, Emily, 191
Saxena, Shreshth, 137
Scheepers, Christoph, 202
Schienle, Anne, 47
Schluppeck, Denis, 79
Schönfelder, Petra, 45
Schotter, Elizabeth, 33, 155, 192
Schroeder, Sascha, 27, 57, 69, 102
Schroeger, Anna, 200
Schütz, Alexander, 78, 196
Schwalm, Laura, 36, 220
Schwan, Stephan, 17
Schwetlick, Lisa, 142
Seelaar, Harro, 51
Seernani, Divya, 152
Senju, Atsushi, 215
Serebryakova, Margarita, 164
Shareef, Idris, 228
Sharif, Mishaal, 199
Shen, Kelly, 183
Sheridan, Heather, 29, 40, 60, 224
Sherman, Charli, 96
Sheth, Viral, 91
Shoshina, Irina, 44
Shovman, Mark, 169
Shurupova, Marina, 143, 163
Simola, Jaana, 98
Simon, Yuliya, 44
Slattery, Timothy, 25, 115
Smith, Daniel, 198
Smith, Tim, 215
Smorenburg, Matthew, 50, 134
Sniezynski, Bartlomiej, 176
Sobolewska, Aleksandra, 53
Souto, David, 2, 81
Spering, Miriam, 8, 181, 200
Spethmann, Sebastian, 43
Spieser, Laure, 30
Sprenger, Andreas, 195
Stamkou, Eftychia, 53
Stanovaya, Viktoriya, 44
Staroverova, Vladislava, 66, 158, 164
Startsev, Mikhail, 132
Stewart, Emma, 73
Stigchel, Stefan, 51, 80, 146
Stoffi, Falco, 77
Straube, Andreas, 127
Strauch, Christoph, 80
Strobl, Josef, 138
Strong, Michael, 50
Strother, Stephen, 50
Sturt, Patrick, 117
Sui, Longjiao, 157
Sultan, Minha, 138
Sumner, Petroc, 122
Takemoto, Ayumi, 216
Tan, Brian, 50
Tas, Caglar, 135
Tatiana, Petrova, 63
Tatler, Benjamin, 227
Taub, Keren, 184
Teixeira, Elisangela, 118
Telgmann, Leonie, 15
Terpilovskii, Maksim, 54

Thomas, Mervyn, 85, 89, 91
Thomas, Nikita, 90
Tilmatine, Mesian, 97, 103
Timmis, Matthew, 39
Tompson, Meyrem, 58
Tu, Zhanhan, 91
Tze, Natalie, 117
Ulianova, Taisiia, 54
Vakil, Eli, 149
Valtakari, Niilo, 154
Vasilev, Martin, 25, 61, 115
Vaughan, Phillip, 227
Vedenina, Olga, 66, 158
Veldre, Aaron, 35, 104
Vieira, Joao, 118
Visser, Ingmar, 130
Vitu, Françoise, 30, 106
Vorstius, Christian, 31, 116, 220
Vziatysheva, Victoria, 54
Wagner, Christoph, 17
Wagner, Ilja, 73, 78
Walcher, Sonja, 76
Walker, Robin, 198
Walter, Jasmin, 136
Wang, Jingxin, 92, 189
Wang, Mengsi, 24, 186
Wang, Shuangshuang, 188
Wang, Yongsheng, 190
Ward, Katherine, 88
Warrington, Kayleigh, 107, 189, 222
Waters, Lee, 39
Watson, Jonathan, 65
Watson, Tamara, 197
Wei, Lixin, 186
Weller, Lisa, 74
Wetzel, Nicole, 236
White, Brian, 134
White, Sarah, 34, 58, 107, 222
Widmann, Andreas, 236
Willems, Roel, 100, 101
Williams, Kirsten, 79
Wilson, Jessica, 152
Wolf, Christian, 124
Wolf, Kerstin, 152
Wolfer, Stephanie, 29
Wong, Roslyn, 35
Woodhouse, J, 87
Wu, Sarah, 58
Wu, Shihui, 107
Wynn, Jordana, 183
Xheladini, Agnesa, 167
Xie, Hongyu, 92
Xie, Mingqing, 117
Xu, Yuening, 49
Yan, Guoli, 48, 67, 68, 162, 188, 190
Yang, Fuyi, 49
Yep, Rachel, 42, 134
Yeshurun, Yaffa, 235
Yoshida, Takako, 72
Yoshinari, Yuko, 174
Yu, Lili, 28, 104
Yuan, Xiaoyuan, 162
Zang, Chuanli, 24, 65, 67, 68, 160, 185, 186, 188
Zdorova, Nina, 66, 158, 164
Zehngut, Amit, 175
Zelinsky, Gregory, 106
Zemblys, Raimondas, 132
Zentall, Thomas, 95
Zerbib, Véronique, 59, 70, 219
Zerr, Paul, 228
Zhang, Kuo, 189
Zhang, Li, 48, 49
Zhang, Manman, 186
Zhang, Qi, 141
Zhang, Yu, 141
Zhang, Zhichao, 65
Zhigulskaya, Daria, 62
Zhou, Li, 49
Zonneveld, Anne, 140

Exhibitors and Sponsors

Gold Sponsors

Silver Sponsors

Bronze Sponsors
